# Pulsed cavitation ultrasound assisted delivery of cardamom, pistacia and laurel encapsulated micelles nanoparticles for sono-photodynamic lymphoma in vitro and in vivo treatment

**DOI:** 10.1007/s10103-025-04387-x

**Published:** 2025-03-24

**Authors:** Samir Ali Abd El-Kaream, Safia Ali Hussein Hamoda, Sohier Mahmoud El Kholey, Awatef Mohamed El-sharkawy

**Affiliations:** Alexandria University, Alexandria, Egypt

**Keywords:** Lymphoma, Cardamom, Pistacia, Laurel, Micelle nanoparticle, Sono-photodynamic

## Abstract

Sono-photodynamic therapy (SPDT) has attracted a lot of interest as a cutting-edge therapeutic strategy in the field of cancer treatment. The essential part of SPDT is the sensitizer, which under laser photon and pulsed cavitation ultrasound sono-irradiation may transform sono and photo- energy into cytotoxic molecules. Photon absorption, targeting, penetration, and oxygen dependence remain challenges in sono- -photosensitizer (SPs) design. Rapid advancements in material science have prompted the creation of several SPs that create cytotoxic species with great selectivity, safety, and noninvasiveness for the treatment of tumors. The current study aims to provide an advanced method of activated cancer treatment by using pulsed cavitation to assist the delivery of cardamom, pistacia and laurel conjugated micelles nanoparticles (CPL-Micelle NP) for the sono-photodynamic lymphoma in vivo and in vitro treatment. Human lymphoma cells (U-937) were used in the in vitro study, and the in vivo application groups of the study protocol were Swiss albino mice treated with 9,10-Dimethyl-1,2-Benzanthracene (DMBA) only; they were not given any treatment to induce lymphoma. The study treatment protocol started only after lymphoma induction, and involved daily administration of CPL-Micelle NP as SPDT sensitizer whether or not to be exposed to photo- (IRL) or sono- (US) or a combination of them for three minutes for a period of two weeks. Indicated that Micelle NP is a useful CPL delivery mechanism that targets lymphoma cells directly. Furthermore, CPL-Micelle NP is a promising SPS that, when used in conjunction with SPDT, can be very effective in in vitro treating lymphoma-U-937 (in a dose-dependent manner cell viability declined, an increase in the cells population during the S and G2/M phases indicates that the cell cycle was arrested, and an increase in cell population in the Pre-G, autophagic cell death, as well as necrosis and early and late apoptosis, indicate that cell death was induced) and DMBA-Lymphoma-induced mice in vivo (induced antiproliferative genes, repressed antiangiogenic and antiapoptotic genes), successfully slowing the growth of tumors and even killing cancer cells, as well as lowering oxidative stress malondialdehyde (MDA), improving the functions of the kidneys, liver, and enzymatic and non enzymatic antioxidants. SPDT, the photo- or sono- chemical CPL activation mechanism, and the antioxidant capacity of non-activated CPL can all be linked to this process. On the bases of the findings, CPL-Micelle NP shows a great promise as a novel, efficient selective delivery system for localized SPDT-activated lymphoma treatment.

**Supplementary Information:**

The online version contains supplementary material available at 10.1007/s10103-025-04387-x.

## Introduction

Hematological malignancy, such as lymphoma, starts in the lymph tissues and tends to spread to other organs. Lymphoma is a broad category of cancer. The two primary varieties of lymphomas are non-Hodgkin and Hodgkin. [1–3] Lymphoid malignancies are complicated diseases with unique biological characteristics, clinical manifestations, and therapeutic outcomes. There are very few effective medications for treating lymphoma, making it a tough disease to treat. Managing these types of tumors is difficult since they are highly recurrent and conventional approaches such as surgery and local treatment are impractical. Furthermore, the cytotoxicity of conventional cancer treatments and chemotherapy drugs causes detrimental side effects that impair the ability to control the malignant growth. It has proven difficult to find new, effective, selective, and less toxic treatment compounds for lymphomas. Ongoing research and technology improve our knowledge of these cancers and how to treat them, leading to more individualized methods of diagnosis and treatment. Consequently, in an effort to address the main problems with conventional cancer treatment, researchers are looking for effective treatments in complementary, alternative, and herbal medicine. [3, 4] One of this alternatives is photodynamic therapy (PDT) has gained popularity in recent years as a means of treating many tumors, either as a stand-alone treatment or in conjunction with other anticancer therapies. PDT involves giving a medication known as a photosensitizing substance (PS) and then shining light on the target area at a wavelength that matches the PS's absorbance to cause a range of biological effects. [5, 6] To address the shortcomings of PDT, sonodynamic therapy (SDT) was evolved. [7] The development of novel approaches to treat malignant tumors is underway, with the goal of improving the overall prognosis of those who suffer from this illness. Sono-photodynamic therapy (SPDT) is one of these; it is a therapeutic approach based on the fact that medications become of much more detrimental impact when combined with an ultrasonic wave and photo-irradiation of the tumor tissue. [8–10] A possible technique to address some of the shortcomings of traditional therapy approaches for lymphoid malignancies is nano-based natural sensitizers combined with SPDT. Using nano-delivery system locally activated therapy in the treatment of lymphoma would be a significant tactic to manage the dynamic process of the tumor, overcoming resistance and increase survival. [3, 11]

Using medicinal plants based sensitizers offers a different approach to reducing the side effects of synthetic drugs. For an extended period, the plants have been utilized to treat cancer owing to their chemical components of therapeutic significance. The human body experiences physiological effects from these chemicals. Clinical trials and phytochemical research have demonstrated that numerous herbs have anti-tumor potential against different types of cancer. Herbal remedies for lymphoma are widely used in impoverished nations. [9–15]

The development of new targeted treatments has revolutionized with the introduction of innovative targeted therapies. Despite the fact that these treatments are more precise than the conventional chemotherapy method, they are nevertheless associated with toxicities linked to dosage and drug resistance. Nano medicine has shown itself to be a highly effective stand-in for increasing the bioavailability of therapeutic components and reducing the deleterious impact of medications in recent decades. Therapeutic agents can be specifically delivered to cancerous cells through the use of Nano therapeutics, which can also transport drugs to the intended locations. [3, 4, 11]

The most widely used spice in the world is cardamom, a dietary phytoproduct whose advantageous health qualities are drawing increasing attention. Traditional therapeutic application of cardamom [Elettaria Cardamomum (L.) Maton. (Family: Zingiberaceae)] Include the treatment of tooth and gum infections, nausea, diarrhea, cataracts, asthma, and problems of the heart, stomach, and kidneys. The biological activity of cardamom and its polyphenols, which include antioxidant, anti-inflammatory, anti-tumor, and metabolic regulation properties, has been shown in numerous researches. [16] Pistacia lentiscus L., commonly referred to as Pistacia, is a member of the Anacardiaceae family, which is a global family with over 600 species and over 70 genera. Since Pistacia plant parts have hypoglycemic, antiatherogenic, cytoprotective, hepatoprotective, antipyretic, anti-inflammatory, antiulcerogenic, antifungal, antibacterial, antiviral, antiparasitic, antigenotoxic, antimutagenic, antioxidant, and anticancer capabilities, along with stimulant and diuretic capacity, they have been chemically characterized and used to treat a variety of human ailments. [17] Laurel, or Laurus nobilis L., is a native of the Mediterranean region. Because it has so many different medical uses, laurel is extremely important. It has long been used to treat digestive issues like eructation, flatulence, and altered digestion. According to previous reports, antifungal, antibacterial, and anticancer active compounds are also abundant in Laurel. [8–19]

The present study offers a different approach to reducing the harmful effects of traditional therapies. Additionally, novel approaches to the treatment of malignant tumors are currently being researched and developed, that raises the standard of patient survival. The main aim of this study is to propose an innovative investigation into pulsed cavitation ultrasound assisted delivery of cardamom, pistacia and laurel conjugated micelles nanoparticles for in vitro and in vivo sono-photodynamic lymphoma treatment. There has been no enough research has been done to determine how cardamom, pistacia and laurel conjugated micelles nanoparticles (CPL-Micelles NP) works as anticancer or the potential uses for it as a SPS for SPDT.

## Materials and methods

### Materials

All of the chemicals used were obtained from commercial sources and didn't need to be further purified. Poloxamer 188 was obtained from (Sigma-Aldrich) CAS No. 9003-11-6. Cardamom, pistacia and laurel were acquired from Tazarin LTD 11576573 on Shanghai, China's Aladdin Industries Inc. Cairo Biodiagnostic, Egypt provided kits measuring antioxidant total capacity (TAC) [20], glutathione peroxidase -S-transferase, and reductase (GPx, GST, GSH,GR) [21, 22], superoxide dismutase (SOD) [23], catalase (CAT) [24, 25], urea, aminotransferases (aspartate AST, alanine ALT), and creatinine. [26] Lipid peroxide (Malondialdehyde; MDA) [27] was also purchased [BioVision Catalog # K739-100, #K274-100, #K761-100, #K263-100, #K335-100 and #K773-100, Sigma Catalog #MAK179, #MAK080, #MAK052, #MAK055, #MAK089 and #MAK089 respectively]. The cDNA H-minus ABT synthesis kit #ABT009, the qPCR WizPure.™ (SYBR) Master #W1711, #K1652, and the total RNA (spin column) mini ABT extraction kit #ABT002 were purchased from Applied Biotechnology and Wizbiosolutions Inc., respectively. [28] WST-1 assay Abcam® #ab155902 kit [29], Annexin V-FITC / PI's apoptosis Abcam Inc #ab14085 detection kit were purchased from Abcam Inc., Cambridge Science Park, Cambridge, UK. [30]

### Methods

The complete investigation and inquiry process was conducted in compliance with all relevant laws and guidelines.

#### CPL-Micelles NP preparation and characterization

**Fig. 1:**
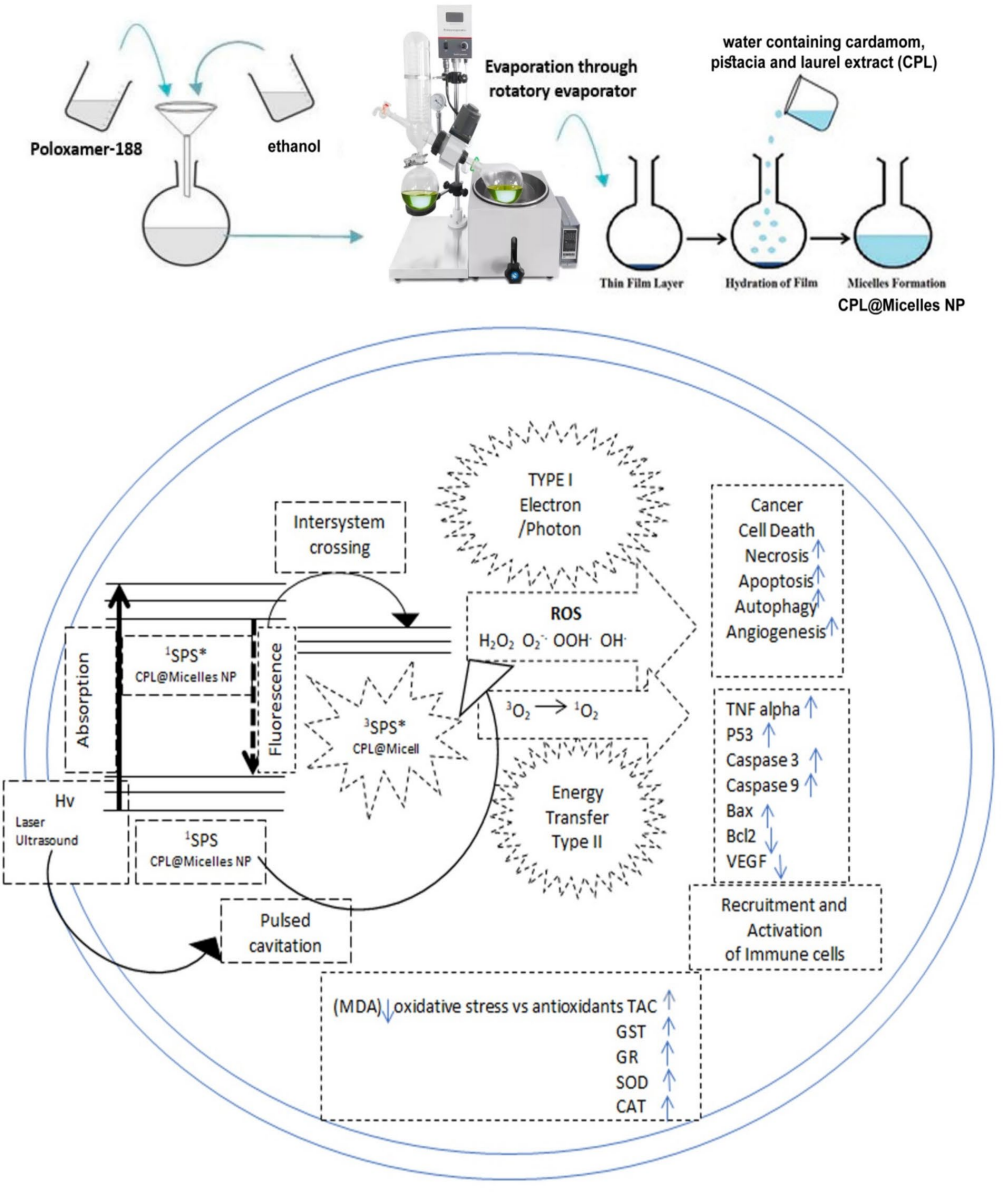
Schematic illustration of SPDT and CPL-Micelles NP synthesis

CPL-Micelles NP was applied as SPS in the present study Fig. (1). The Micelles NP were synthesized and conjugated to CPL as following; 100 mg of poloxamer 188 powder were dissolved in a quantity of 10 ml of ethanol contained in a round bottom flask, with gently shaking. The mixture was then charged in a rotary vacuum evaporator with a vacuum controller, evaporated to dryness at 50 °C and 100 rpm. The dried lipid film was hydrated by drop wise addition of 10 ml of water containing the filtrated of 10 mg of cardamom, pistacia and laurel extract (CPL) (1 mg/ml concentration) in at 35 °C and 100 rpm for 30 min. [31] CPL-Micelles NP was characterized by measuring its size and shape, CPL-Micelles NP was examined using a particle size analyzer, Fourier transform scanning infrared spectroscopy (FTIR), absorbance and photoluminescence scanning spectrometry (UV/Visible, PL), scanning energy dispersive and diffraction X-ray (EDX, XRD), transmission and scanning electron (TEM, SEM) microscopy, and zeta potential. Lymphoma-U-937 cell lines treated with CPL-Micelles NP and the DMBA-Lymphoma-induced mice groups (ip injected) were given nine to twelve hours to incubate before being subjected to PDT and/or SDT for a period of two weeks.

### Ethics statement

The requirements of the Institutional Committee for the Care and Use of Animals (IACUC); (ALEXU-IACUC; Code No.0122232221), as well as the policies of the European Convention for the Protection of Vertebrates Used for Experimental and Scientific Purposes regarding animal care and use in research and teaching are followed in all animal experiments described in this study. Every attempt was made to lessen the animals' suffering, and when necessary, authorized anesthetic techniques were used.

### In vitrostudy

#### **1. (Control positive group)** 

Lymphoma cell (U-937) line was kept in a drug-free environment and was not treated.

#### **2. (non activated CPL-Micelles NP group)**

Lymphoma cell (U-937) line was received 0.041 µl dissolved CPL-Micelles NP in PBS solely. 

#### 3. (Laser subjected group)

Lymphoma cell (U-937) line was subjected to laser for 3 min. 

#### 4. (Laser CPL-Micelles NP activated group)

Lymphoma cell (U-937) line was received 0.041 µl CPL-Micelles NP and subjected as group 3 to laser.

#### **5. (Ultrasound subjected group)**

Lymphoma cell (U-937) line was subjected to ultrasound for 3 min. 

#### 6. (Ultrasound CPL-Micelles NP activated group)

Lymphoma cell (U-937) line was received 0.041 µl CPL-Micelles and subjected as group 5 to ultrasound. 

#### 7. (Combined ultrasound and laser group)

Lymphoma cell (U-937) line was subjected to ultrasound and laser for 3 min.

#### **8. (Combined ultrasound and laser CPL-Micelles NP activated group)**

Lymphoma cell (U-937) line was received 0.041 µl CPL-Micelles NP, and subjected as group 7 to laser and ultrasound.

### In vivostudy

Ninety Albino mice, weighing 15 ± 1.5 g and aged 28 ± 1 days, were obtained from Alexandria University- Faculty of Agriculture's animal house. Experimental animals in suitable cages were housed with 12 h light—wake / dark—sleep cycles and 26 ± 0.5 °C temperature. The experimental animals had full access to tap water and were given a constant pellet diet. The drug was administered after the experimental animals had acclimated for a week. To put it briefly, the experimental animals were grouped into mouse nine groups of ten each: 

#### **1. (Control negative group)**

Normal healthy untreated mice were kept untreated. 

#### 2. (Lymphoma control positive group)

mice were orally administered only a dose of 50 mg/kg bw 9,10-Dimethyl-1,2-Benzanthracene (DMBA) to induce lymphoma and kept untreated. 

#### 3. (non activated CPL-Micelles NP group)

Lymphoma induced mice were administered a 0.041 ml daily dose of CPL-Micelles NP dissolved in PBS solely. 

#### 4. (Laser group)

 Lymphoma-induced mice were irradiated with laser every day for three minutes for two weeks. 

#### 5. (Laser CPL-Micelles NP activated group)

Lymphoma-induced mice were administered a 0.041 ml daily dose of CPL-Micelles NP and subsequently irradiated with laser every day for three minutes for two weeks. 

#### 6. (Ultrasound group)

Lymphoma-induced mice were irradiated with ultrasound every day for three minutes for two weeks. 

#### 7. (Ultrasound CPL-Micelles NP activated group)

Lymphoma-induced mice were administered a 0.041 ml daily dose of CPL-Micelles NP and subsequently irradiated with ultrasound every day for three minutes for two weeks. 

#### 8. (Combination ultrasound and laser group)

Lymphoma-induced mice were subjected to, ultrasound followed by laser photon every day for three minutes for two weeks.

#### 9. (Combined ultrasound and laser CPL-Micelles NP activated group)

Lymphoma-induced mice were administered a 0.041 ml daily dose of CPL-Micelles NP, and were irradiated with both laser and ultrasound every day for three minutes for two weeks.

### Instruments

#### Laser irradiation

Before the experimental animals were exposed to the laser, doses of (100, 10 mg/kg bw) ketamine and xylazine were used to put them to sleep. Around the tumor, there was no hair by shaving. The mouse was positioned facing the board. The probe of laser was exactly positioned almost above the tumor, and the groups were treated to laser therapy for three minutes under previously mentioned certain conditions. The mice were in the dark kept after PDT in order to prevent irritation of skin. The tumor in mice was irradiated (10 mW and 0.43 J/cm2) using a high-tech laser type LAS 250-infrared diode from Fujimed, China. It has a 50 W output peak and a wavelength of 600–904 nm at frequencies between 1 and 7000 Hz.

#### Ultrasound irradiation

Before the experimental animals were exposed to the ultrasound, doses of (100, 10 mg/kg bw) ketamine and xylazine were used to put them to sleep. Around the tumor, there was no hair by shaving. The mouse was positioned facing the board. The probe of ultrasound was exactly positioned almost above the tumor, and the groups were treated to ultrasound therapy for three minutes under previously mentioned certain conditions; utilizing an electronic tube, an ultrasonic (Shanghai, CSl China, Model822) generates an alternating electric current oscillation with a frequency of 0.8 MHz. Using an ultrasonic transducer, the gadget transforms its power output into mechanical ultrasonic energy. Ultrasonic mechanical energy can be used to create a beam density of 0.5–3 W/cm^2^. With a 1/3 duty ratio, 1000 Hz pulse frequency, and a 0.15–1 W/cm^2^ power density average range, this device can provide power in the range of 0.5–3 W/cm^2^. It has the ability to function in continuous and pulsed modes.

Upon the experimental protocols were completed, the experimental animals were euthanized by 5% isofurane (overdose) inhaling, and to ensure euthanasia cervical dislocation was conducted. 60 s later when the mice had white eyes and no longer heartbeat. Following the dissection, entire blood and sera were extracted from blood samples. After centrifuging a portion of the blood at 1000xg for ten minutes, and at −20 °C the sera separated were stored until analysis. The blood remaining sample was taken and at −80 °C stored until the identification and evaluation of gene relative expressions was started. After then it was reconstituted into another vial containing an RNA later solution from the vial containing EDTA. Furthermore, lymphoma tissues were removed immediately and washed in cold saline, punctured with a needle within a vial, and stored in a 10% formalin/saline solution for histopathological analysis.

#### **Cell culture** 

Human lymphoma cell U-937 was supplied by the American Type Culture Collection (ATCC). The cells were cultured in DMEM media supplemented with 10% heat-inactivated foetal bovine serum, 100 units/mL penicillin, and 100 mg/mL streptomycin. The cells were kept at 37 °C in a humidified 5% CO_2_ (v/v) environment.

### Test for cell viability and cytotoxicity

The CPL-Micelles NP cytotoxic effect against U-937 cells was evaluated by the WST-1 assay using the Abcam® WST-1 ab155902 kit, the cytotoxic impact of CPL-Micelles NP against U-937 cells was evaluated. 96-well plates were seeded with aliquots of 50 μL (5 × 10^3 cells) cell suspension, and the plates were cultured in complete medium for a full day. A second aliquot of 50 μL medium with CPL-Micelles NP at successive concentrations (1000, 100, 10, 1.0, and 0.1 μM) was then added to the cells for treatment. Following a full day of exposure to CPL-Micelles NP, cells were treated with 10 μL of WST-1 reagent, and an hour later, at 450 nm, A BMG microplate Omega reader (Ortenberg, Germany) LABTECH.®-FLUOstar was applied to detect the absorbance. [29]

### Flow cytometry assay

#### **Cell cycle analysis** 

Trypsinization was used to gather 10^5^ U-937 cells after a 24-h following various modalities treatment, in presence and absence of CPL-Micelles. Then, the cells were cleansed in PBS twice, which was extremely cold (pH 7.4). After being re-suspended in 60% ice-cold ethanol two milliliters, the cells were fixed at 4 °C for an hour. The fixed cells were rinsed in PBS (pH 7.4) repeatedly after being suspended in 1 mL of PBS containing RNAase A 50 µg/mL and propidium iodide (PI) 10 µg/mL. The cells were examined after 20 min at 37 °C of dark incubation. The cells' DNA content was assessed using flowcytometry analysis utilizing a Novocyte™ ACEA flowcytometer (Biosciences Inc., USA) fitted with a (λex/em 535/617 nm) FL2 signal detector. For all samples, a total events of 12,000 were gathered. With the use of Novo ACEA Express™ software, the cell cycle distribution was identified [32].

#### Analysis of apoptosis and necrosis Annexin V-FITC / PI's

The Abcam Inc. detection kit, Annexin V-FITC apoptosis (Cambridge, UK) was used in conjunction with two fluorescent channels flowcytometry to identify the necrosis and apoptosis cells populations. Trypsinization was used to isolate the 10^5^ total U-937 cells after a 24-h treatment period utilizing different modalities both with and without CPL-Micelles. The cells twice were washed in ice-cold PBS pH 7.4. As directed by the manufacturer, cells were treated at room temperature for 30 min and in the dark with 0.5 ml of Annexin V-FITC/PI solution. After staining, the Novocyte™ ACEA flowcytometer was used to measure the FITC and PI fluorescence signals using signal FL1 and FL2 detectors (λex/em 488/530 nm and 535/617 nm for FITC and PI). For all samples, a total events of 12,000 were gathered. Using Novo Express.™ ACEA software, positive PI and/or FITC cells were quantified and calculated via quadrant analysis. [30]

#### Autophagy analysis

Autophagic cell death was measured using flowcytometric analysis and the lysosomal dye acridin orange (AO). Trypsinization was employed to isolate U-937 cells (10^5^ cells) following a 24-h trial of various modalities, in presence and absence of CPL-Micelles. After that, the cells were cleansed in (pH 7.4) PBS twice that was extremely cold. After the cells were stained with (10 µM) AO, they were incubated for 30 min at 37 °C in the dark. Following labeling, cells were injected using a Novocyte ACEA flowcytometer, and the signal FL1 detector (λex/em 488/530 nm) was used to analyze the AO fluorescence signals. Novo Express™ ACEA software is used to measure net fluorescence intensities (NFI) based on 12,000 events collected for each sample [33].

### Molecular, biochemical, and histological analyses

#### Liver and kidney enzymes biochemical analysis

According to Burtis et al. (2008), commercial kits were used to assess the liver (AST and ALT) and kidney (creatinine and urea). [26]

#### Assessment of the antioxidant and oxidative stress indicators

The serum antioxidant and oxidative stress markers levels were evaluated using commercial kits. Lipid peroxidation is assumed to be indicated by levels of malondialdehyde (MDA). [27] SOD activity [23] glutathione- peroxidase, S-transferase and reductase (GPx, GST, GSH, GR) activities. [21, 22] Catalase activity. [24, 25] Total antioxidant activity (TAC). [20]

#### **Assessment of the relative gene expressions of TNF alpha, Caspase (3, 9), Bcl-2, Bax, p53, and VEGF** 

To find the expression of the genes, qRT-PCR was utilized. The Total RNA mini spin column ABT extraction kit was used to isolate total RNA from blood samples in accordance with the instructions. Purity was assessed by measuring the ratio of absorption at 260 to 280 nm, which was often higher than 1.8. Using a one-step RT-PCR reaction, the cDNA was synthesized as instructed by the cDNA ABT H-minus synthesis kit. Wiz Pure™ qPCR (SYBR) Master with ROX Dye was applied for qRT-PCR. A (20 µL) reaction mixture including 0.25 µM target gene primers and 5 µL of template C-DNA, P53-R is TGGAATCAACCCACAGCTGCA, and P53-F is CTGTCATCTTCTGTCCCTTC; Caspase3-R is AAATGACCCCTTCATCACCA, and Caspase3-F is TGTCATCTCGCTCTGGTACG; Caspase9-F is AGTTCCCGGGTGCTGTCTAT, and Caspase9-F is GCCATGGTCTTTCTGCTCAC; TNF-α-R is TGAGATCCATGCCGTTGGC, and TNF-α-F is CACGTCGTAGCAAACCACC; Bax-R is CCAGTTCATCTCCAATTCG, and Bax-F is CTACAGGGTTTCATCCAG; Bcl2-F is GTGGATGACTGAGTACCT, and Bcl2-R is CCAGGAGAAATCAAACAGAG; VEGF-R is TTTCTCCGCTCTGAACAAGG and VEGF-F is AAAAACGAAAGCGCAAGAAA, β-actin-R (CTCTCAGCTGTGGTGGTGAA) and β-actin-F (AGCCATGTACGTAGCCATCC) were used in qRT-PCR (10 µL) Sybr Green. The qRT-PCR PikoReal Thermo Scientific apparatus (PR0241401024) was run in 35 cycles, consisting of 10 s at 95 °C, 10 s at 55 °C, and 5 s at 72 °C. At 95 °C, the first denaturation process lasted for five minutes. Equation 2^−ΔΔCt^ was utilized to compute the fold difference between each gene expression, while the β-actin mRNA levels from the same sample were used for comparison. [28]

#### **Histopathology lymphoma tissue analyses** 

After being collected, the lymphoma samples were fixed by dipping them into a formalin/saline solution (10%), embedded in paraffin, and sectioned. The degree of histological changes in the lymphoma tissue was assessed using the staining dye haematoxylin and eosin (H&E). Every slide was examined under a light microscope, and images were captured. [34, 35]

#### **Regarding to statistical analysis** 

Mean ± standard deviation (SD) was the data formatted in. The statistical variances in the data were verified using one-way analysis of variance (ANOVA). When determining statistical significance, P values less than 0.05 were used. The groups were compared using the post hoc analysis tool of SPSS 25.0.

## Results

### CPL-Micelles characterization

**Fig. 2:**
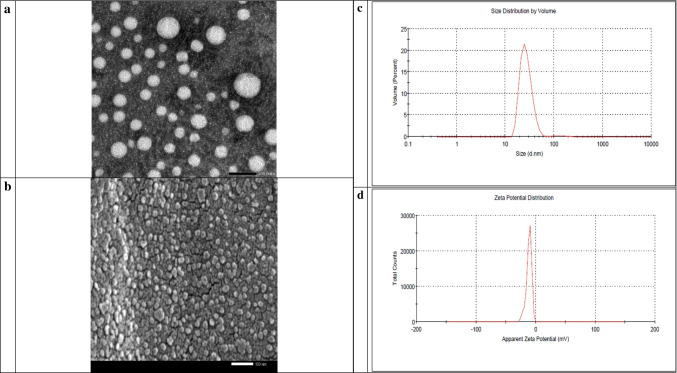

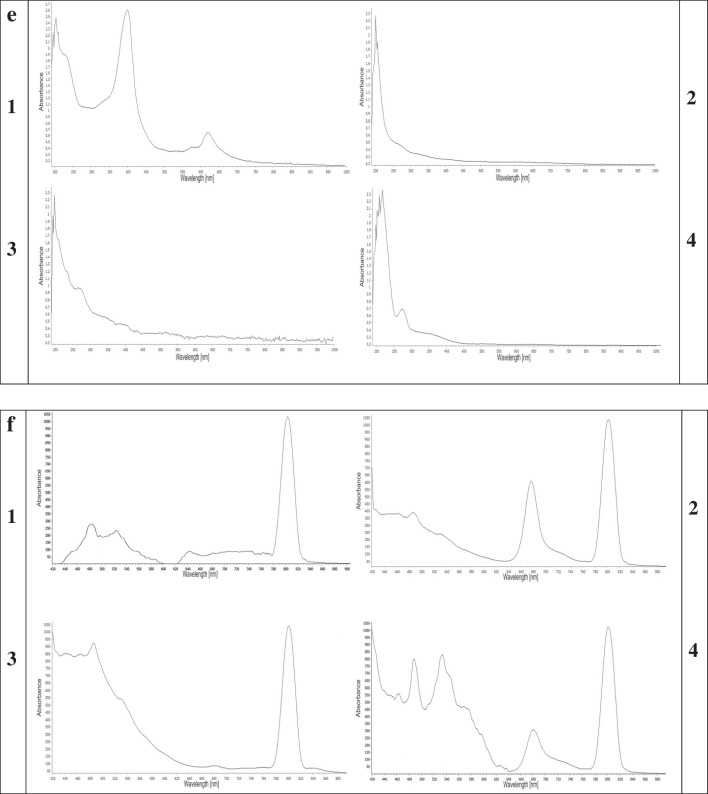

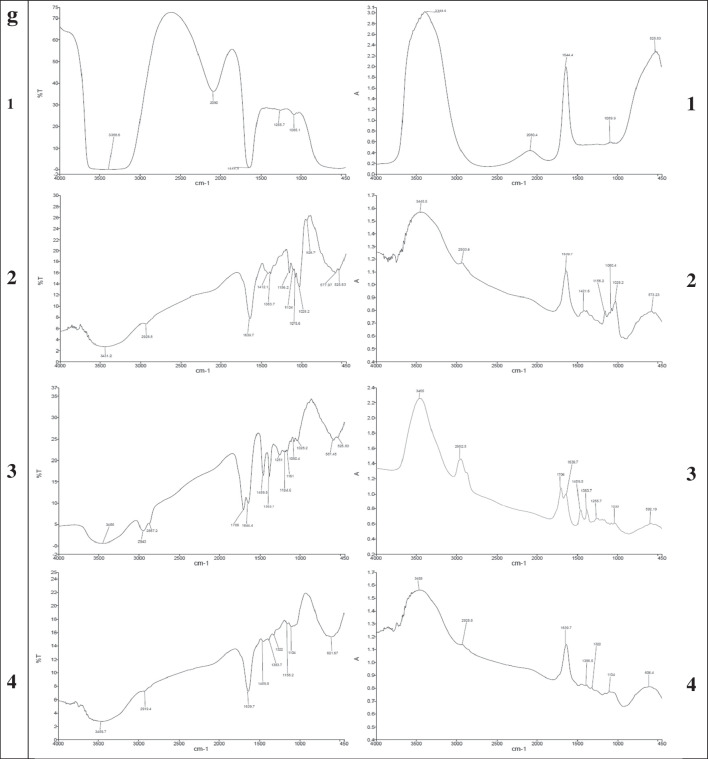

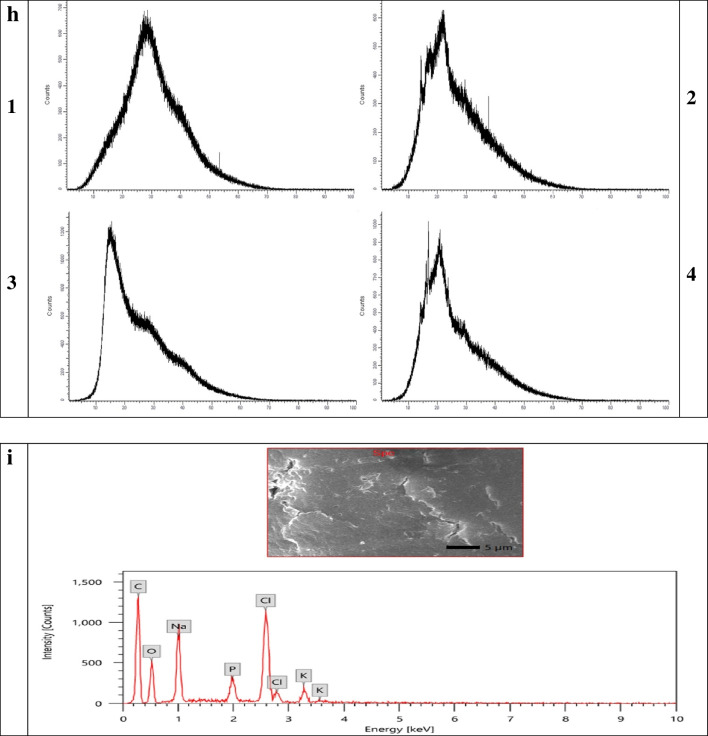
CPL-Micelles NP Characterization; **a**-**d** (TEM, SEM, particle size, zeta potential of CPL-Micelles NP), **e**–**h** (UV–Vis spectra, PL, FTIR transmittance and absorbance, XRD of (1. CPL-MicellesNP, 2. Cardamom, 3. Pistacia, 4. Laurel), **i**. EDX of CPL-Micelles NP

In accordance with Fig. (2) the particle size of CPL-Micelles mapped by TEM and particle size analyzer Fig. (2a,c), SEM results showing surface morphology of the nanocomposites illustrating the conjugation of CPL to Micelles NP Fig. (2b), zeta potential Fig. (2d), characteristic (UV–Visible) absorption peaks for CPL before and after conjugation with Micelles NP Fig. (2e), characteristic (PL) photoluminescence peaks for CPL before and after conjugation with Micelles NP Fig. (2f), the characteristic FTIR bands found that match the vibrations of the functional groups that are present in CPL before and after conjugation with Micelles NP represented on Fig. (2 g); FTIR function groups peaks for cardamom ( 3431, 2928, 1639, 1412, 1239, 1156, 1075, 1028 cm^−1^), Pistacia (3455, 2943, 2867, 1644, 1459, 1383, 1251, 1184, 1161,1050, 1028 cm^−1^) and Laurel (3459, 2919, 1644, 1539, 1459,1383, 1322, 1251, 1156, 1104 cm^−1^) and Micelle NP (3388, 1544, 1089 cm^−1^); the band at 3459- 3431 cm^−1^ is described to –OH group, while those at 2943–2919 cm^−1^ is assigned to the C–H stretching vibration of aliphatic CH_3_ and CH_2_ groups. The band at 1644–1639 cm^−1^ is attributed to the stretching vibration of the C = O bond. The band at 1459–1412 cm^−1^ is attributed to the C–C phenyl ring stretching. The band at 1383–1322 cm^−1^ is attributed to CH_3_ groups. The band at 1251–1239 cm^−1^ is due to C–O stretching. 1161-1156 cm^−1^ is attributed to the CC vibration of the molecule skeleton. 1104–1050 cm^−1^ is attributed to the -OH and -C–O–C group vibrations. 1028 cm.^−1^ is attributed to the C = C stretching. as well as XRD the characteristic peaks observed at 2θ value of Micelles NP broad band at 30° and CPL before and after conjugation with Micelles NP (C; 14°, 17°, 18°, 22°, 23°, 28°, 29°, 38°, P; 13°, 15°, 16°, 17°, 18°, 19°, 26°, 27°, 32°, 39°, L; 14°, 16°, 17°, 21°, 22°, 24°, 28°, 29° indicating high crystallinity and phase purity Fig. (2 h), and EDX elemental analysis illustrating the presence of C, K, N, Fe, S in the Micelles NP in correct state alongside with CPL content Fig. (2i), demonstrating the correct and well preparation of Micelles NP as well as high entrapment efficiency; correct conjugation and nanocomposite formation in nanoscale; small particle size, narrow size distribution. The overall results indicated that CPL-Micelles nanocomposite was properly synthesized in compliance with earlier research. [20]

### Cytotoxicity of PDT, SDT and SPDT on the lymphoma U-937 cell line in (CPL-Micelles) presence and absence

**Fig. 3:**
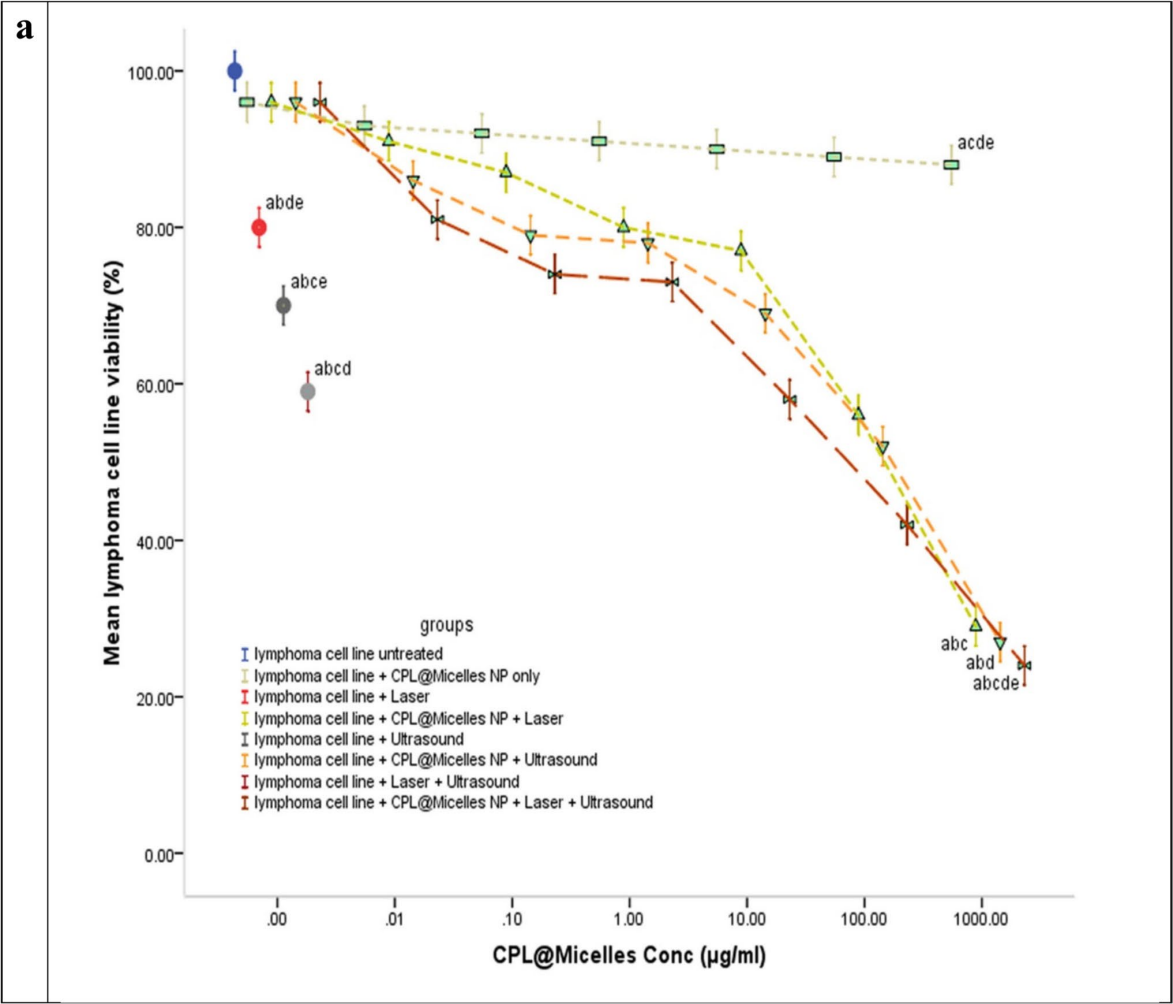

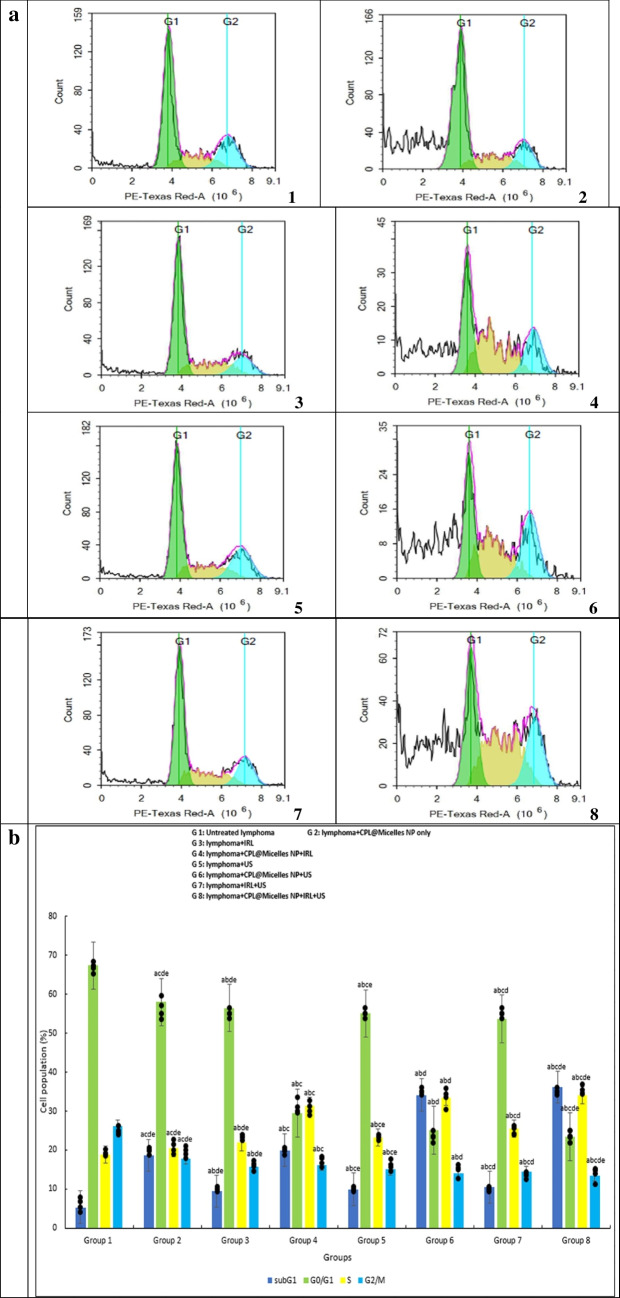

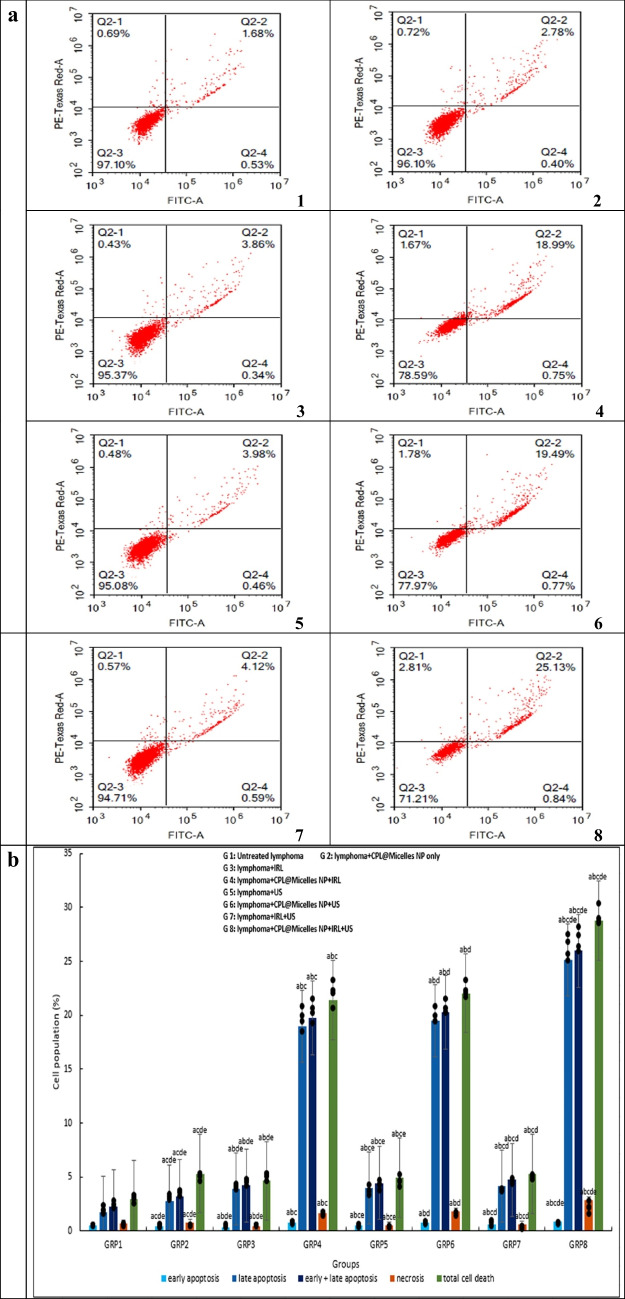

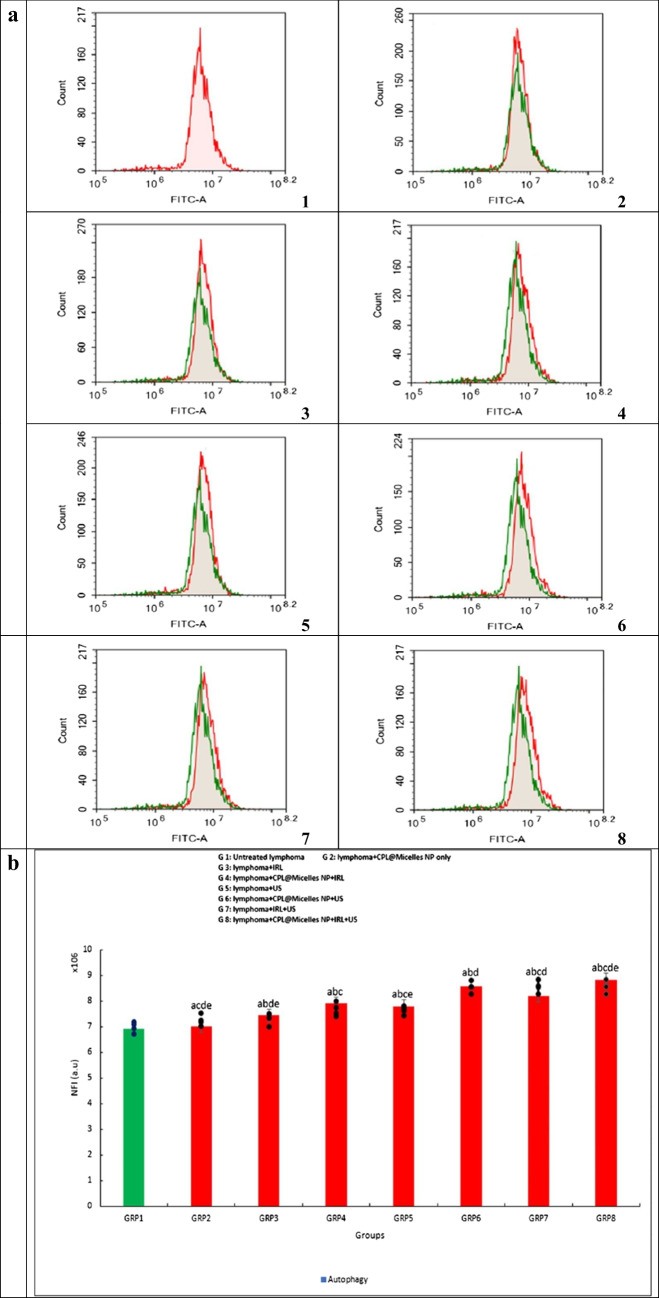
** a** The impact of various treatment approaches on the viability of prostate cancer (U-937) cells; In all in vitro research groups, cells were subjected to several treatment modalities including serial dilution of CPL-Micelles NP for 24 h, a. microscopic examinations, and b. dosage response curves. The WST-1 test was applied to assess cell viability. Viability of cells (%): F(p) = 4.718 (< 0.001*). The data ( n  = 3) are shown as mean ± SD. a,b,c,d,e Significant with (untreated prsostate cancer group, non activated CPL-Micelles NP treated group, laser subjected group, ultrasound subjected group, laser + ultrasound group). **b** The impact of various treatment approaches on the distribution of lymphoma (U-937) cell cycles throughout all in vitro research groups; 1. For a 24-h period, cells were subjected to various treatment modalities; 2. DNA cytometry analysis was applied to assess the cell cycle distribution, and the proportion of total events for each phase of the cell was plotted; SubG1, G0/G1, S, G2/M (%):F(p) = 637.920 (< 0.001*), 432.574 (< 0.001*), 78.846 (< 0.001*), 38.112 (< 0.001*).The data ( n  = 3) are shown as mean ± SD. a,b,c,d,e Significant with (untreated lymphoma group, non activated CPL-Micelles NP treated group, laser subjected group, ultrasound subjected group, laser + ultrasound group). **c** The impact of various treatment approaches on necrosis and apoptosis of lymphoma (U-937) across all in vitro research groups; 1. For 24 h, cells were subjected to various treatment modalities; 2. Annexin V-FITC/PI was applied to stain the cells, and various cell populations were plotted as a proportion of the total events. Early apoptosis, late apoptosis, early and late apoptosis, total cell death, necrosis: F(p) = 6.592 (< 0.001*), 900.167 (< 0.001*), 1.044E3 (< 0.001*), 43.717 (< 0.001*), 1.166E3 (< 0.001*). The data ( n  = 3) are shown as mean ± SD. a,b,c,d,e Significant with (untreated lymphoma group, non activated CPL-Micelles NP treated group, laser subjected group, ultrasound subjected group, laser + ultrasound group). **d** The impact of various treatment approaches on the autophagy of lymphoma (U-937) across all in vitro research groups; 1. After 24 h of exposure to various treatment modalities, cells were labeled using Cyto-ID autophagosome tracker. 2. Plotting of net fluorescent intensity (NFI; red color) was done in comparison to the control group's basal fluorescence (green color). Autophagy (%): F(p) = 19.124 (< 0.001*). The data ( n  = 3) are shown as mean ± SD. a,b,c,d,e Significant with (untreated lymphoma group, non activated CPL-Micelles NP treated group, laser subjected group, ultrasound subjected group, laser + ultrasound group)

Using the WST-1 viability test, the cytotoxic effect of various activation methods in presence and absence of CPL-Micelles was examined in lymphoma-U-937 cells following 24-h treatments. Treatment of the Lymphoma- U-937 cell line with varied CPL-Micelles dosages and activation modes resulted in an increase in floating cells and a modification of cellular shape. Furthermore, PDT, SDT, and SPDT with and without CPL-Micelles were investigated using the WST-1 cytotoxicity test. The results manifested that CPL-Micelles inhibited lymphoma-U-937 cell proliferation in a dose-dependent way. The lymphoma-U-937 cell line was also marginally impacted by treatment with CPL-Micelles without activation, according to the results of the current study's cytotoxicity investigation. This was followed by treatment of the lymphoma-U-937 cell line with laser and ultrasound without CPL-Micelles. The addition of CPL-Micelles enhances the cytotoxic efficacy of lymphoma-U-937 cells as well as growth inhibition due to PDT (laser) and SDT (ultrasound). The obtained results illustrated that US is more effective and cytotoxic than IRL against the lymphoma U-937 cell line, both with and without the CPL-Micelles. US was selected for IRL integration. The combined therapy strategy (SPDT) with CPL-Micelles is the most cytotoxic effective when used against the lymphoma- U-937 cell line, as compared to using IRL or US alone. The concentration at which viable cells are 50% inhibited by CPL-Micelles (IC50) was found by plotting cell viability vs. concentration; this value is shown in μg/ml in Fig. (3a).

### Effects of PDT, SDT and SPDT on cell cycle distribution when CPL-Micelles is present or absent during lymphoma U-937 treatment

Treatment of lymphoma- U-937 cells with CPL-Micelles at an IC50 equivalent concentration has been shown to increase the population of cell at the sub G1, S and G2/M phases. Treatment with CPL-Micelles without activation had a minor implication on the lymphoma-U-937 cell line. It increased the population of cell at the S phase, G2/M phase and leads to cell death, which was displayed as an increase in the Sub G1 phase. Conversely, it emerged a decrease in the G1 phase, and this was followed by the lymphoma- U-937 cell line irradiated with ultrasound and laser without CPL-Micelles. The presence of CPL-Micelles improved the effectiveness of laser (PDT) and ultrasound (SDT) induced growth inhibition, arrest, and distribution of lymphoma-U-937 cell line. It also showed an elevation in population of cell at the S phase, G2/M phase triggering cell death, demonstrated as an elevation in Sub G1 phase, and this in turn lead to decrease in G1phase. The resulted data illustrated that US is more effective than IRL against the lymphoma-U-937 cell line, both with and without the CPL-Micelles. US was selected for IRL integration. The combined therapeutic method (SPDT) in the CPL-Micelles presence is more efficient than using IRL or US alone when treating the lymphoma-U-937 cell line. This cell line showed an elevation in population of cell at the S phase, G2/M phase, as well as an elevation in the Sub G1 phase, which in turn, lead to a decrease in the G1 phase Fig. (3b).

### Mechanisms of necrosis and apoptosis cell death in PDT, SDT and SPDT treated Lymphoma U-937 in the presence and absence of (CPL-Micelles)

The reduction in cell viability may have been caused by an apoptotic response to CPL-Micelles, according to the WST-1 test and microscopic analysis. To be able to get additional understanding of the mechanism underlying cell death (apoptosis versus necrosis), lymphoma-U-937 cells were exposed to activation several modalities for a whole day, both with and without CPL-Micelles. The treated cells' flowcytometric histogram manifested a shift in population; surviving cells were in the lower left quadrant, followed by early apoptosis in the lower right, late apoptosis in the upper right quadrant, and cell necrosis in the upper left. An increase in annexin V positivity was also seen in the treated cells. The proportion of lymphoma-U-937 cells that underwent either CPL-Micelles without activation or laser and ultrasound without CPL-Micelles treatment showed a significant elevation in early and late apoptotic cell percentages. Adding CPL-Micelles to lymphoma-U-937 cells irradiated with PDT (laser) and SDT (ultrasound) enhances the percentage of exhibiting early and late apoptosis cells. The obtained data demonstrated that, in both cases—with and without the CPL-Micelles—US is more efficient than IRL at increasing the percentage of exhibiting early and late apoptosis lymphoma-U-937 cells. US was selected for IRL integration. Compared to employing IRL or US alone, the combined SPDT therapeutic technique in the CPL-Micelles presence is the most efficient way to raise the fraction of lymphoma-U-937 cells with early and late apoptosis. Comparable results were seen for the fraction of necrotic cells in lymphoma-U-937 cells irradiated with laser and ultrasound without CPL-Micelles, or with CPL-Micelles non activation. When CPL-Micelles is added, the fraction of lymphoma-U-937 necrotic cells when cells irradiated with PDT (laser) and SDT (ultrasound) is improved. The results obtained demonstrated that, in both cases—with and without the CPL-Micelles -US is more efficient than IRL at the necrotic lymphoma-U-937 cells percentage rising. When compared to using US or IRL alone, the SPDT combination therapy method in the CPL-Micelles presence is more efficient in increasing the percentage of lymphoma-U-937 cells with necrosis Fig. (3c).

### Mechanisms of autophagy cell death in PDT, SDT and SPDT treated lymphoma U-937 in the presence and absence of (CPL-Micelles)

The microscopic and WST-1 assay analysis demonstrated that the reason of the cell viability loss may be program cell death rather than autophagic apoptosis in response to CPL-Micelles. To obtain further understanding of the cell death process, (autophagy), lymphoma-U-937 cells were activated in both the presence and absence of CPL-Micelles using various modalities for a whole day. When AO, a fluorophore that builds up at high concentrations in autolysosomes acidic vesicular organelles (AVO), dimerizes and induces a green metachromatic shift to red, the treated cells produced more red and less green, which could be evaluated to investigate autophagy. The percentage of lymphoma-U-937 cells undergoing autophagy was much higher in those treated with CPL-Micelles without activation or with laser and ultrasound without CPL-Micelles. When CPL-Micelles is present, Lymphoma-U-937 cells treated with both SDT (ultrasound) and PDT (laser) treatment have a higher percentage of autophagous cells. The obtained data manifested that, both with and without the CPL-Micelles, US is more efficient than IRL at increasing the proportion of lymphoma-U-937 cells undergoing autophagy. US was selected for IRL integration. When compared to utilizing IRL or US alone, the SPDT combination therapeutic method in the presence of CPL-Micelles is more effective in increasing the percentage of lymphoma-U-937 cells undergoing autophagy Fig. (3d).

### Oxidative stress and PDT, SDT, SPDT in in vivo Lymphoma models in the presence and absence of (CPL-Micelles)

**Fig. 4:**
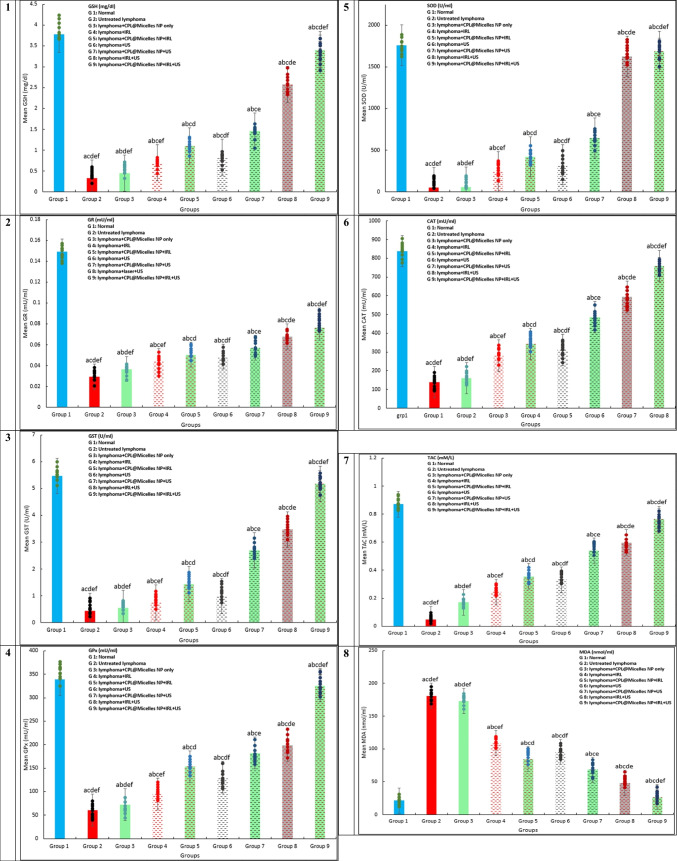

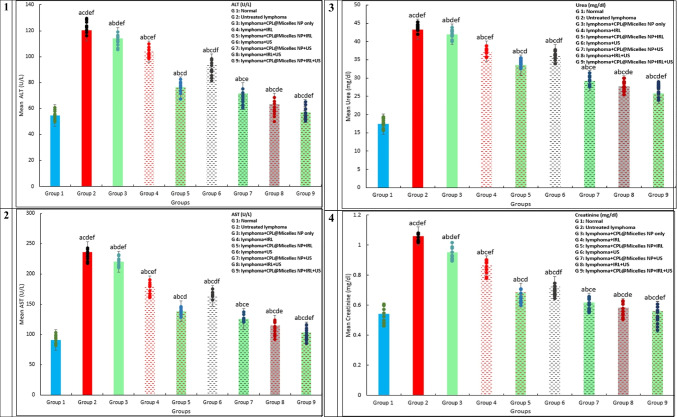

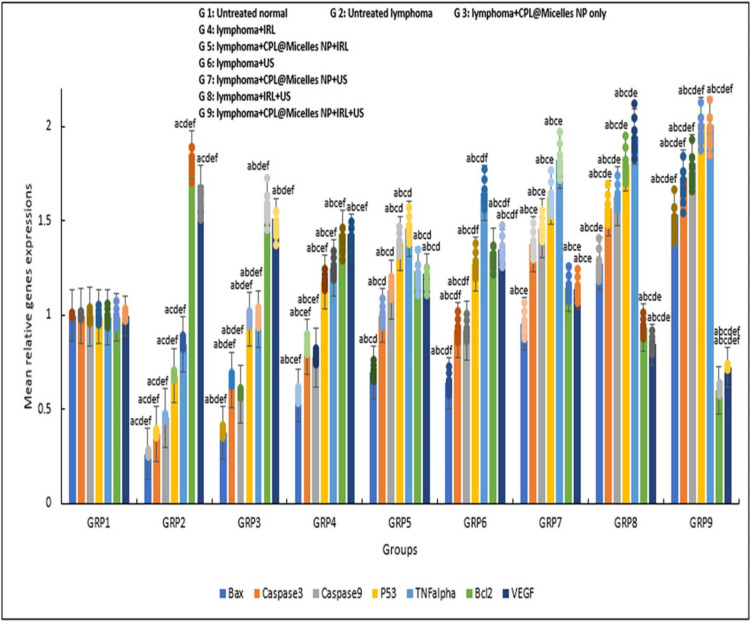

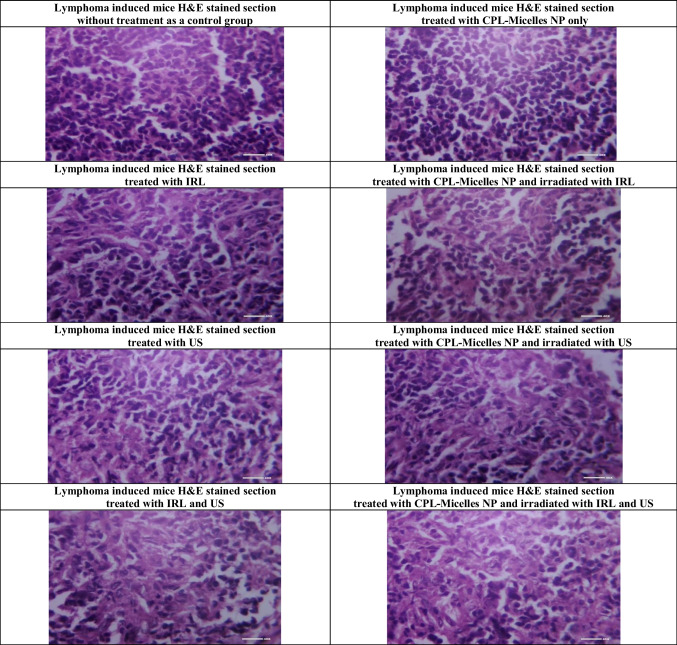
**a** The impact of various treatment approaches on MDA, antioxidant activities, and capacity across all research groups; F represents the ANOVA test value. 1–8. GR (mU/ml), GSH (mg/dl), GST (U/ml), GPx (mU/ml), SOD (U/ml), CAT (mU/ml), TAC (mM/L), MDA (nmol/ml): F(p) = 105.046 (< 0.001*), 408.814 (< 0.001*), 1.217E3 (< 0.001*), 2.703E4 (< 0.001*), 3.450E6 (< 0.001*), 1.721E5 (< 0.001*), 3.328E3 (< 0.001*), 9.669E4 (< 0.001*). The data ( n  = 10 each group) are shown as mean ± SD. a,b,c,d,e Significant with (untreated lymphoma group, non activated CPL-Micelles NP treated group, laser subjected group, ultrasound subjected group, laser + ultrasound group). **b** The impact of various treatment approaches on hepatic and renal biomarkers across all research groups; F represents the ANOVA test value. ALT (U/l), AST (U/l), urea (mg/dl), creatinine (mg/dl): F(p) = 5.184E3 (< 0.001*), 1.778E5 (< 0.001*), 3.853E3 (< 0.001*), 316.670 (< 0.001*). The data ( n  = 10 each group) are shown as mean ± SD. a,b,c,d,e Significant with (untreated lymphoma group, non activated CPL-Micelles NP treated group, laser subjected group, ultrasound subjected group, laser + ultrasound group). **c** The impact of various treatment modalities on the gene relative expressions of TNF alpha, Bax, Caspase (9,3), p53, VEGF, and Bcl-2 as measured by qRT-PCR in each research group; F represents the ANOVA test result. p53, Bax, Caspase 9, Caspase 3, TNFalpha, VEGF, Bcl-2: F(p) = 576.752 (< 0.001*), 650.868 (< 0.001*), 895.850 (< 0.001*), 749.625 (< 0.001*), 456.606 (< 0.001*), 173.303 (< 0.001*), 217.722 (< 0.001*). The data ( n  = 10 each group) are shown as mean ± SD. a,b,c,d,e Significant with (untreated lymphoma group, non activated CPL-Micelles NP treated group, laser subjected group, ultrasound subjected group, laser + ultrasound group). **d** The H&E-stained segment of lymphoma tissue in all research groups, which illustrates the impact of various treatment regimens at the cellular level (Magnification × 40); 1. Normal lymphoma untreated group, 2. DMBA induced lymphoma group untreated, 3. DMBA induced lymphoma group subjected to CPL-Micelles without activation, 4. DMBA induced lymphoma group subjected to laser only, 5. DMBA induced lymphoma group subjected to laser in presence of CPL-Micelles, 6. DMBA induced lymphoma group subjected to ultrasound only, 7. DMBA induced lymphoma group subjected to ultrasound in presence of CPL-Micelles, 8. DMBA induced lymphoma group subjected to combined modalities laser/ultrasound only, 9. DMBA induced lymphoma group subjected to combined modalities laser/ultrasound in presence of CPL-Micelles

The impact of CPL-Micelles; PDT, SDT, and SPDT on the MDA parameter for oxidative stress and lipid peroxidation in the mice of each experimental group are shown in Fig. (4a). The MDA in the sera of the mice group treated with CPL-Micelles alone and non-irradiated revealed very slight modifications as compared to the untreated DMBA-lymphoma-induced control mouse group. Regarding to control healthy normal mice, the untreated DMBA-lymphoma-induced control animals exhibited noticeably higher levels of this parameter. Additionally, as regard to the normal control mice, all DMBA-lymphoma-induced mice irradiated with ultrasound, laser, or ultrasound and laser alone combined groups demonstrated a significant increase in MDA concentrations. When CPL-Micelles was administered, MDA levels were significantly decline in the IRL, US, and combination of IRL and US activated groups than in the DMBA-lymphoma-induced control animals; however, this impact did not achieve the normal level of control mice.

### **Antioxidant system and PDT, SDT, SPDT in vivo lymphoma models in the presence and absence of (CPL-Micelles)** 

Fig. (4a) shows how the antioxidant markers (SOD, Catalase, TAC, GPx, GST, and GR) were impacted in each of the mouse research groups by CPL-Micelles, PDT, SDT, and SPDT. The CPL-Micelles alone without activation was contrasted with the DMBA-lymphoma-induced control group. Changes in the activity of GPx, GST, GR, catalase, and SOD and TAC level, were only slightly significant. SOD, Catalase, GPx, GST, GR, and TAC levels were significantly lower in the untreated control DMBA-lymphoma-induced animals than in the normal control mice. In addition, both DMBA-lymphoma-induced animal groups treated with ultrasound, laser, or ultrasound and laser combination alone manifested a significant decline in TAC levels and in the SOD, Catalase, GPx, GST, and GR activities regarding to the mice healthy control group. Compared to the untreated DMBA-lymphoma-induced control mice, activated CPL-Micelles in the IRL, US, and combination of IRL and US groups demonstrated a considerable elevation in the SOD, Catalase, GPx, GST, and GR, activities and TAC level while not approaching the level of normal control group.

### The liver functions and PDT, SDT, SPDT in vivo lymphoma models in the presence and absence of (CPL-Micelles)

The liver function tests data for each study group are displayed in Fig. (4b). The mice treated with CPL-Micelles alone and non irradiation demonstrated only slightly different changes in their levels of AST and ALT from the untreated DMBA-lymphoma-induced control group, despite the fact that the sera levels of this group were notably increased than those of the healthy normal control mice. Moreover, ALT and AST levels were notably higher in all DMBA-lymphoma-induced mice irradiated with ultrasound, laser, or ultrasound and laser combination alone groups than in the control normal group. Furthermore, when CPL-Micelles was administered to the IRL, US, and combination of IRL and US activated groups, the level of ALT and AST was drastically lowered in contrast to the untreated DMBA-lymphoma-induced control mice; however, the levels of normal control group were not attained.

### The kidney functions and PDT, SDT, SPDT in vivo lymphoma models in the presence and absence of (CPL-Micelles)

The data of the renal function tests conducted on each of the research groups are displayed in Fig. (4b). The levels of urea or creatinine in CPL-Micelles treated mice with solely and non irradiated demonstrated only marginally considerable changes regarding to the DMBA-lymphoma-induced mice untreated control; however, these parameter levels in the DMBA-lymphoma-induced mice untreated control were notably higher regarding to the control healthy normal mice. Moreover, creatinine and urea levels were statistically significantly higher in all DMBA-lymphoma-induced mice irradiated with ultrasound, laser, or ultrasound and laser combination alone groups than in the control normal group. Additionally, the treatment of CPL-Micelles in the IRL, US, and combination of IRL and US activated groups notably decline the levels of creatinine and urea with regard to the DMBA-lymphoma-induced control untreated mice; however, the levels of normal control group were not attained.

### Anticancer, antiproliferative, and antiangiogenic effects and PDT, SDT, SPDT in vivo lymphoma models in the presence and absence of (CPL-Micelles)

In all study groups, Fig. (4c) illustrates how CPL-Micelles affects the p53, Bax, TNF alpha, Caspase (3, 9), VEGF, and Bcl-2 relative genes expressions. The p53, Bax, TNF alpha, Caspase (3, 9), VEGF, and Bcl-2 expression altered very marginally when CPL-Micelles was given to mice solely non activated compared to the untreated control DMBA-lymphoma-induced group. On the other hand, the levels of p53, Bax, TNF alpha, and caspase (3, 9) in the untreated DMBA-lymphoma-induced control group were considerably lower than those of healthy normal control mice, while those of VEGF and Bcl-2 were significantly higher. In addition, all DMBA-lymphoma-induced mice irradiated with ultrasound, laser, or ultrasound and laser combination only groups demonstrated significantly declined levels of p53, TNF alpha, Bax, and caspase (3, 9) expressions and notably increased levels of VEGF and Bcl-2 expressions when regarding to the healthy control group of mice. Regarding to the untreated DMBA-lymphoma-induced control group, the delivery of CPL-Micelles to the IRL, US, and combination of IRL and US activated groups led to significant decline in the expressions of the Bcl-2 and VEGF genes and elevation in the p53, Bax, TNF alpha, and caspase (3, 9) expressions.

### **Histopathological effect and PDT, SDT, SPDT in vivo lymphoma models in the presence and absence of (CPL-Micelles)** 

Sections of tissue stained with H&E from every mouse study group are shown in Fig. (4d), which illustrates the impact of CPL-Micelles, PDT, SDT, and SPDT on lymphoma brought on by DMBA. The histological analysis revealed that every tumor in the untreated DMBA-lymphoma-induced control group included 5% necrosis and was made up entirely of extensively cancerous cells. The DMBA histologically caused Lymphoma tissues in the CPL-micelles treated mice solely and non irradiated, exhibited only slightly significant changes as compared to the untreated control DMBA-lymphoma-induced mice. Regarding to the untreated DMBA-lymphoma-induced control mice, the injection of CPL-micelles in the ultrasound, laser, and ultrasound and laser combination activated groups showed considerably necrotic big foci regions (91–96%). Furthermore, regarding to the untreated DMBA-lymphoma-induced control group, all DMBA-lymphoma-induced mice irradiated with IRL, US, or a combination of IRL and US only showed significant areas of necrosis.

## Discussion

The WST-1 assay results are reliable in order to find out the exact number of viable cells and assessing the cytotoxicity of medications used to treat cancer. One of the primary factors contributing to cancer is the cell cycle disruption, which is the fundamental system controlling a cell's life processes. Furthermore, apoptosis, necrosis, and autophagy are the three types of cell programmed death that maintain the balance of biological processes involved in proliferation and are essential for normal development. Tumor growth and metastasis are thought to be associated with abnormal proliferation and suppressed apoptosis, as most malignancies rely on the ability of tumor cells to outnumber apoptosis in order to live. [36–39]

The results of this study's cytotoxicity (WST-1 assay) as well as cell cycle analysis, necrosis, apoptosis, and autophagy (flowcytometry) revealed that the lymphoma-U-937 cell line was mostly unaffected by treatment with non activated CPL-Micelles after a 24-h incubation period. Without CPL-Micelles, the application of laser and ultrasound had no discernible effect on the lymphoma-U-937 cell line. When CPL-Micelles is present, both the SDT (ultrasound) and PDT (laser) are more effective. The acquired results showed that when treating the lymphoma-U-937 cell line, the therapeutic (SPDT) combined approach is more efficient than either IRL or US alone (decline of cell viability in a dose-dependent manner, slowed down of progression of cell cycle in S and G2/M phase, and cell death was provoked as revealed by rise in Early-G cell population, an elevation in necrosis, early and late apoptosis, and an increase in autophagic cell death). Our results are in line with earlier in vitro studies against cancer of cardamom [40–43], pistacia [44–47] and laurel [48–51]. demonstrating the inhibitory effect (cell cycle arrest; down of progression of cell cycle in in S phase and G2/M phase), as well as destroying tumor cells (decline of cell viability in a dose-dependent manner, necrosis, apoptosis, autophagy) was maximum in presence of CPL-Micelles as sensitizer combined with both laser (-photo) and ultrasound (-sono) activation (SPDT) followed by CPL-Micelles ultrasound only (SDT activation and laser activation only (PDT) and that SPDT superior to SDT and PDT.

A well-known immunosuppressant and carcinogen, DMBA is employed in rodent models of cancer research. The incomplete combustion of complex hydrocarbons releases DMBA, a polycyclic aromatic hydrocarbon, into the atmosphere. It is an indirect carcinogen that is broken down by cytochrome P 4501B1 into harmful compounds (DMBA-3,4-dihydrodiol-1,2-epoxide (DMBA-DE)), and ROS. The harmful metabolites of DMBA attach to DNA adenine residues (DNA adducts) and cause damage, and these ROS cause lipid peroxidation and exhaustion the cell's antioxidant defense mechanisms either directly or indirectly by functioning as second messengers for the initial free radicals. It's possible that the in situ production of hydroxyl radicals and other ROS caused the DNA breaks. Furthermore, it is thought that reactive material is created when oxygen radicals break deoxyribose or DNA. Additional evidence of increased DNA damage from restraint stress is provided by the dose-dependent rise in MDA formation a consequence of lipid peroxidation production in both liver tissues and the bloodstream following DMBA exposure. Previous research indicates that the DMBA generated cancer is dose dependent; lymphoma, lymphoblastic, myelogenous, and thymic leukemias as well as widespread hepatic leukemia of erythroblastic stem cells and mammary tumors. [34, 35, 52–56]

The present study provides evidence of the oxidants that are driving the advancement of DMBA-induced lymphoma. The untreated control DMBA-lymphoma-induced mice had noticeably increased MDA levels. All treated CPL-Micelles groups (non-activated, laser-, ultrasound-, laser and ultrasound combination-activated mice) had significantly lower MDA levels than the untreated control DMBA-lymphoma-induced animals. The antioxidant system is further disrupted by significant decreases in the GPx, GST, GR, Catalase, SOD activity and TAC backup. The DMBA-lymphoma-induced control mice that were not given any treatment had noticeably reduced antioxidant levels. Compared to untreated DMBA-lymphoma-induced control mice, antioxidant levels were significantly greater in all treated CPL-Micelles groups (laser-, ultrasound-, laser and ultrasound combined- activated mice). Prior research has demonstrated that induction of DMBA-lymphoma causes a noteworthy drop in TAC, GPx, GST, GR, Catalase, and SOD activity, as well as a notable increase in MDA. [56–63] It was found that, in comparison to mice from a normal, healthy control group, mice with lymphoma produced by DMBA had decreased capacity in free radicals scavenging and were more susceptible to lipid peroxidation. A surplus and accumulation of reactive oxygen species (ROS) in addition an increase in the oxidation polyunsaturated fatty acid phospholipid bilayers of cell, which in turn boosts MDA levels, are the results of DMBA-lymphoma-induction, as was previously noted. One crucial stage in the emergence of oxidative stress and cancer is the degradation of enzymatic and non-enzymatic antioxidants. Because DMBA-lymphoma-induced ROS and their metabolites required the detoxification of these antioxidants, both non-enzymatic and enzymatic, their constant depletion explained why they were deficient. The catalytic activity of enzymatic antioxidants could be compromised by the build-up of ROS. [56, 64–69] Our results showed how the non-activated portion of CPL-Micelles ability to scavenge free radical production, hence lowering MDA levels, supports its anti-lipid peroxidative effect. Further evidence of the efficacy of CPL-Micelles action as SPS and its activation by PDT, SDT, and SPDT, that eliminated DMBA-lymphoma cancerous cells-the main source of reactive oxygen species-and led to an increase in antioxidant enzyme activities, a restoration of the antioxidant system, and a delay in the progression of the cancerous state to a nearly normal state. Our findings concurred with earlier research. [58, 70–74] manifesting the SPDT biochemical impact (decline of MDA level and restoring enzymatic and non enzymatic antioxidants to near normal levels; destroying DMBA-induced lymphoma cells the main source of ROS, as well as blocking the antioxidants exhausting by ROS evolved from DMBA-induced lymphoma cells) was maximum in presence of CPL-Micelles as sensitizer combined with both laser (-photo) and ultrasound (-sono) activation followed by CPL-Micelles ultrasound only activation and laser activation only and that SPDT superior to SDT and PDT.

There was a significant elevation in ALT and AST in the DMBA-Lymphoma-induced groups in our study, indicating liver cell injury. Increased serum concentrations of a number of liver enzymes are linked to those enzymes cellular leaking into the bloodstream, a sign of hepatocyte membrane integrity being compromised. [56, 61–64] The present study's results are in line with earlier research, which indicates that DMBA-induced animals had lower hepatic function with regard to normal control mice. This is likely because of the disruption of metabolism caused by DMBA, which also leads to organ failure. [56, 67–70] The results of the present investigation showed that CPL-Micelles protected the liver by lowering ALT and AST levels. Moreover, this reinforces CPL-Micelles preventive effectiveness in preventing hepato-dysfunction in mice that is caused by DMBA-lymphoma. In this study, DMBA also clearly elevated the levels of creatinine and urea in the DMBA-induced groups, indicating kidney injury. Renal failure in DMBA-lymphoma-induced mice has been shown to be caused by cardiac and hepatic injury, leading to increased tubular and glomerular congestion. The entire capillary and tubule system experiences a rise in renal interstitial pressure as a result of this congestion. The results of this study demonstrate that DMBA-lymphoma-induced animals exhibited reduced renal function in comparison to control normal mice, which is consistent with earlier findings. [9, 10, 56, 73–77] The current investigation found that CPL-Micelles induced kidney protection by lowering serum levels of urea and creatinine. Additionally, study demonstrates that CPL-Micelles can prevent renal impairment in mice caused by DMBA-lymphoma. Our research showed that the non-activated portion of CPL-Micelles protects the liver and kidneys by scavenging free radicals produced by DMBA-lymphoma induction. Activated CPL-Micelles also effectively removes DMBA, the main source of ROS, leading to the restoration of liver and kidney function as well as a shift from a cancerous to a nearly normal state. This ameliorating effect was maximum in presence of CPL-Micelles as sensitizer combined with both laser (-photo) and ultrasound (-sono) activation followed by CPL-Micelles ultrasound only activation and laser activation only and that SPDT superior to SDT and PDT.

In the current investigation, we molecularly examined the p53, TNF alpha, Bax, VEGF, Caspase (3,9), and Bcl-2 expressions as markers of DMBA-induced lymphoma therapy and inhibition of angiogenesis,. The findings show a strong positive link between the therapy and gene expressions in the presence of CPL-Micelles using various modalities, but a notably negative correlation between the gene expressions and DMBA-lymphoma-induction. The p53, TNF alpha, Bax, and caspase 3, 9 expressions was considerably higher in the groups receiving sono-photo-dynamic treatment with CPL-Micelles than in the PDT or SDT with CPL-Micelles, IRL or US alone without CPL-Micelles, and the untreated DMBA-lymphoma-induced mice. Conversely, expressions of VEGF and Bcl-2 correlated positively in the DMBA-lymphoma-induced mice that were left untreated, but expressions of VEGF and Bcl-2 correlated negatively with various modalities when CPL-Micelles was present. The Bcl-2 and VEGF genes expressions were significantly decline in mice subjected to SPDT therapy with (CPL-Micelles) as regard to mice irradiated with PDT or SDT solely with (CPL-Micelles), and then IRL or US solely without (CPL-Micelles). When DMBA-lymphoma stimulated mice, the untreated group exhibited the highest expression level. The outcomes of our investigation, which align with prior research, demonstrate the validity of p53, TNF alpha, Bax, VEGF, Caspase (3,9), and Bcl-2 genes expressions as trustworthy markers of treatment-relevant malignancy. [9, 10, 15, 78–80] illustrating the SPDT molecular level implications (down regulation of VEGF and Bcl-2 levels; restricting the proliferation and limiting the angiogenesis capabilities of DMBA-induced lymphoma cells) as well as (up regulation of pro-apoptotic genes p53, caspase 3, 9, Bax, and TNF alpha; directing DMBA-induced lymphoma cells to be destroyed by either programmed cell death or necrosis and alerting the immune system) was maximum in presence of CPL-Micelles as sensitizer combined with both laser (-photo) and ultrasound (-sono) activation followed by CPL-Micelles ultrasound only activation and laser activation only and that SPDT superior to SDT and PDT.

According to our current research, CPL-Micelles may be employed as a photo- and ultrasound sensitizer to treat DMBA-induced in vivo lymphoma. The CPL-Micelles, which may be induced by chemical activation processes mediated by laser photons or ultrasound, dramatically suppresses tumor growth and results in cell death. One explanation for the sono-photo-chemical/physical mechanisms that underlie the biological effects of SPDT could be that an electron is propelled into a higher energy orbital by laser photon and ultrasound, which excite a ground state SPS (CPL-Micelles) molecule in a singlet state. At lower energies, the excited triplet state SPS has a relatively lengthy half-life (~ microseconds) before reverts from the excited state to the ground state by internal or fluorescence conversion (~ nanoseconds). Alternatively, the excited electron's spin inverts. In a spin-forbidden transfer, phosphorescence returns to the ground singlet state and loses its excitation energy. The two possible outcomes of triplet oxygen excitation are: either it transfers an electron to superoxide anions, producing a range of ROS that can cause significant damage to biological systems, or it transfers its energy to triplet molecular oxygen, producing reactive biologically excited singlet state oxygen. Innate and adaptive immune systems both mount a strong defense in response to SPDT. It also causes cell death via a number of different pathways, such as necrosis, autophagy, and apoptosis.

Finally, in the CPL-Micelles presence, anticancer effects can be achieved through the application of laser and ultrasound. It is proposed that a highly successful anticancer treatment is laser photon-dynamic therapy combined with sono-dynamic therapy. Our findings indicate that CPL-Micelles have a promising potential as a novel sensitizer and efficient drug delivery method for lymphoma sono-photodynamic SPDT treatment.

## Conclusion

The current study delivered substantial outcomes involving the application pulsed cavitation of conjugated micelles nanoparticles of cardamom, pistacia and laurel (CPL-Micelles) as a sensitizer delivery system for lymphoma sono-photodynamic therapy (SPDT) in vitro (using the U-937 cell line) and (using DMBA-induced mice) in vivo demonstrated significant results in this work, indicating promising outcomes in the treatment of cancer. In addition, CPL-Micelles many benefits, including its low systemic toxicity and excellent bioavailability, make it a feasible alternative for treating cancer. Moreover, the CPL-Micelles NP@US@IRL combination has paved a wide range of options for anticancer drugs for the effective eradication of lymphoma.

## Recommendation

The current investigation emerged the potential of conjugated micelles nanoparticles from cardamom, pistacia and laurel assisted by pulsed cavitation (CPL-Micelles) as a novel sensitizer in conjunction with sono- photodynamic therapy (SPDT), a therapeutic approach that is still needs more confirmation and further validation for the treatment of lymphoma. It is strongly advised to conduct additional research using techniques that safely apply this contemporary advanced technology and approach to people and monitor modifications in various biochemical and/or biophysical indicators.

## Supplementary Information

Below is the link to the electronic supplementary material. 

ESM1 (DOCX 6.85 MB)

## Data Availability

The datasets generated during and/or analyzed during the current study are available from the corresponding author on reasonable request.

## Abbreviations

ALT: Alanine aminotransferase
AST: Aspartate aminotransferase
CAT: Catalase
CPL-Micelles NP: Cardamom, pistacia and laurel conjugated micelles nanoparticles
DMBA: 9,10-Dimethyl-1,2-Benzanthracene
GPx: Glutathione peroxidase
GR: Glutathione reductase
GSH: Reduced glutathione
GST: Glutathione-S-transferase
MDA: Malondialdehyde
PDT: Photodynamic therapy
ROS: Reactive oxygen species
SDT: Sonodynamic therapy
SOD: Superoxide dismutase
SPDT: Sono-photo-dynamic therapy
TAC: Total antioxidant capacity
