# Supplementary material for: Pulsed cavitation ultrasound assisted delivery of cardamom, pistacia and laurel encapsulated micelles nanoparticles for sono-photodynamic lymphoma in vitro and in vivo treatment
Source: Lasers Med Sci. 2025 Mar 24;40(1):156. doi: 10.1007/s10103-025-04387-x (PMC11933185; doi:10.1007/s10103-025-04387-x)
Supplement: Supplementary file 1 — (DOCX 6.85 MB) [file 10103_2025_4387_MOESM1_ESM.docx]

**
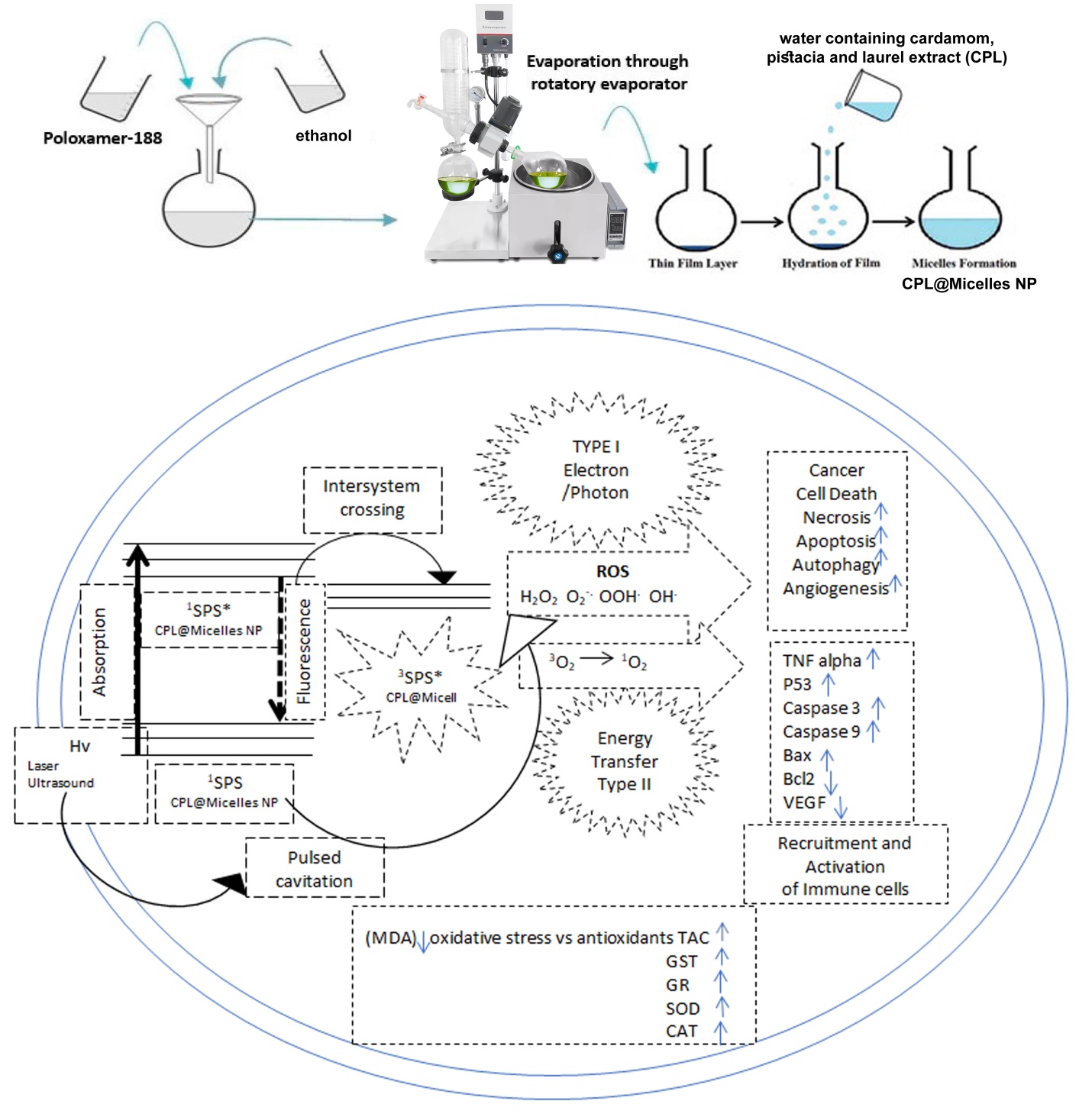
**

**Fig (1): Schematic illustration of SPDT and CPL-Micelles NP synthesis.**

| **a** | 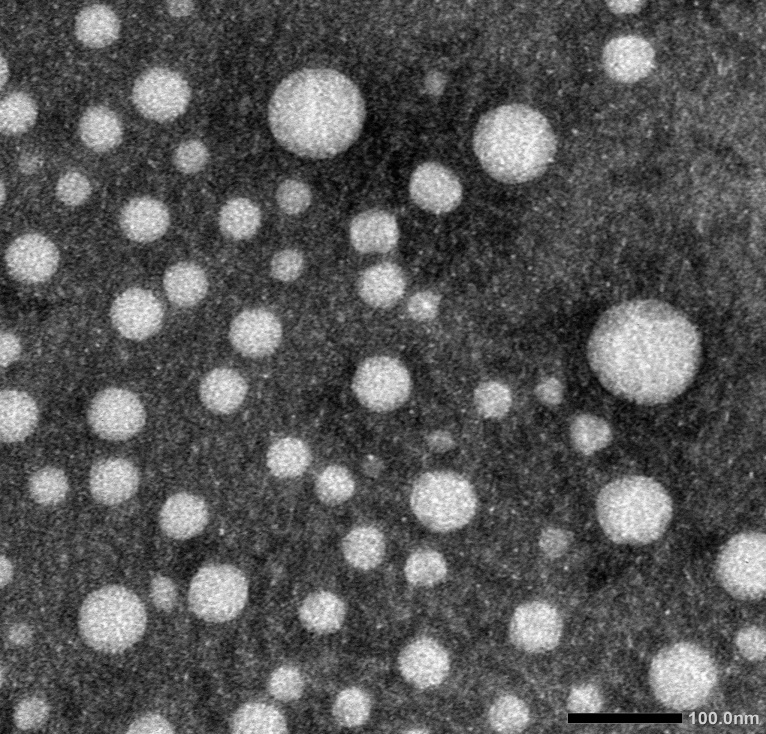 |
| --- | --- |
| **b** | 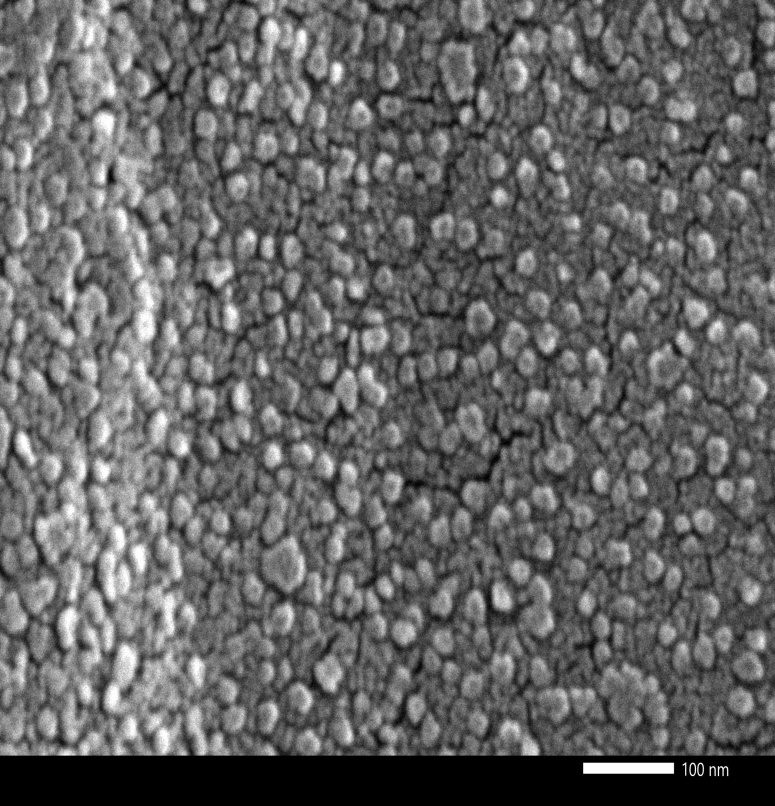 |

| **c** | 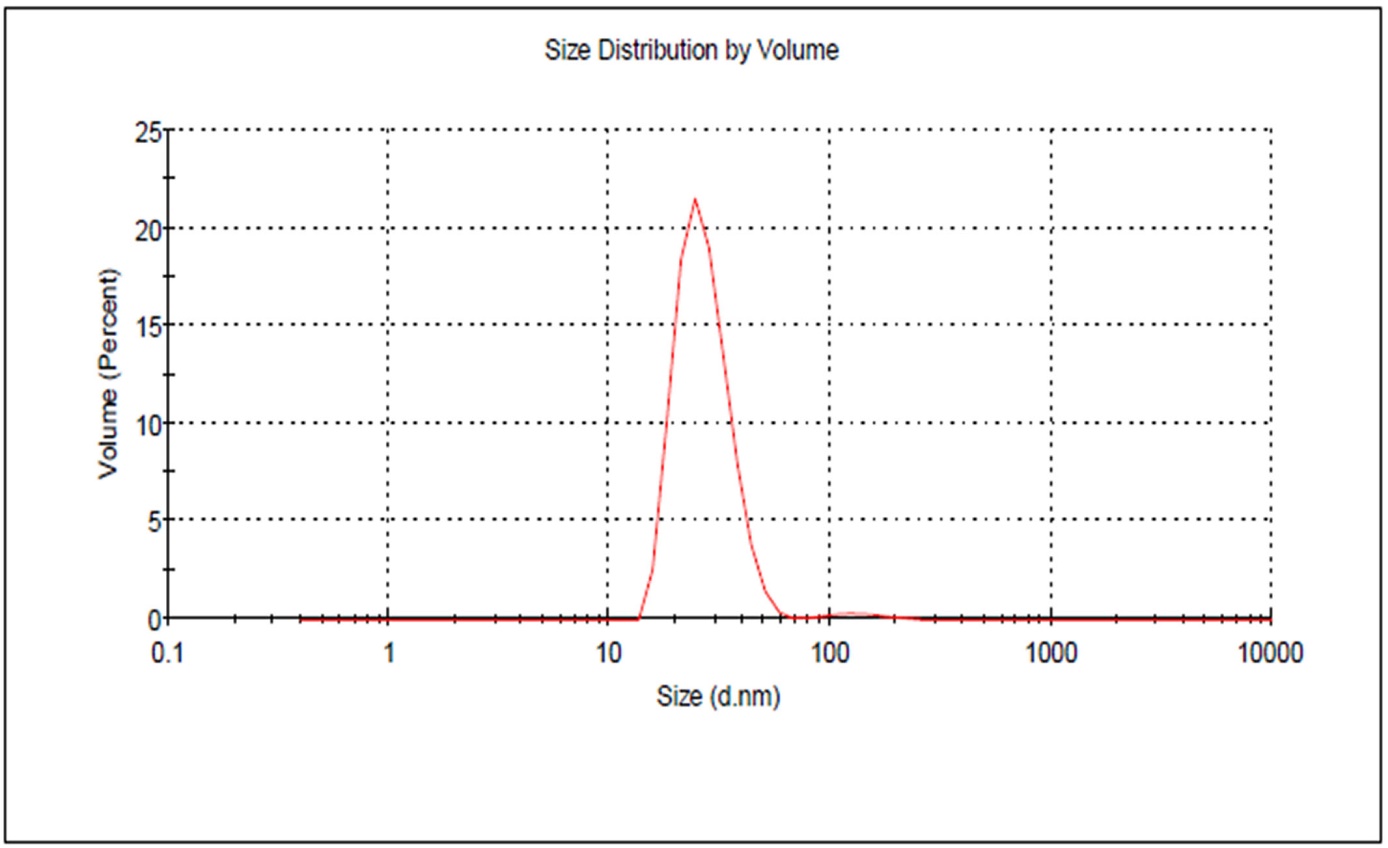 |
| --- | --- |
| **d** | 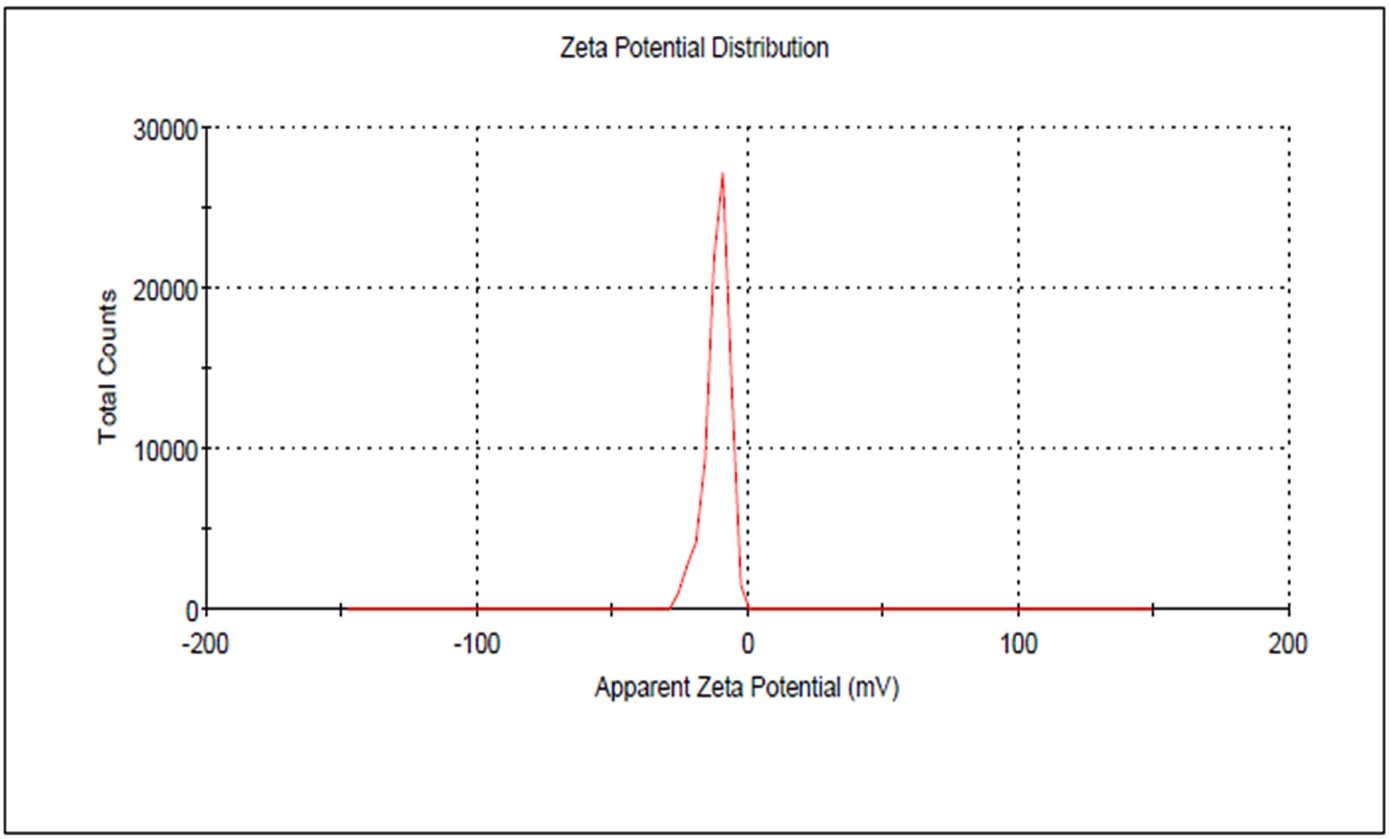 |

| **e**  **1**  **3** | 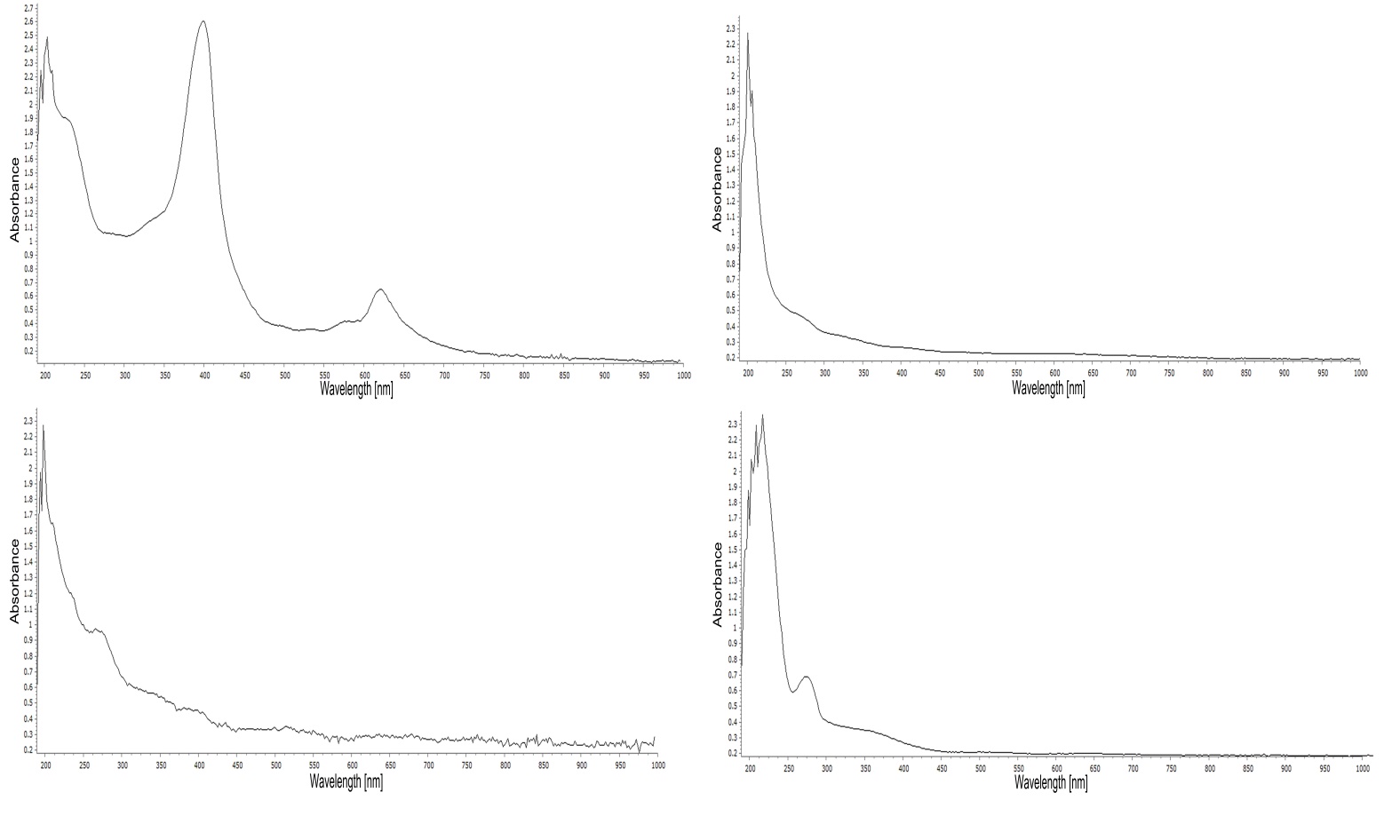 | **2**  **4** |
| --- | --- | --- |

| **f**  **1**  **3** | 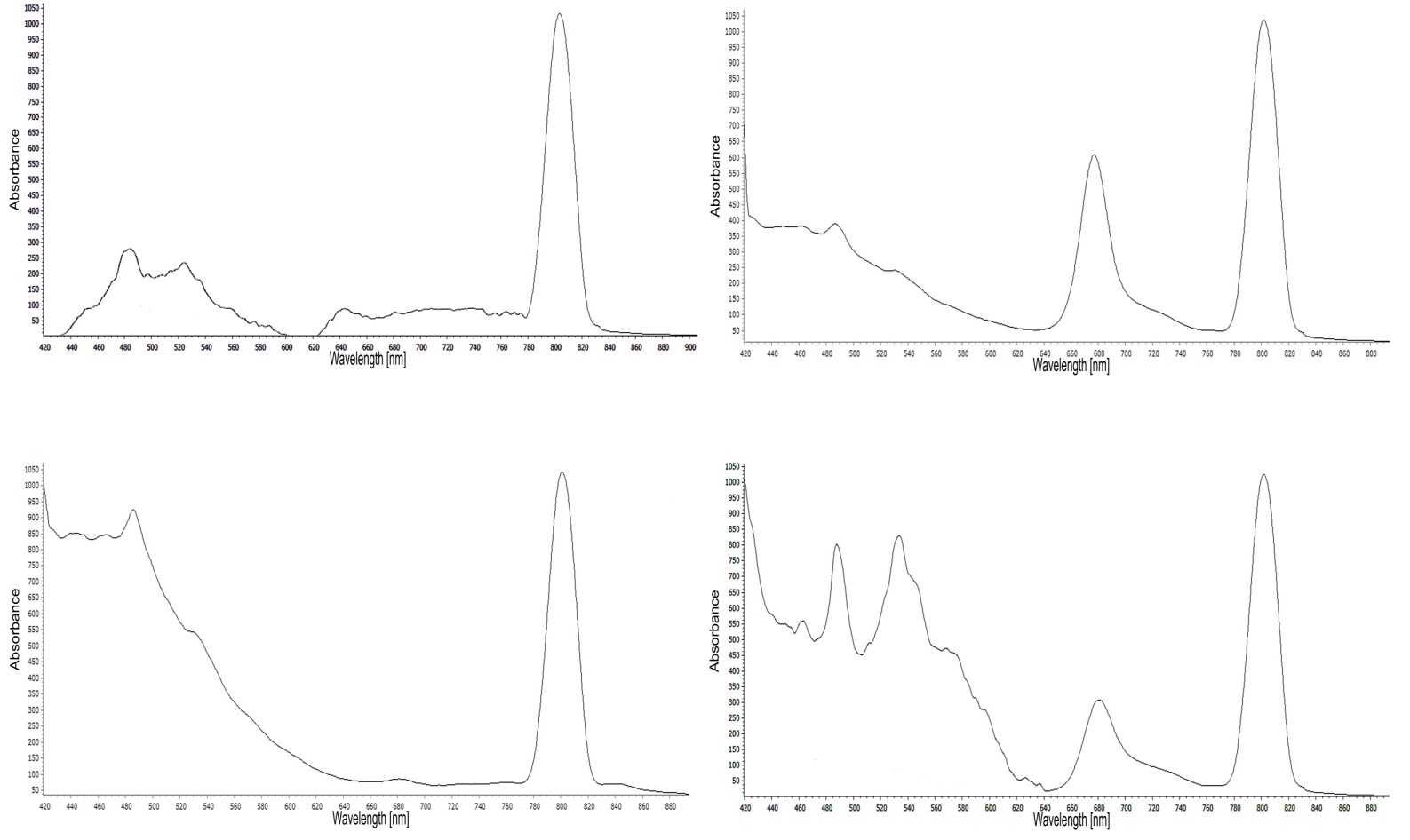 | **2**  **4** |
| --- | --- | --- |

| **g**  **1**  **2**  **3**  **4** | 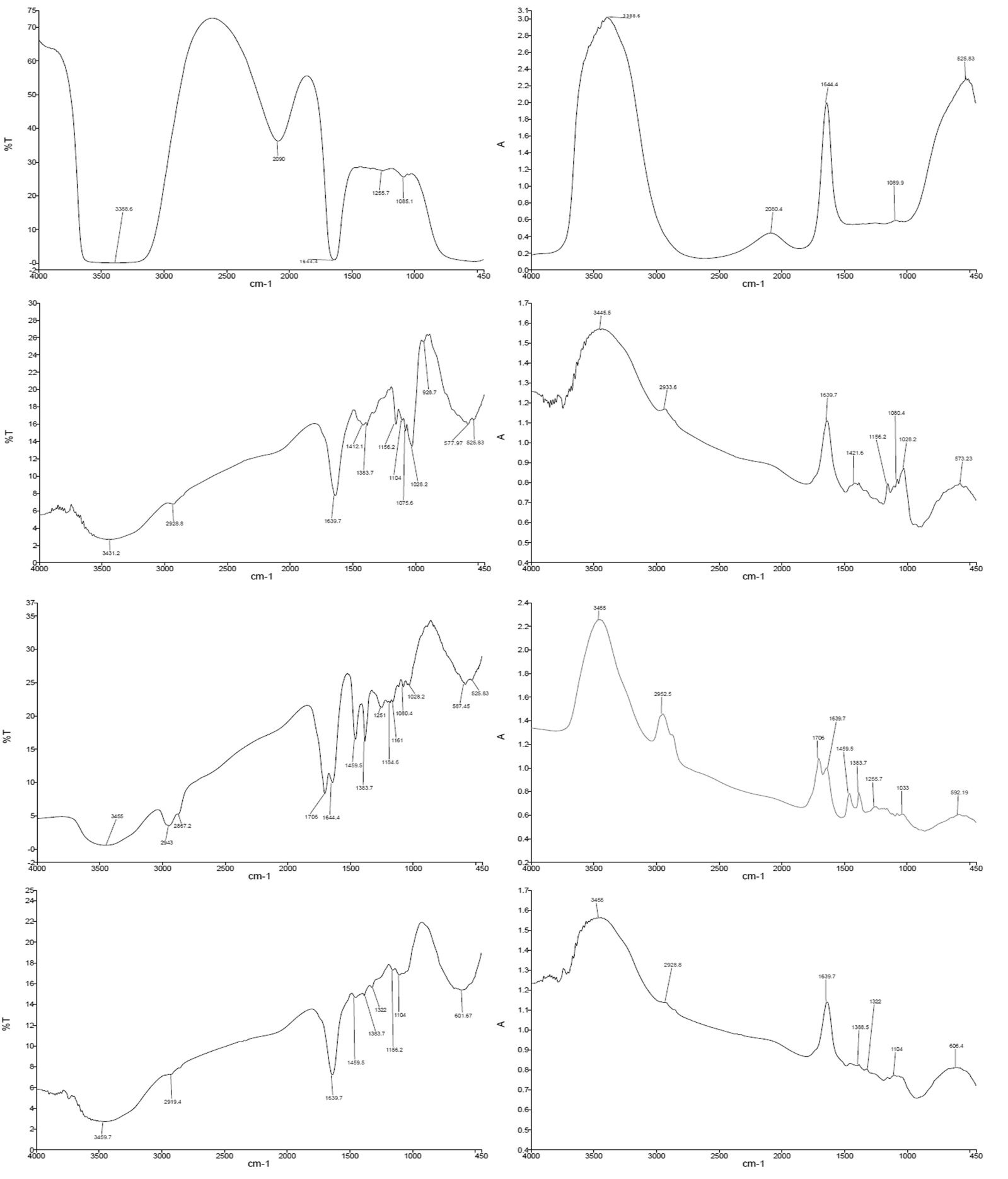 | **1**  **2**  **3**  **4** |
| --- | --- | --- |

| **h**  **1**  **3** | 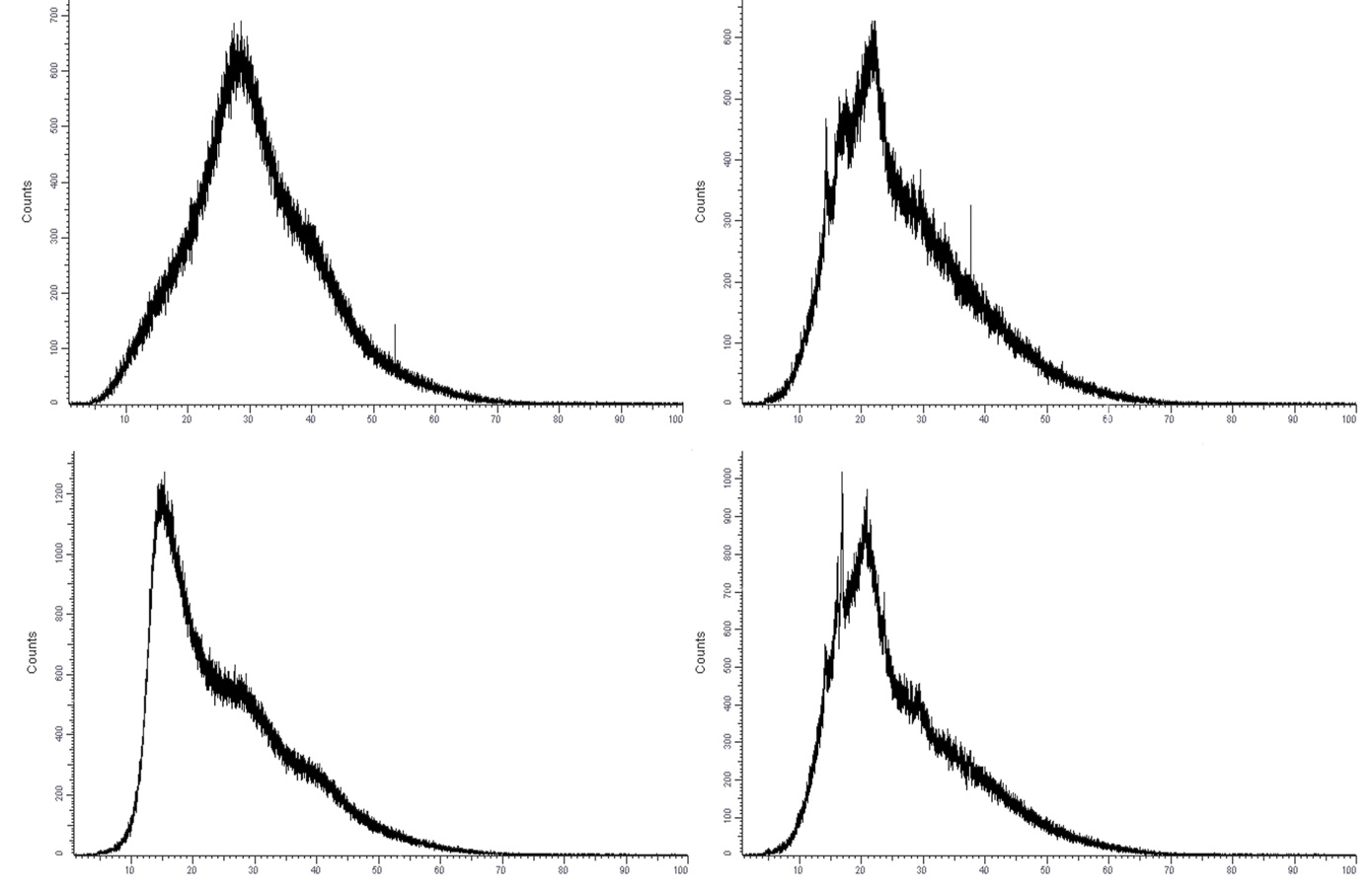 | **2**  **4** |
| --- | --- | --- |

| **i** | 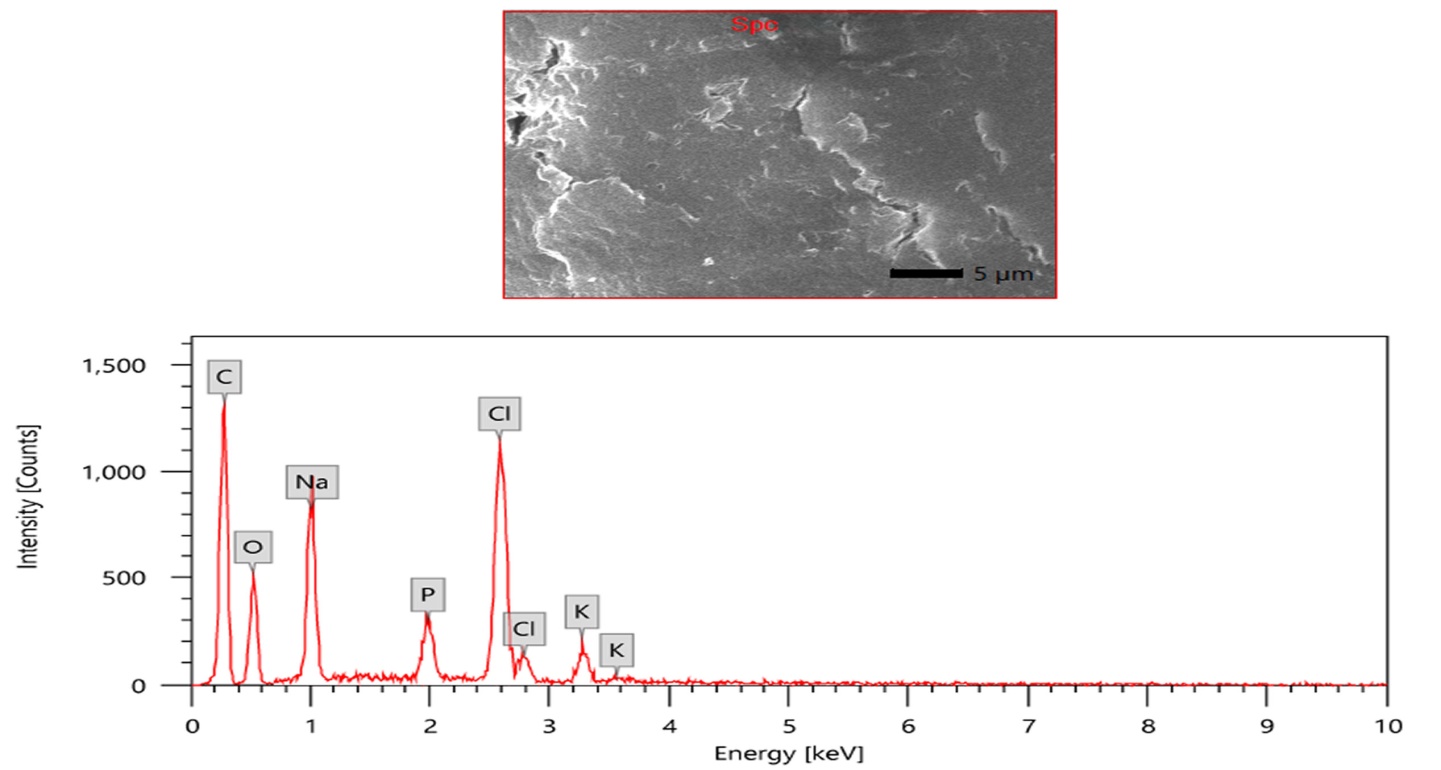 |
| --- | --- |

**Fig (2): CPL-Micelles NP Characterization; a-d** (TEM, SEM, particle size, zeta potential of CPL-Micelles NP), **e-h** (UV-Vis spectra, PL, FTIR transmittance and absorbance, **XRD** of (**1.** CPL-MicellesNP, **2.** Cardamom, **3.** Pistacia, **4.** Laurel), **i.** **EDX** of CPL-Micelles NP.

| **a** | 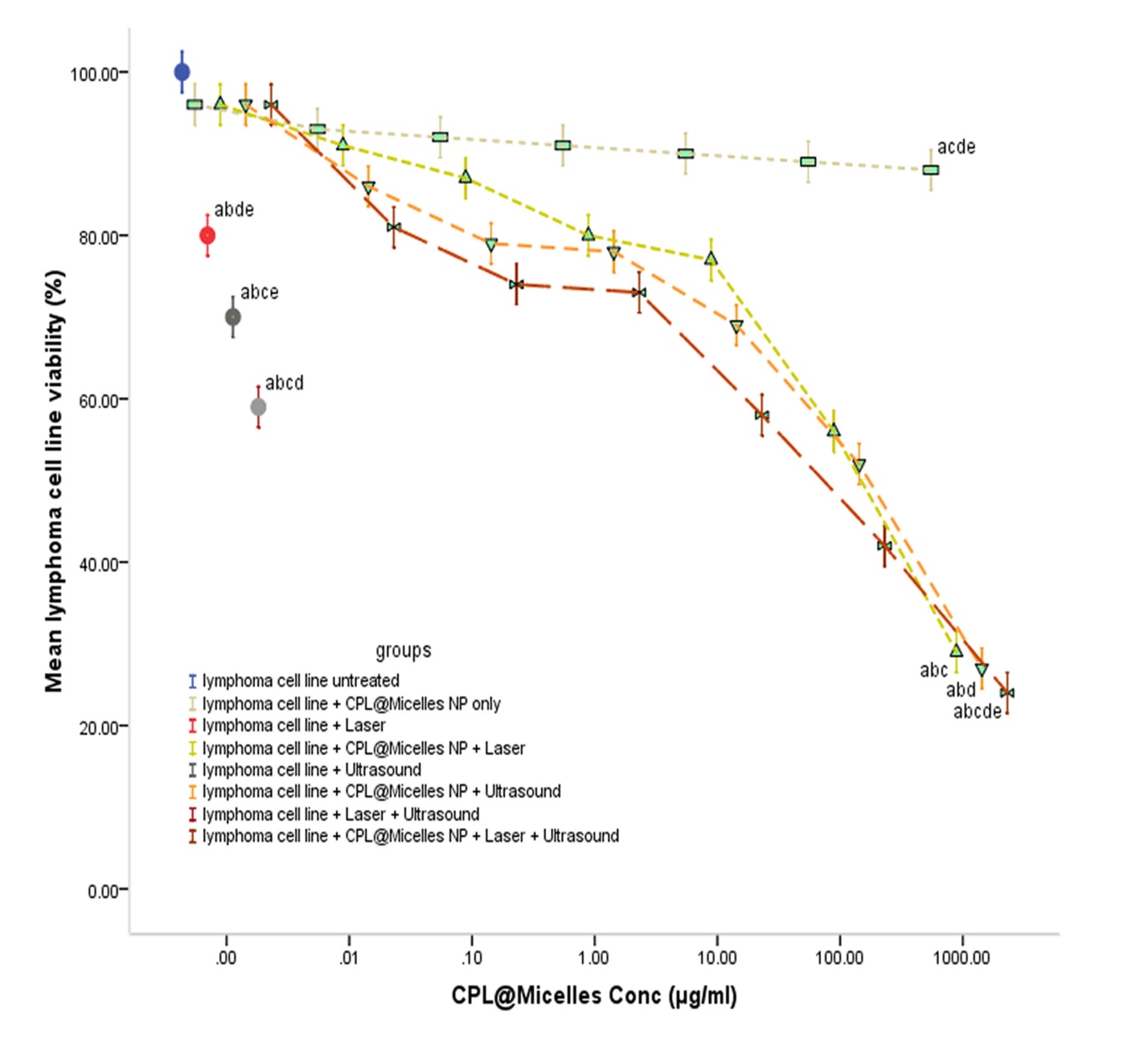 |
| --- | --- |

**Fig (3a): The impact of various treatment approaches on the viability of prostate cancer (U-937) cells;** In all in vitro research groups, cells were subjected to several treatment modalities including serial dilution of CPL-Micelles NP for 24 hours, a. microscopic examinations, and b. dosage response curves. The WST-1 test was applied to assess cell viability. **Viability of cells (%):** F(p) = 4.718 (<0.001*). The data (n=3) are shown as mean±SD. ^a,b,c,d,e^ Significant with (untreated prostate cancer group, non activated CPL-Micelles NP treated group, laser subjected group, ultrasound subjected group, laser+ultrasound group).

| **a** | 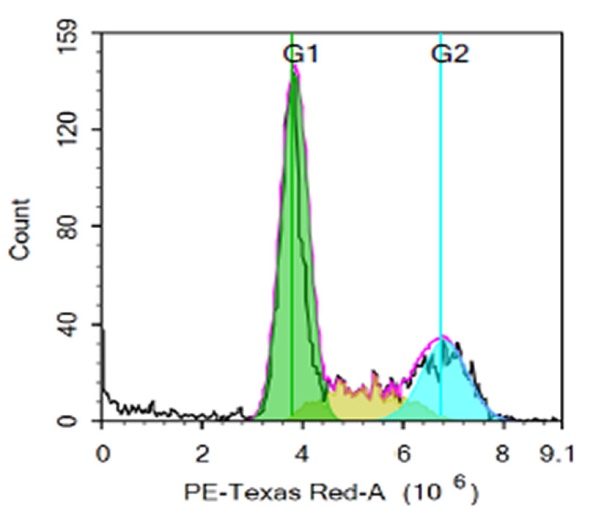**1** | 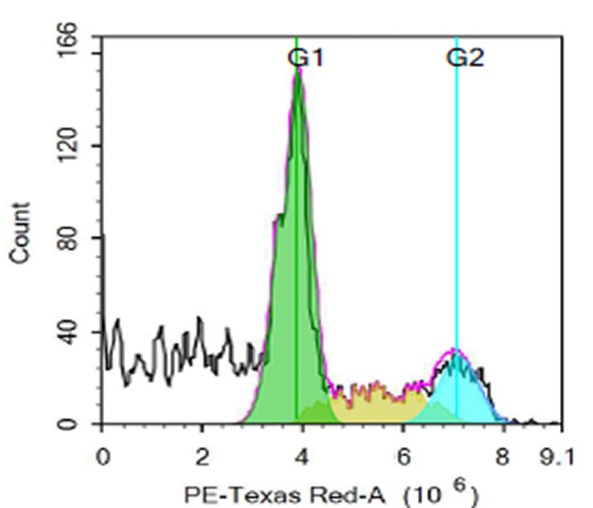**2** | |  |
| --- | --- | --- | --- | --- |
|  | 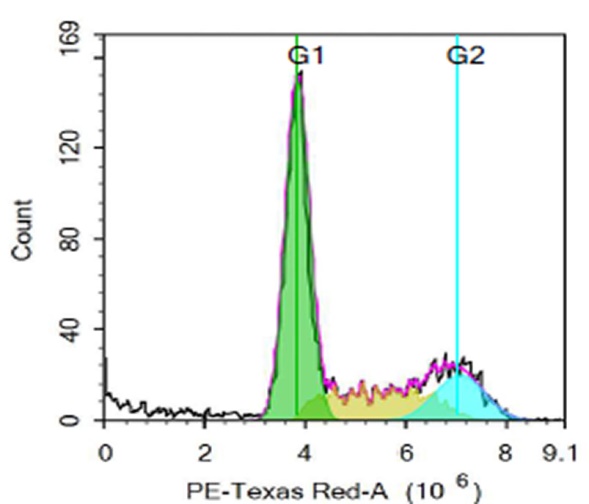**3** | | 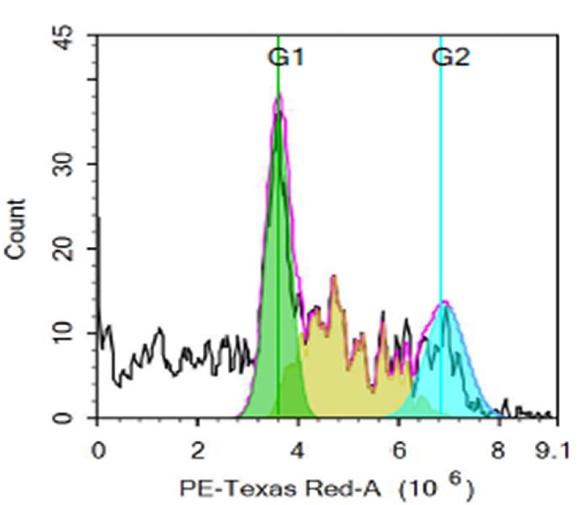**4** | |
|  | 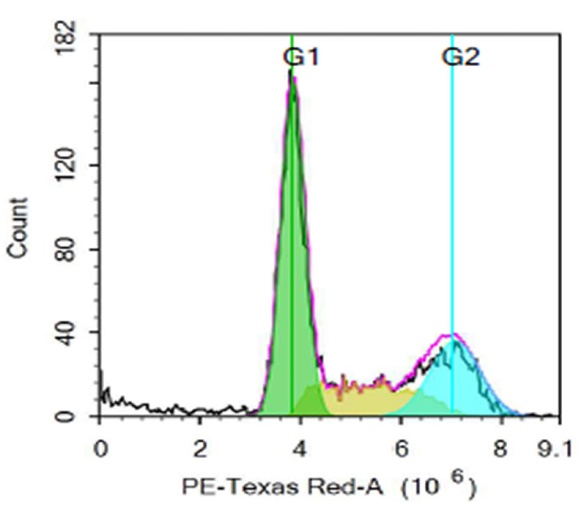**5** | | 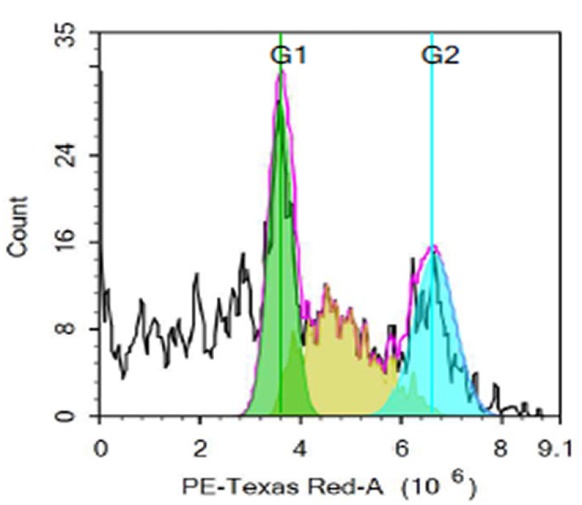**6** | |
|  | 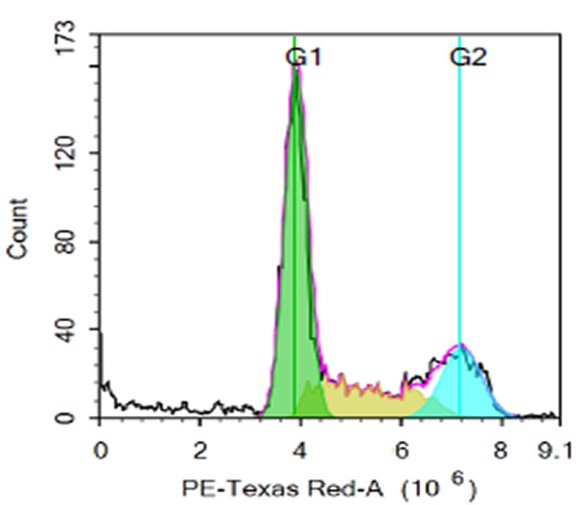**7** | | 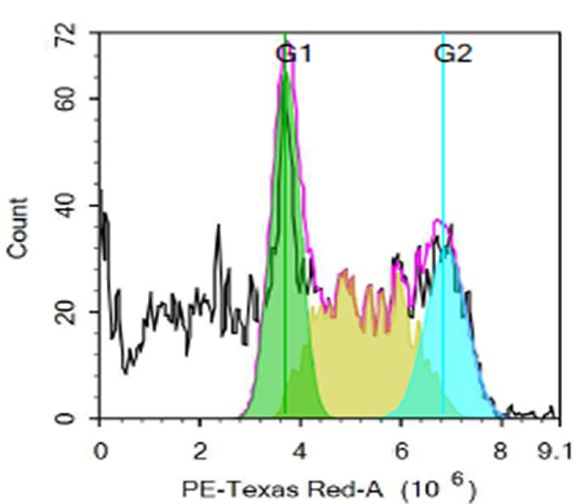**8** | |
| **b** | 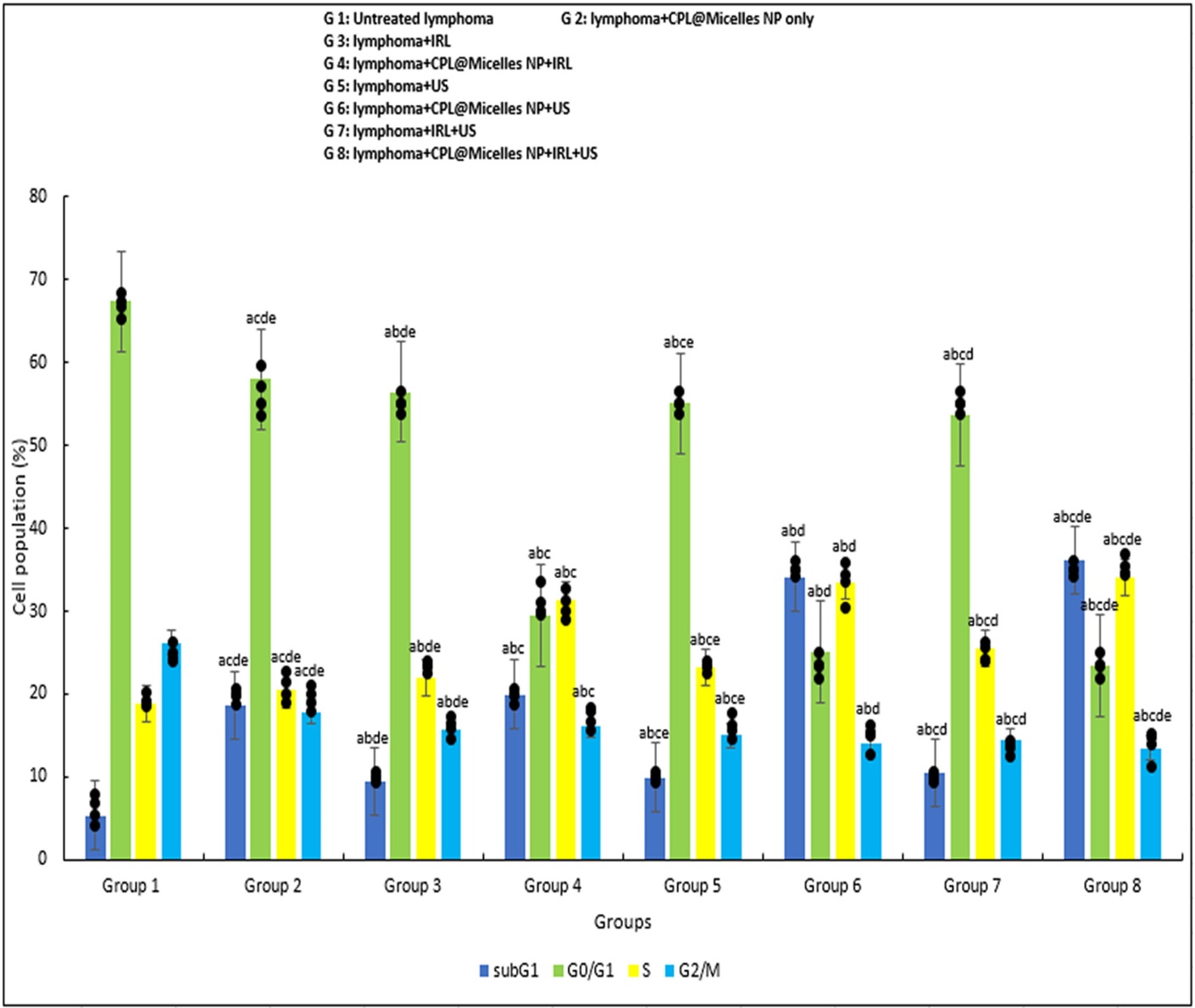 | | | |

**Fig (3b): The impact of various treatment approaches on the distribution of lymphoma (U-937) cell cycles throughout all in vitro research groups;** 1. For a 24-hour period, cells were subjected to various treatment modalities; 2. DNA cytometry analysis was applied to assess the cell cycle distribution, and the proportion of total events for each phase of the cell was plotted; **SubG1,** **G0/G1, S, G2/M (%):**F(p)= 637.920 (<0.001*), 432.574 (<0.001*), 78.846 (<0.001*), 38.112 (<0.001*).The data (n=3) are shown as mean±SD. ^a,b,c,d,e^ Significant with (untreated lymphoma group, non activated CPL-Micelles NP treated group, laser subjected group, ultrasound subjected group, laser+ultrasound group).

| **a** | 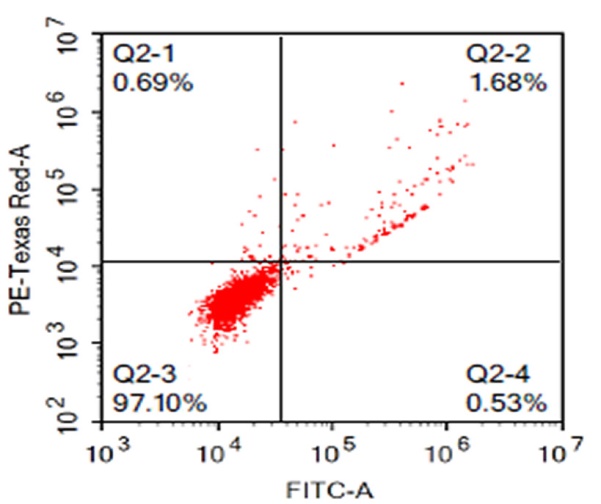**1** | 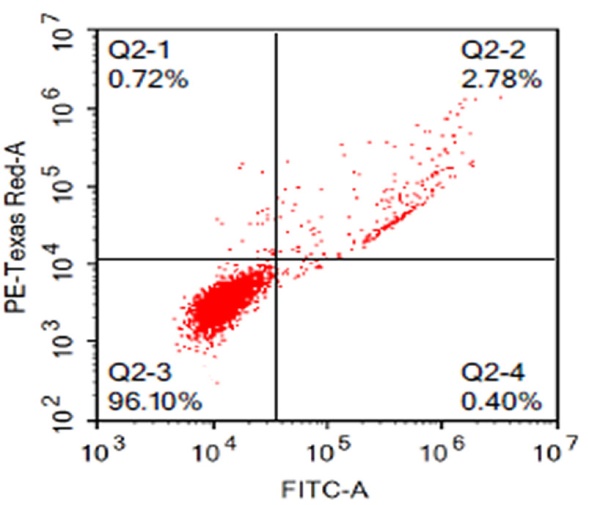**2** |
| --- | --- | --- |
|  | 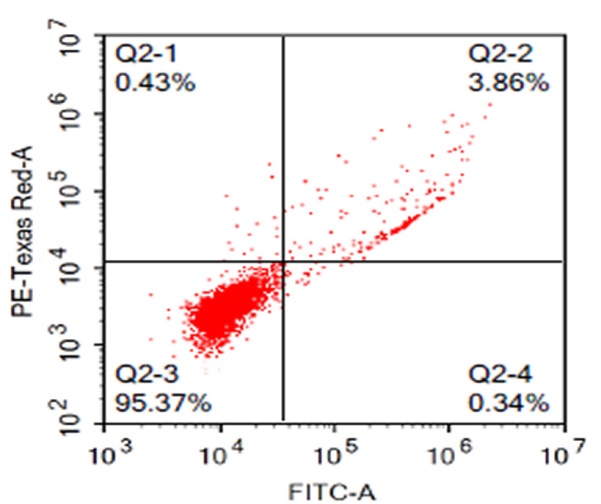**3** | 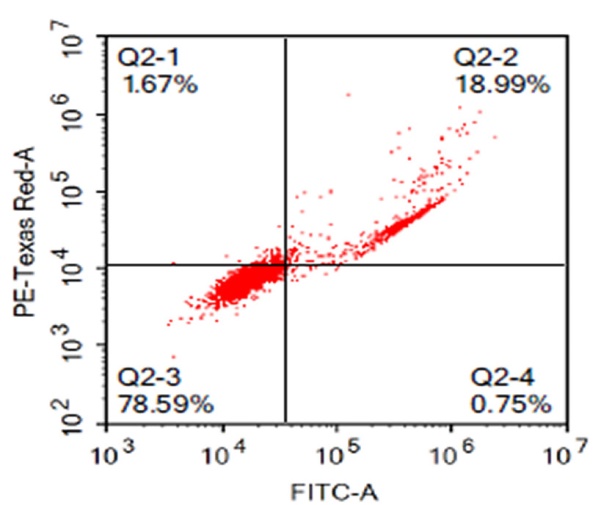**4** |
|  | 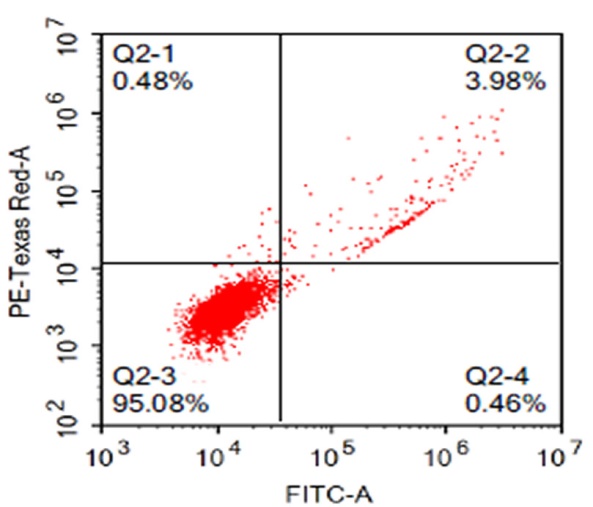**5** | 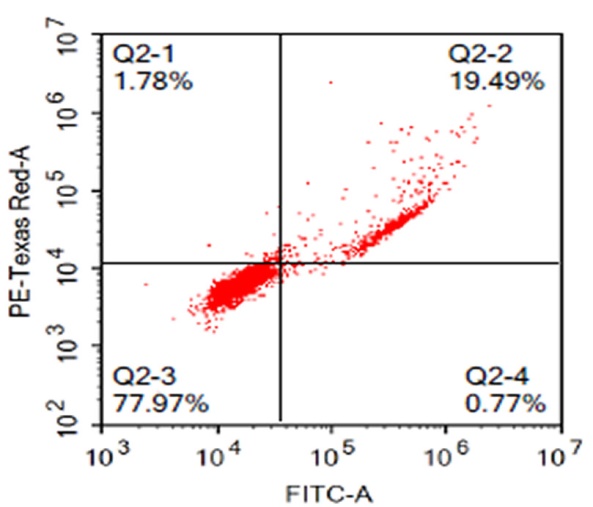**6** |
|  | 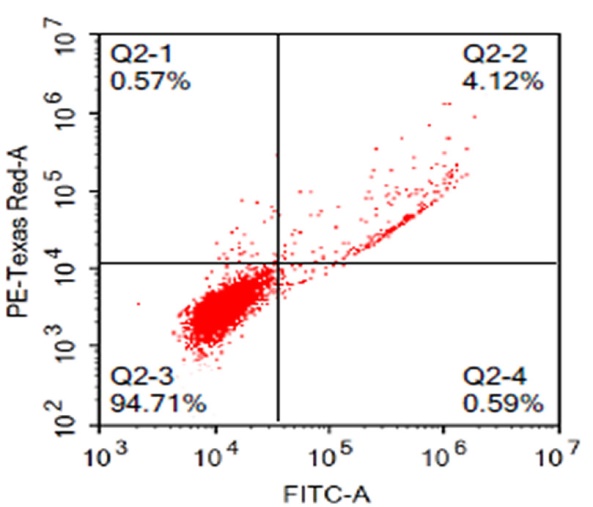**7** | 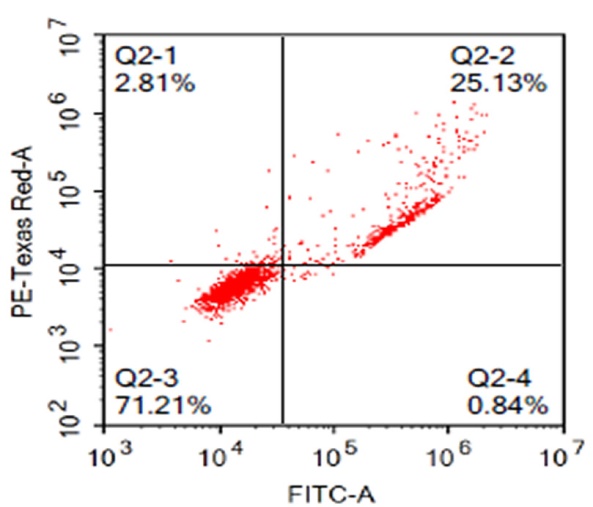**8** |
| **b** | 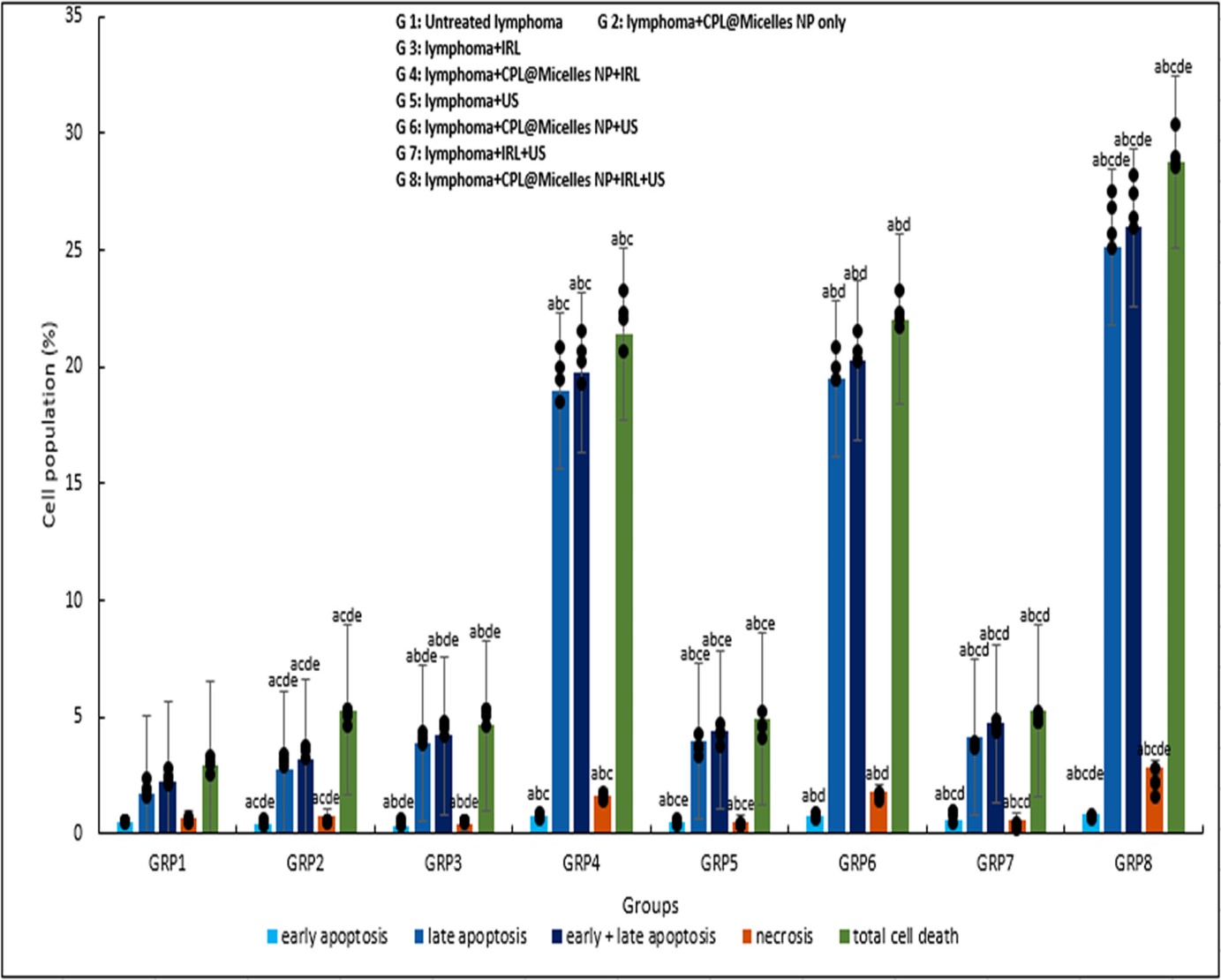 | |

**Fig (3c): The impact of various treatment approaches on necrosis and apoptosis of lymphoma (U-937) across all in vitro research groups; 1.** For 24 hours, cells were subjected to various treatment modalities; **2.** Annexin V-FITC/PI was applied to stain the cells, and various cell populations were plotted as a proportion of the total events. **early apoptosis**, **late apoptosis**, **early and late apoptosis**, **total cell death**, **necrosis:** F(p)= 6.592 (<0.001*), 900.167 (<0.001*), 1.044E3 (<0.001*), 43.717 (<0.001*), 1.166E3 (<0.001*). The data (n=3) are shown as mean±SD. ^a,b,c,d,e^ Significant with (untreated lymphoma group, non activated CPL-Micelles NP treated group, laser subjected group, ultrasound subjected group, laser+ultrasound group).

| **a** | 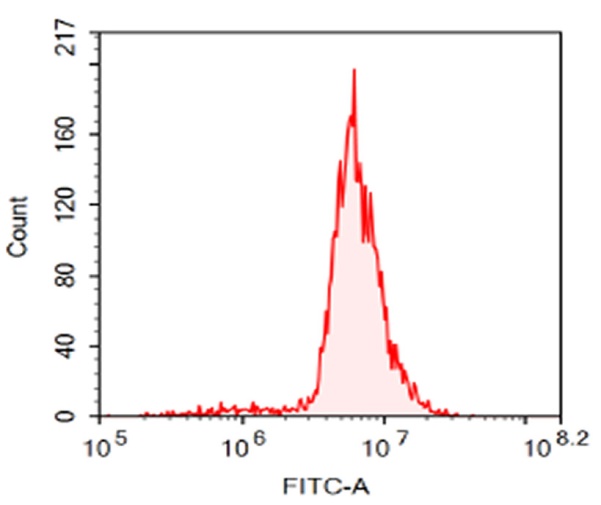**1** | 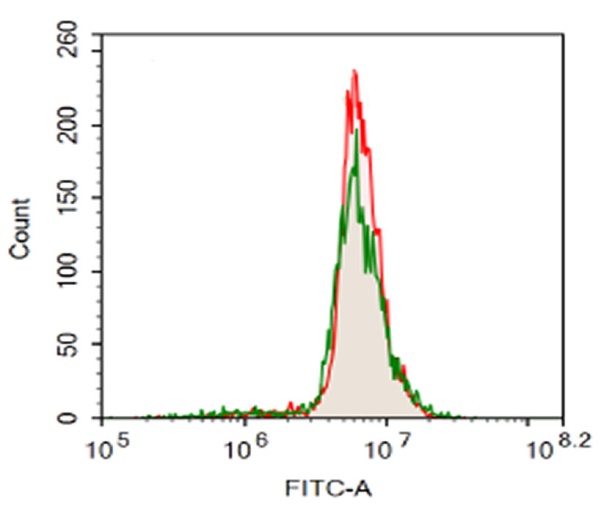**2** |
| --- | --- | --- |
|  | 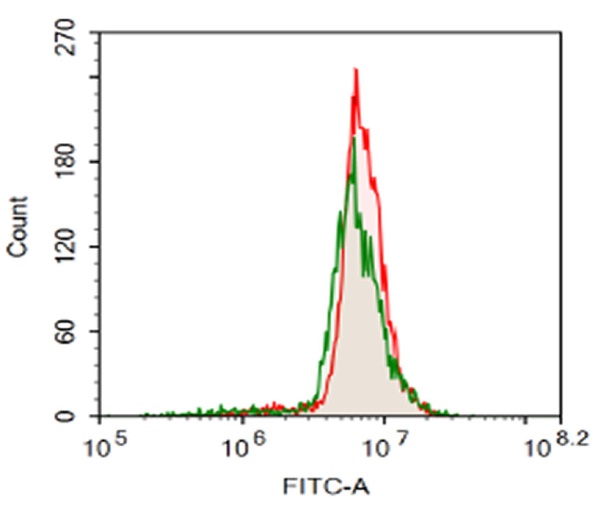**3** | 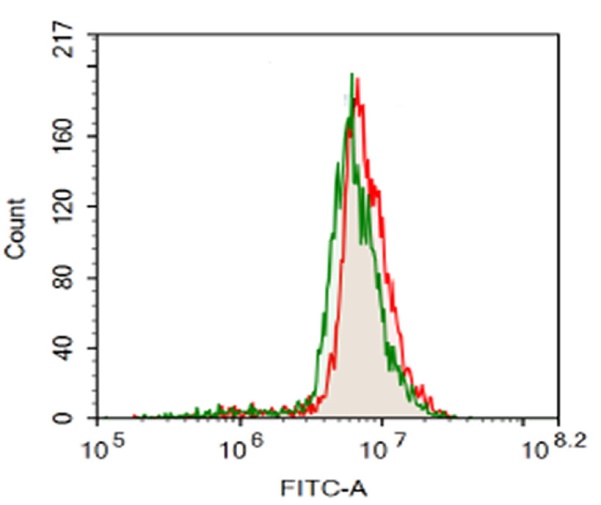**4** |
|  | 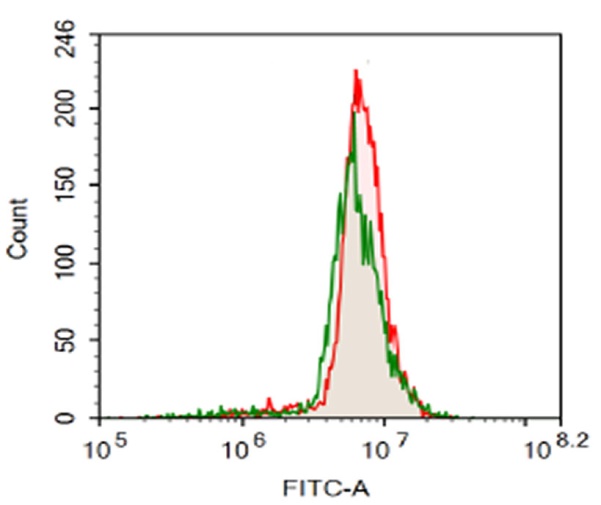**5** | 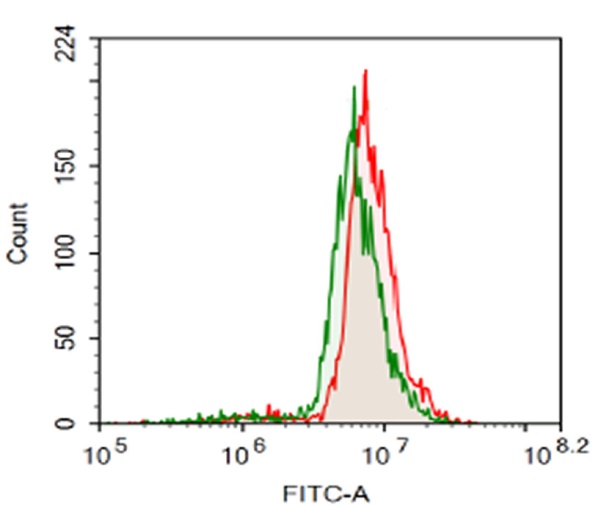**6** |
|  | 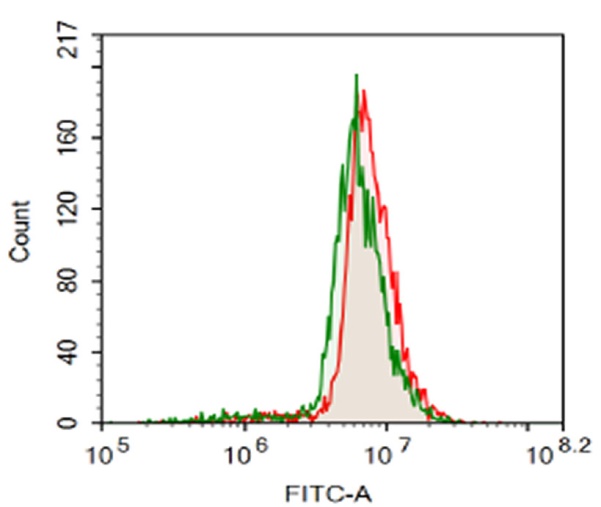**7** | 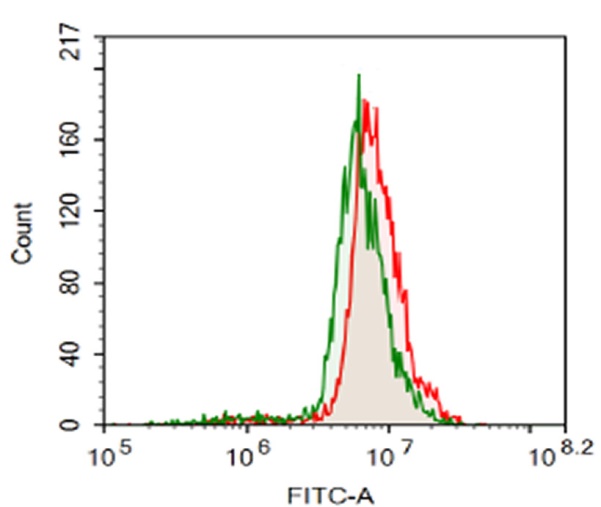**8** |
| **b** | 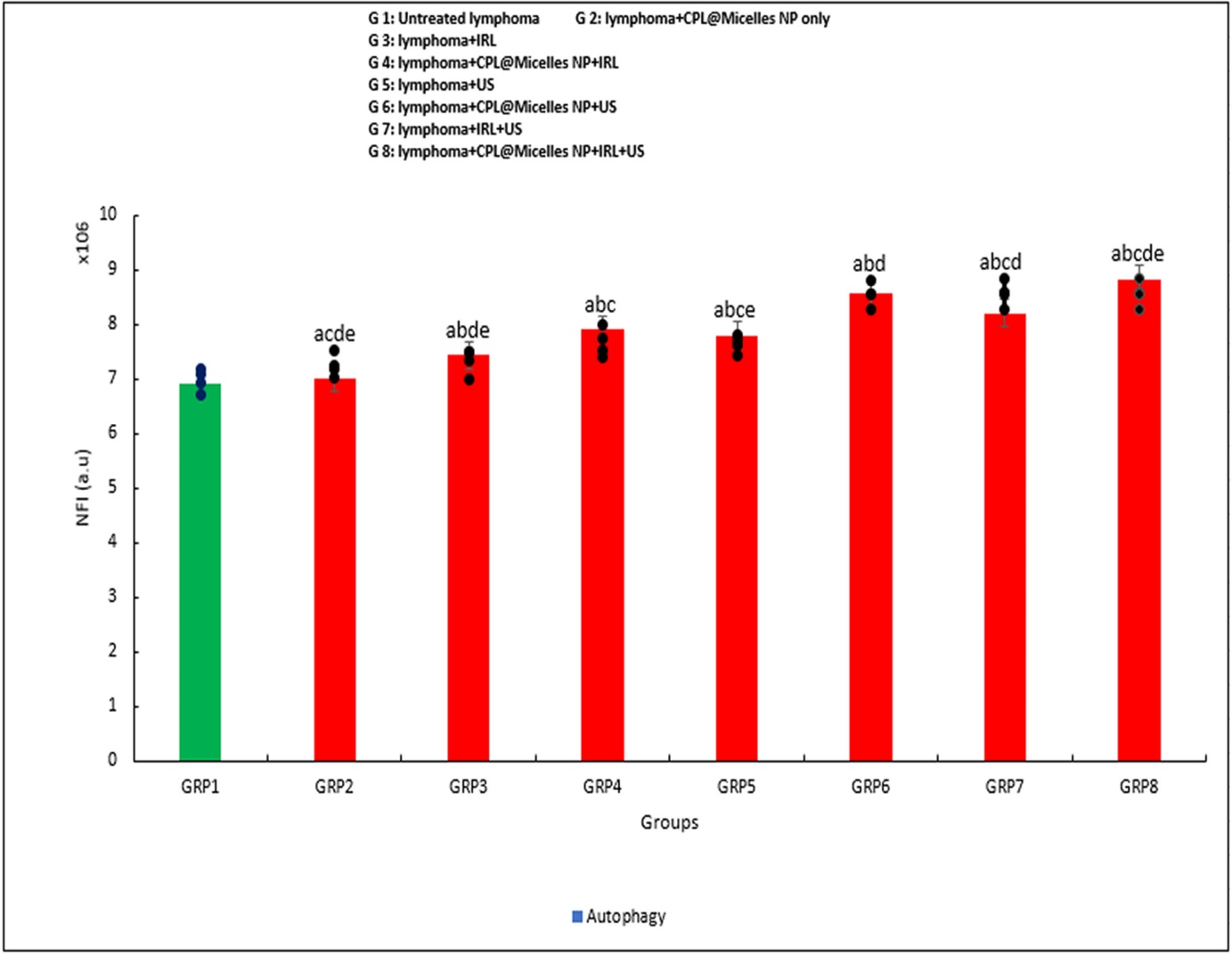 | |

**Fig (3d): The impact of various treatment approaches on the autophagy of lymphoma (U-937) across all in vitro research groups;** **1.** After 24 hours of exposure to various treatment modalities, cells were labeled using Cyto-ID autophagosome tracker. **2.** Plotting of net fluorescent intensity (NFI; red color) was done in comparison to the control group's basal fluorescence (green color). **Autophagy (%):** F(p)= 19.124 (<0.001*). The data (n=3) are shown as mean±SD. ^a,b,c,d,e^ Significant with (untreated lymphoma group, non activated CPL-Micelles NP treated group, laser subjected group, ultrasound subjected group, laser+ultrasound group).

| **1** | 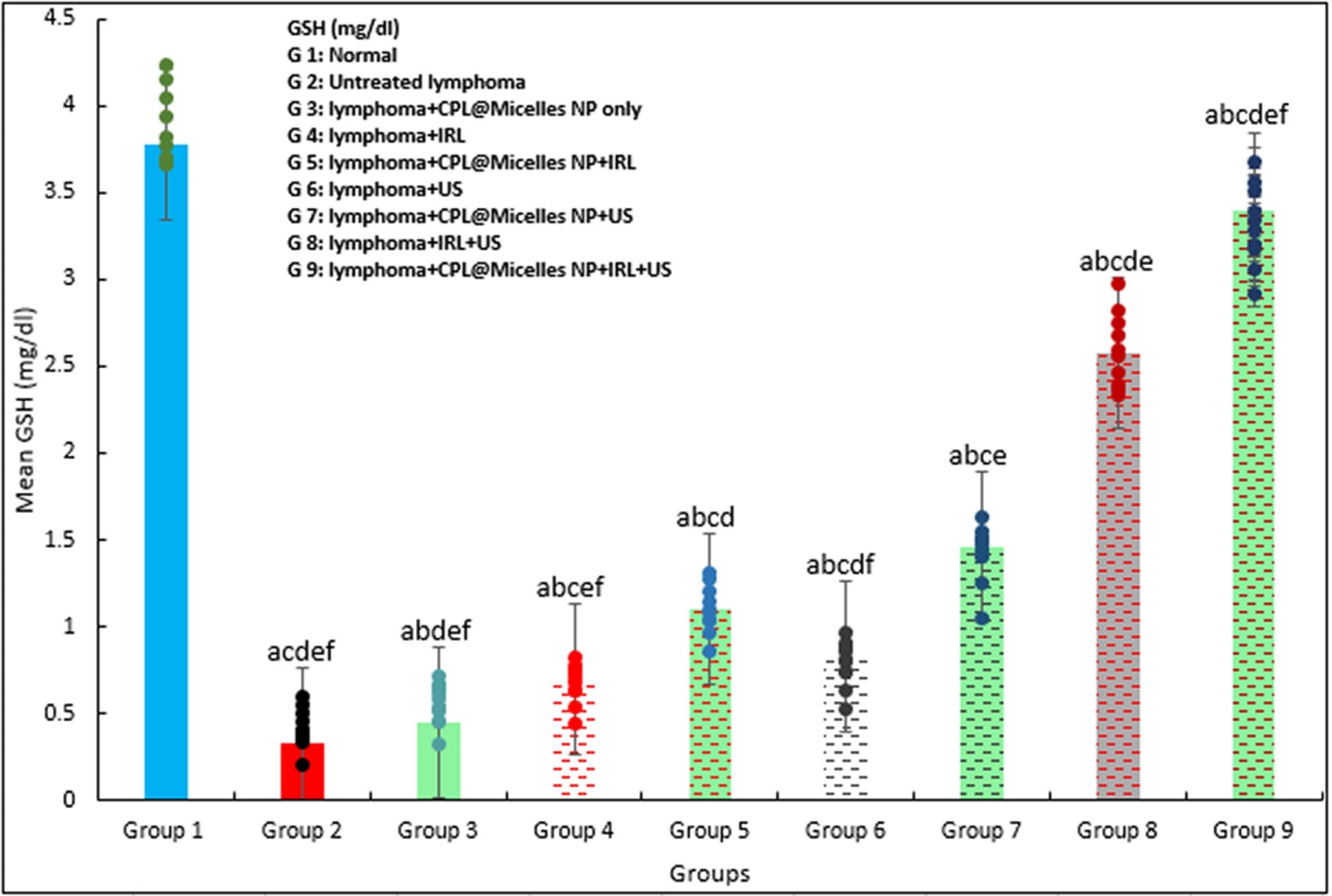 |
| --- | --- |
| **2** | 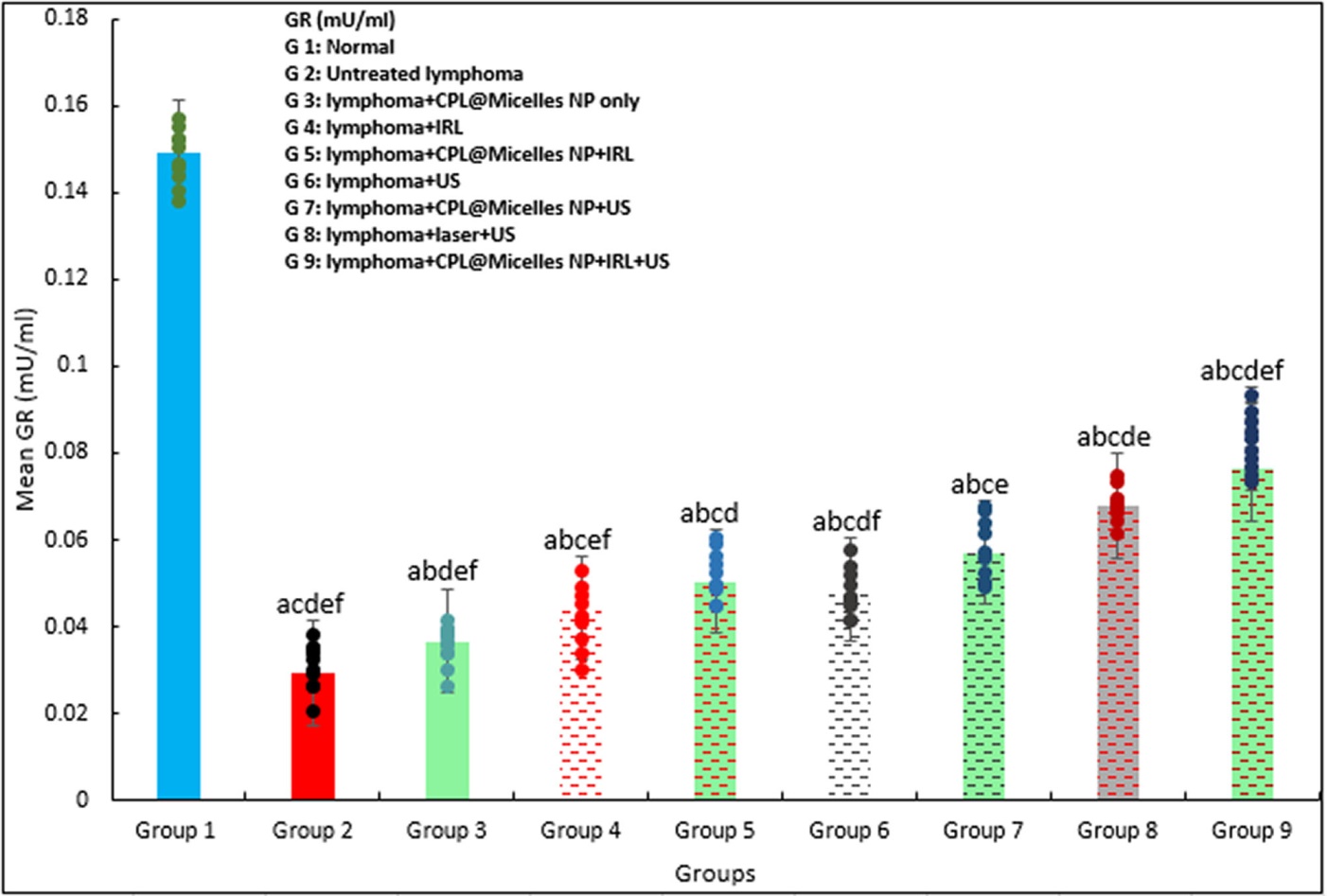 |
| **3** | 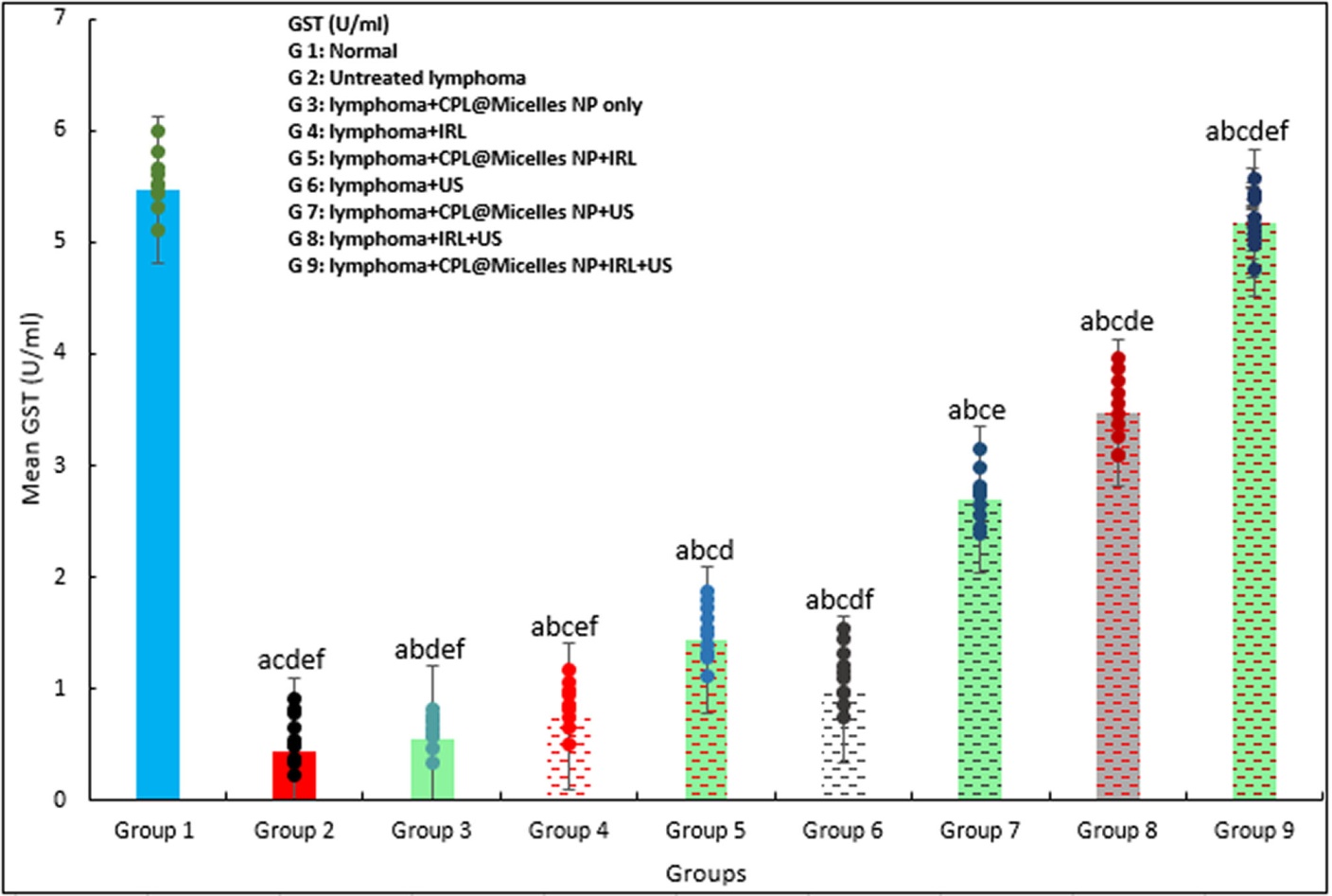 |
| **4** | 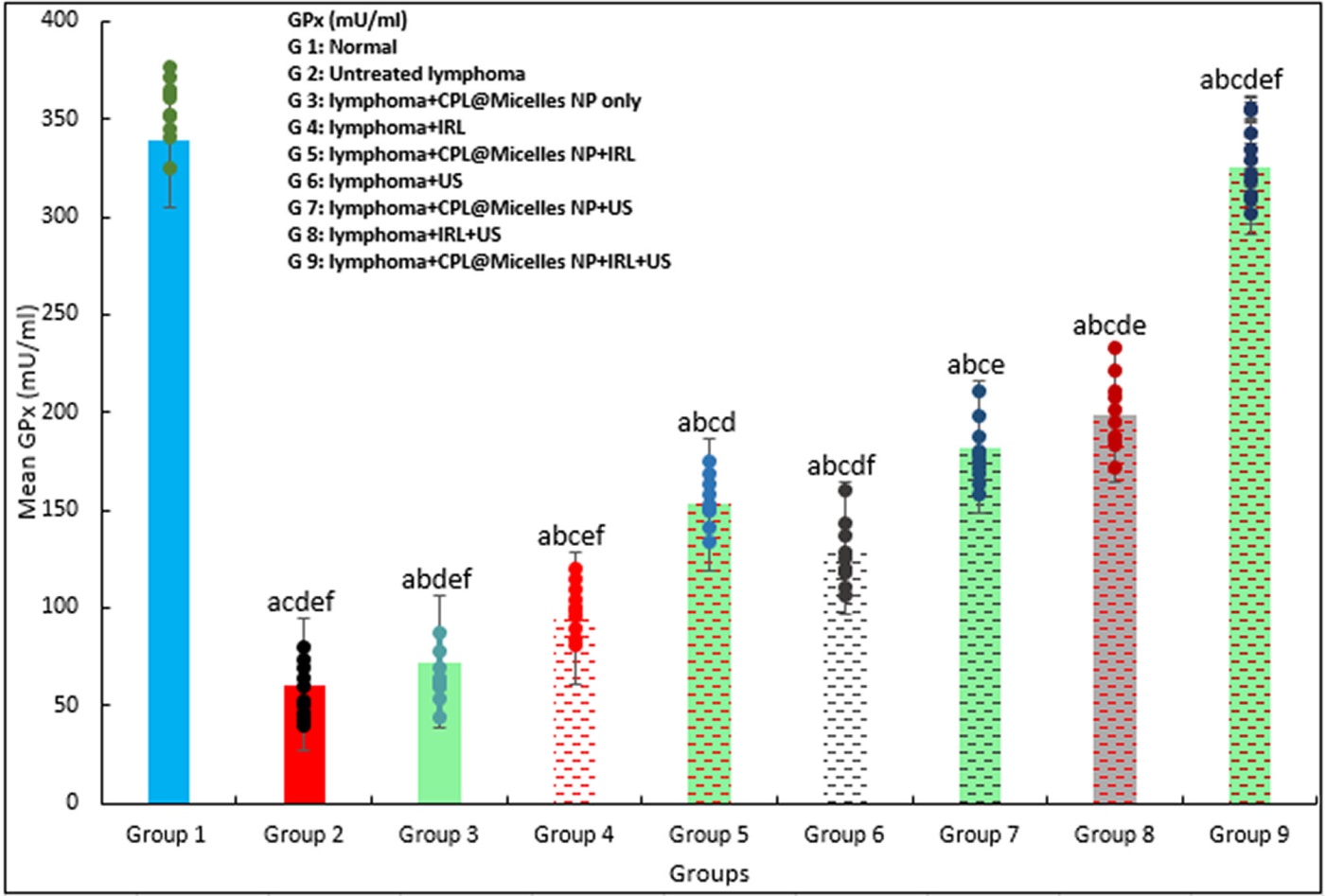 |
| **5** | 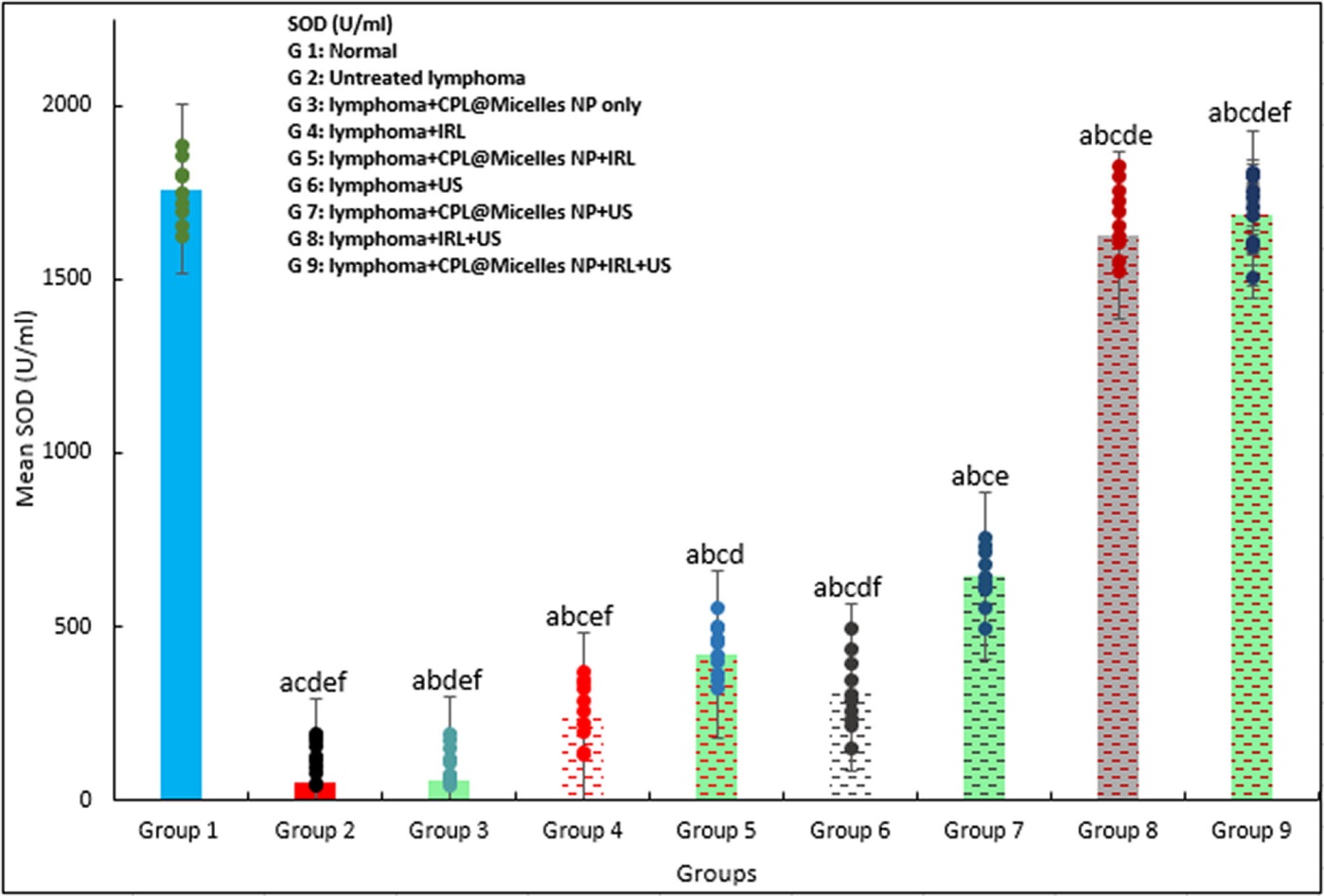 |
| **6** | 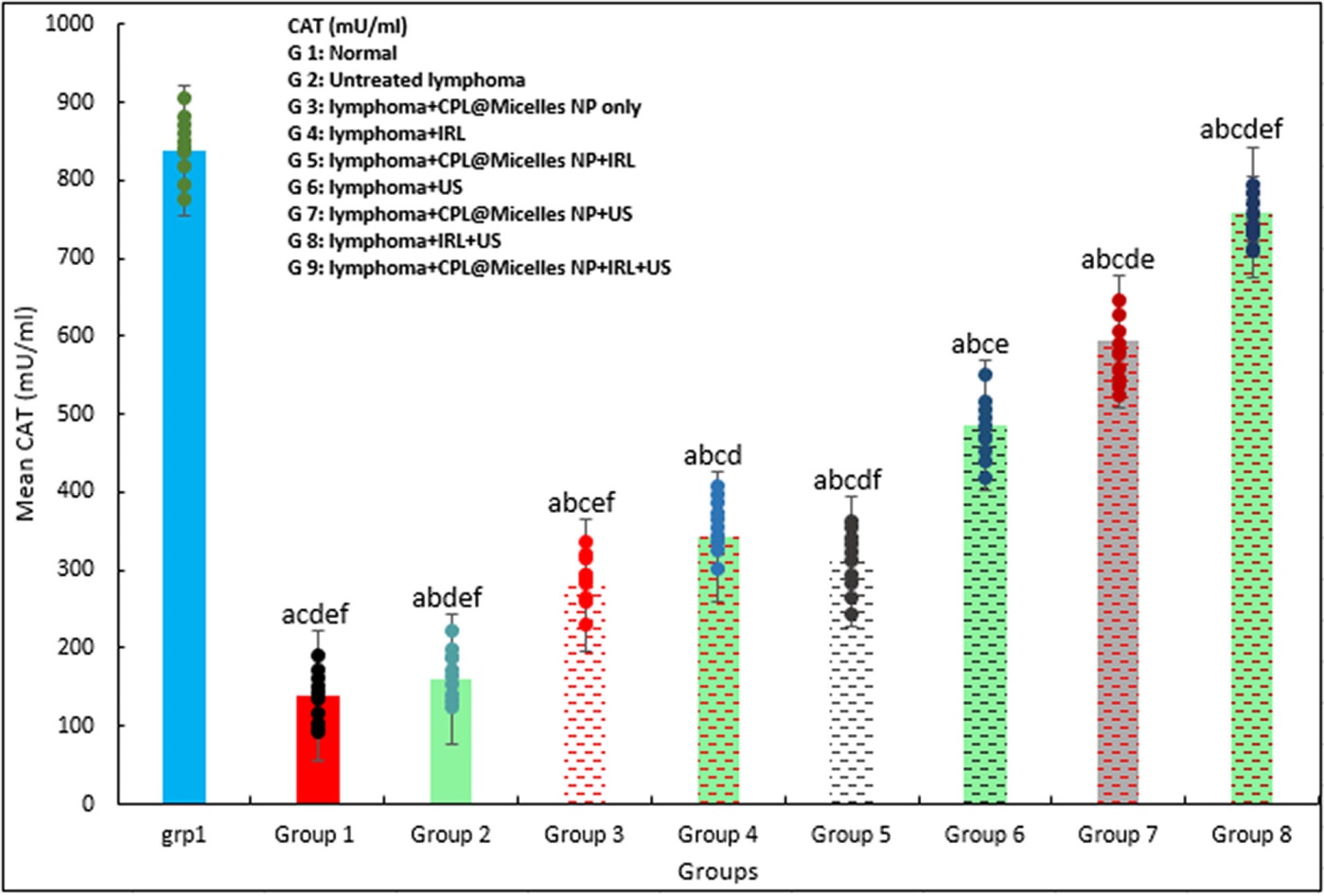 |
| **7** | 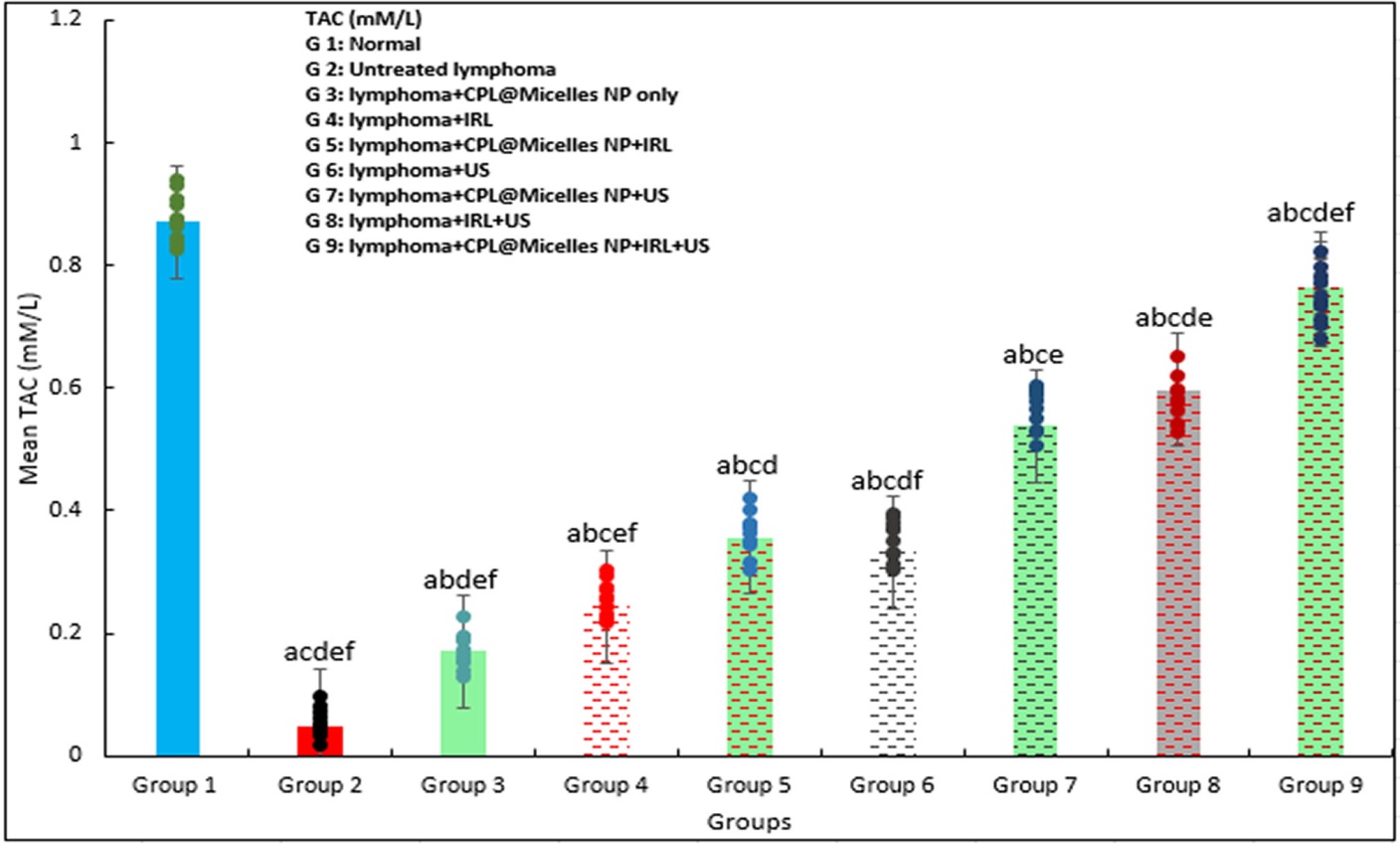 |
| **8** | 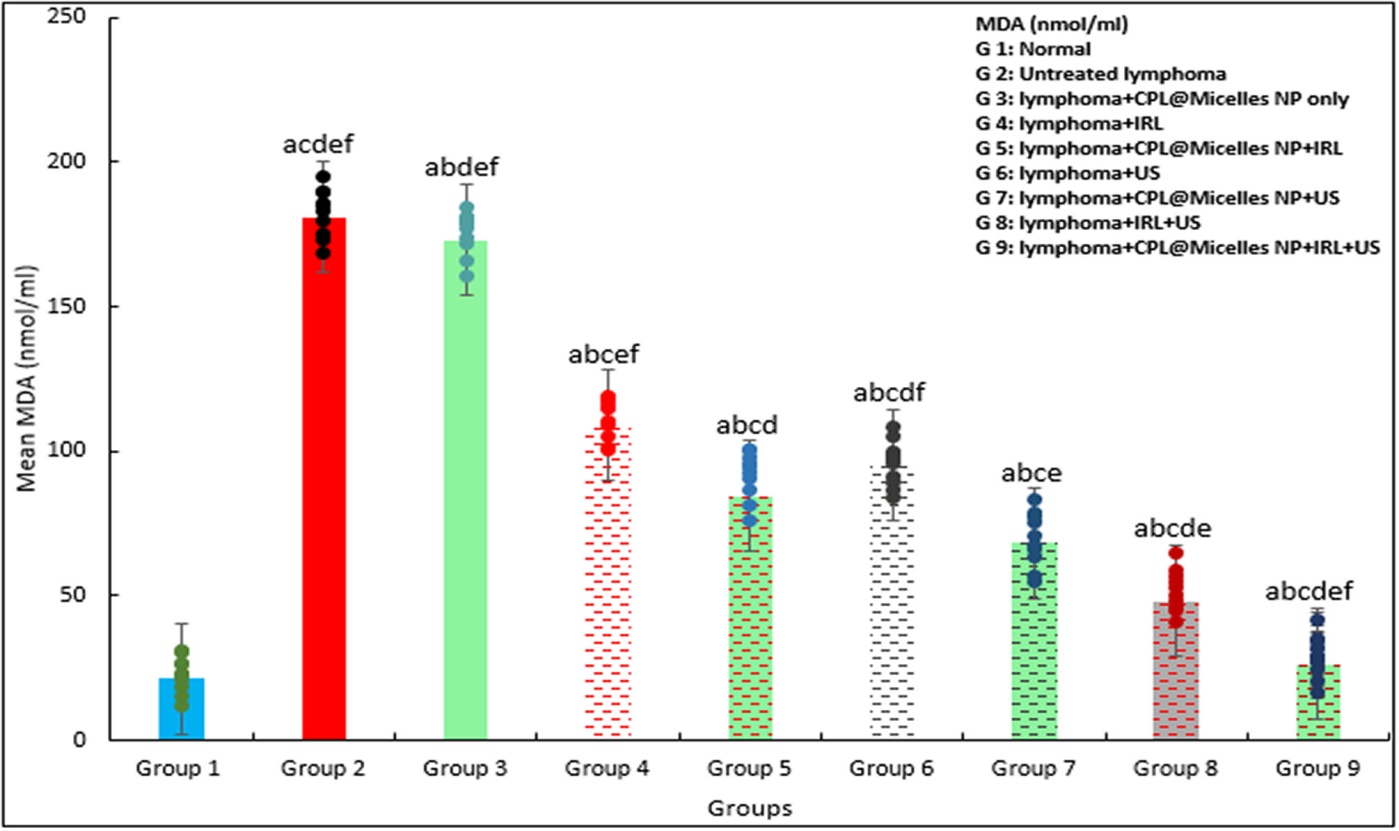 |

**Fig (4a): The impact of various treatment approaches on MDA, antioxidant activities, and capacity across all research groups;** F represents the ANOVA test value. **1-8**. **GR (mU/ml),** **GSH (mg/dl),** **GST (U/ml),** **GPx (mU/ml), SOD (U/ml),** **CAT (mU/ml),** **TAC (mM/L),** **MDA (nmol/ml):** F(p)= 105.046 (<0.001*), 408.814 (<0.001*), 1.217E3 (<0.001*), 2.703E4 (<0.001*), 3.450E6 (<0.001*), 1.721E5 (<0.001*), 3.328E3 (<0.001*), 9.669E4 (<0.001*). The data (n=10 each group) are shown as mean±SD. ^a,b,c,d,e^ Significant with (untreated lymphoma group, non activated CPL-Micelles NP treated group, laser subjected group, ultrasound subjected group, laser+ultrasound group).

| **1** | 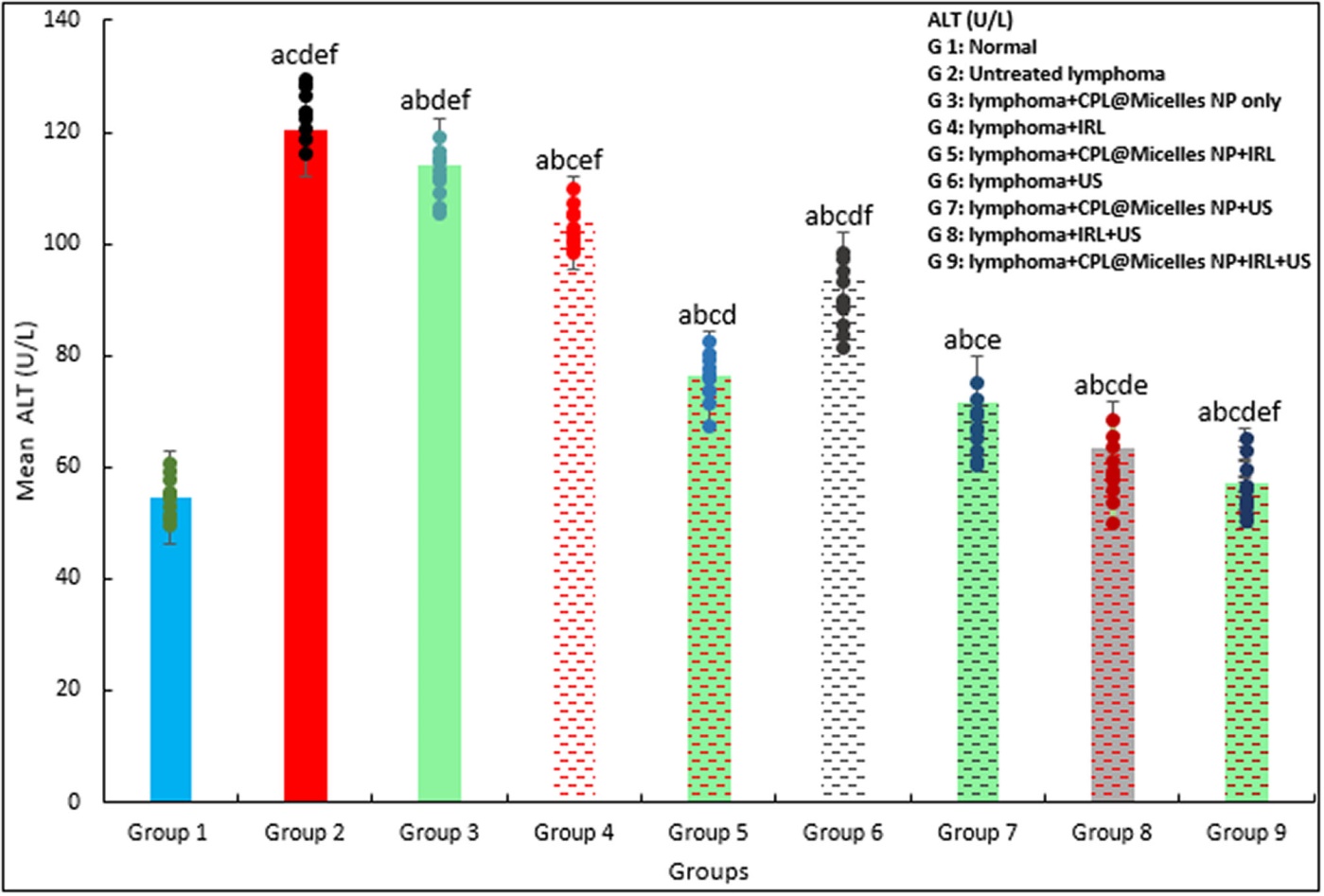 |
| --- | --- |
| **2** | 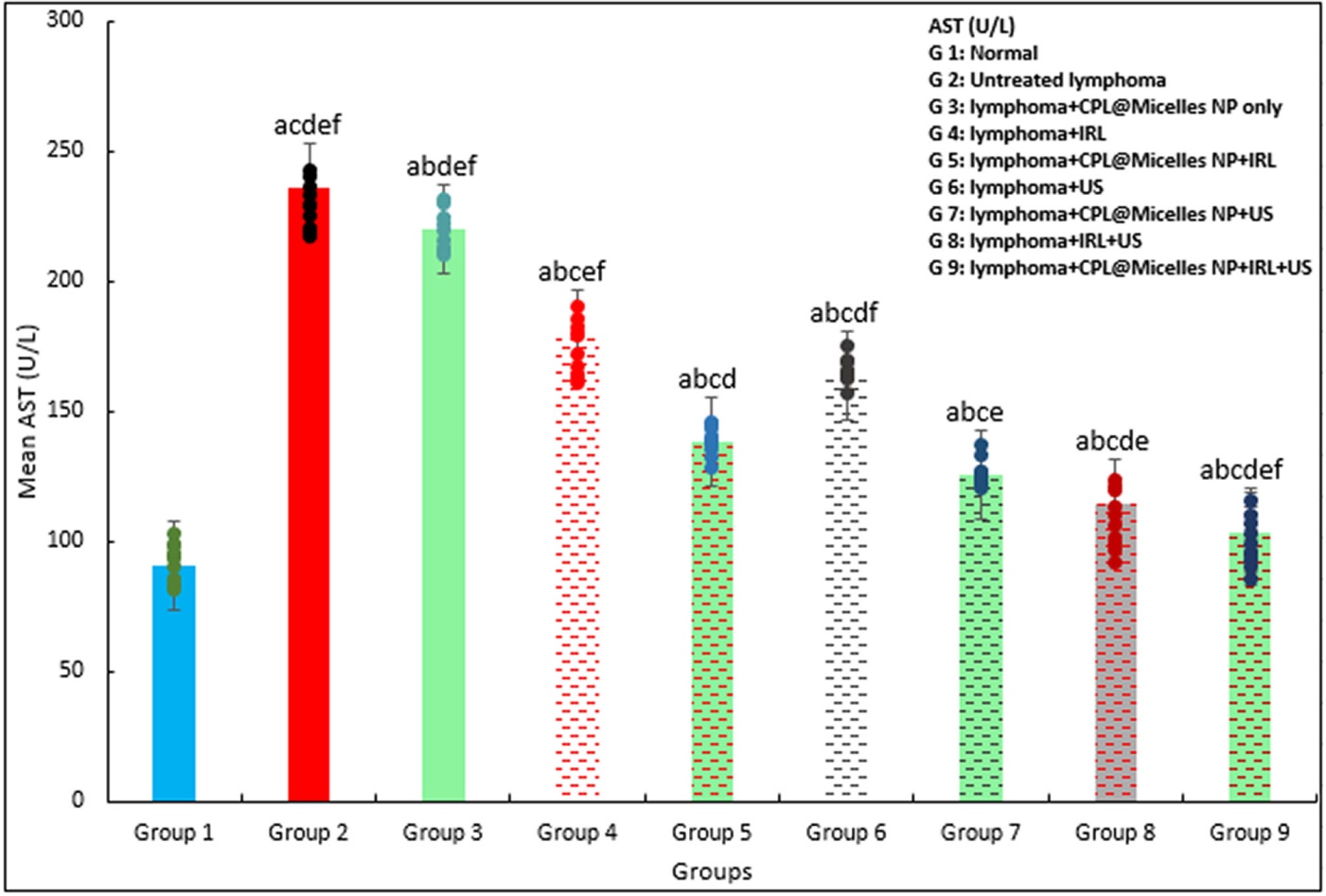 |
| **3** | 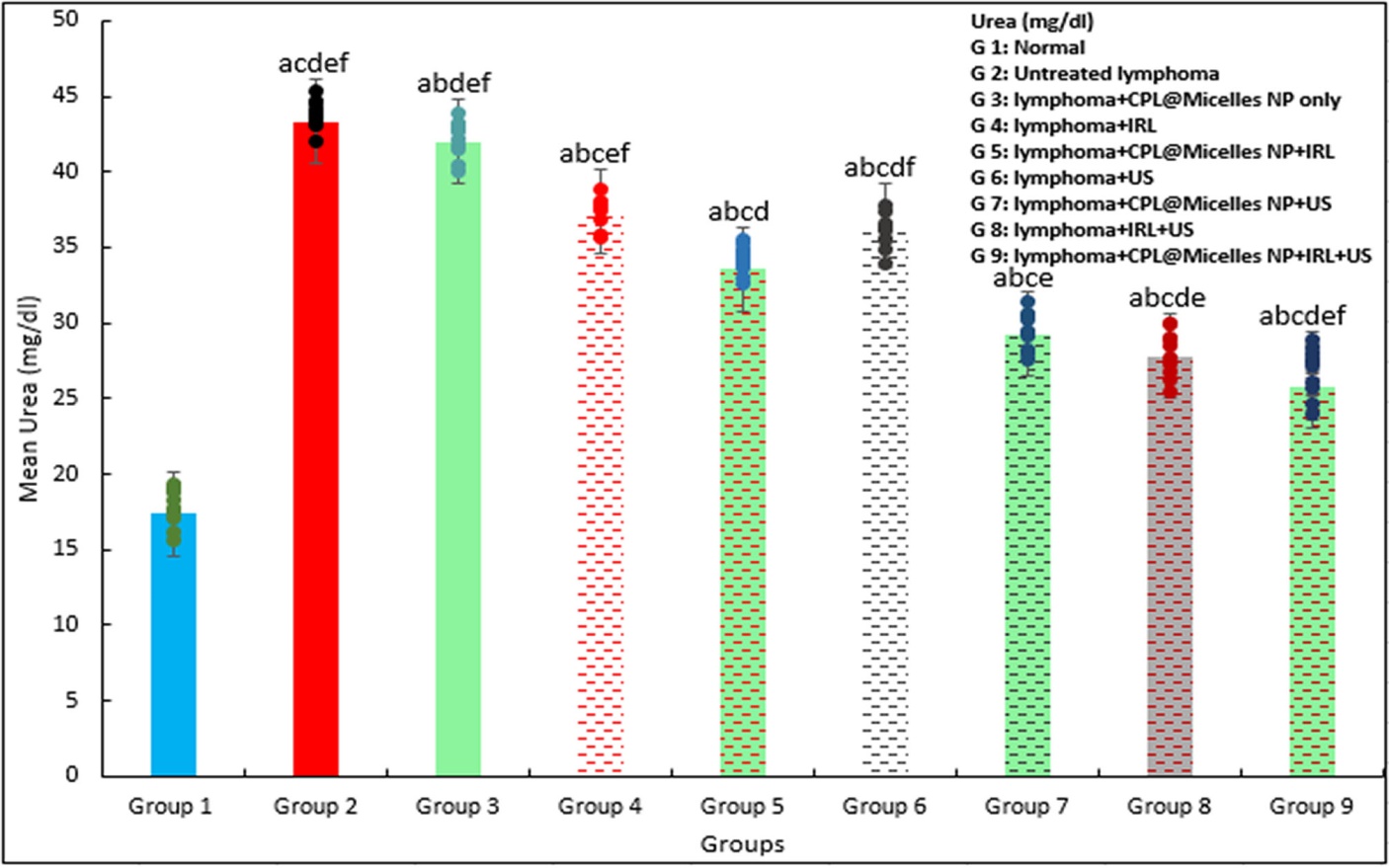 |
| **4** | 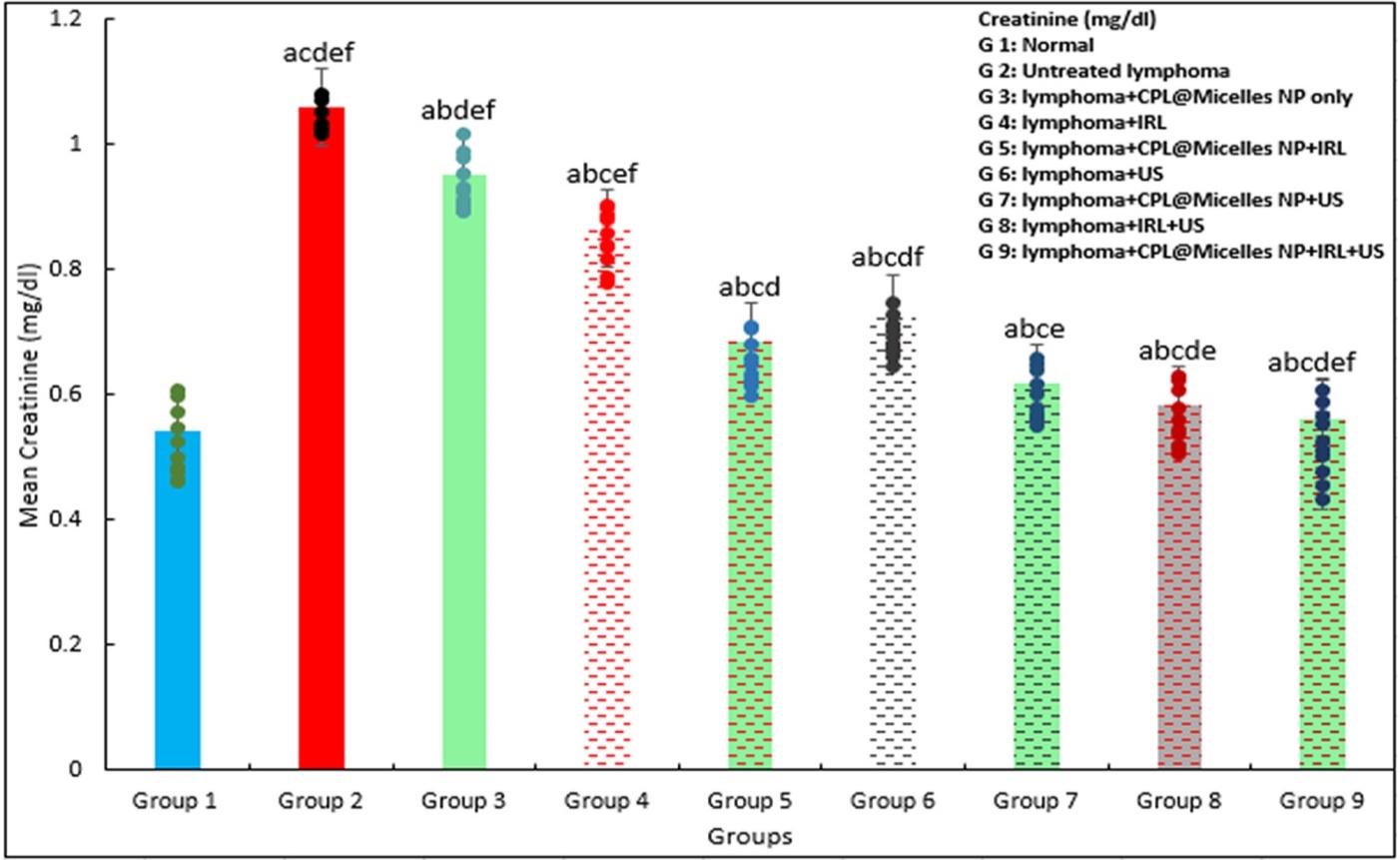 |

**Fig (4b): The impact of various treatment approaches on hepatic and renal biomarkers across all research groups;** F represents the ANOVA test value. **ALT** **(U/l)**, **AST** **(U/l), urea (mg/dl), creatinine** **(mg/dl):** F(p)= 5.184E3 (<0.001*), 1.778E5 (<0.001*), 3.853E3 (<0.001*), 316.670 (<0.001*). The data (n=10 each group) are shown as mean±SD. ^a,b,c,d,e^ Significant with (untreated lymphoma group, non activated CPL-Micelles NP treated group, laser subjected group, ultrasound subjected group, laser+ultrasound group).

**
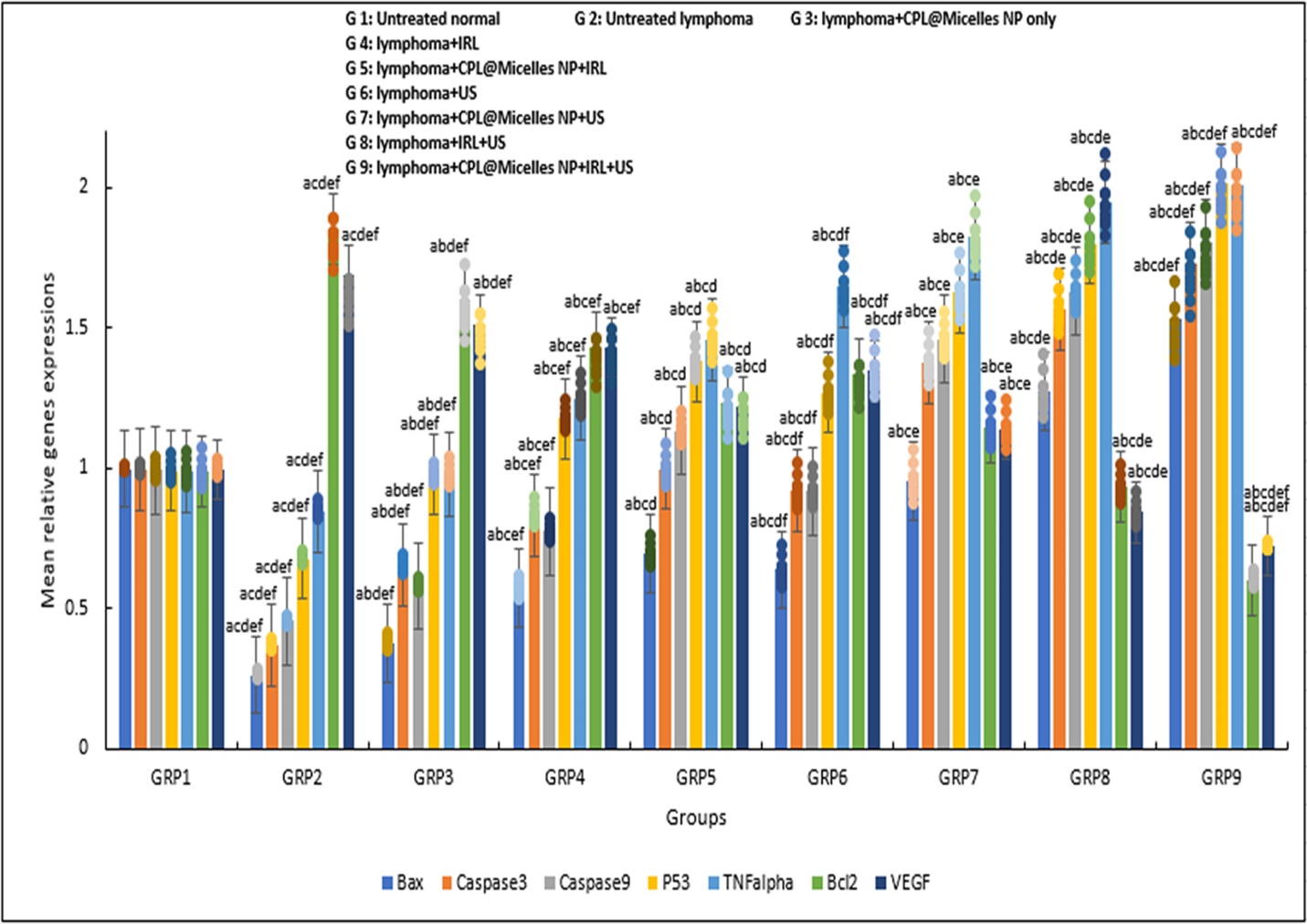
**

**Fig (4c): The impact of various treatment modalities on the gene relative expressions of TNF alpha, Bax, Caspase (9,3), p53, VEGF, and Bcl-2 as measured by qRT-PCR in each research group;** F represents the ANOVA test result. **p53, Bax, Caspase 9,** **Caspase 3, TNFalpha, VEGF,** **Bcl-2**: F(p)= 576.752 (<0.001*), 650.868 (<0.001*), 895.850 (<0.001*), 749.625 (<0.001*), 456.606 (<0.001*), 173.303 (<0.001*), 217.722 (<0.001*). The data (n=10 each group) are shown as mean±SD. ^a,b,c,d,e^ Significant with (untreated lymphoma group, non activated CPL-Micelles NP treated group, laser subjected group, ultrasound subjected group, laser+ultrasound group).

| **Lymphoma induced mice H&E stained section**  **without treatment as a control group** | **Lymphoma induced mice H&E stained section**  **treated with CPL-Micelles NP only** |
| --- | --- |
| 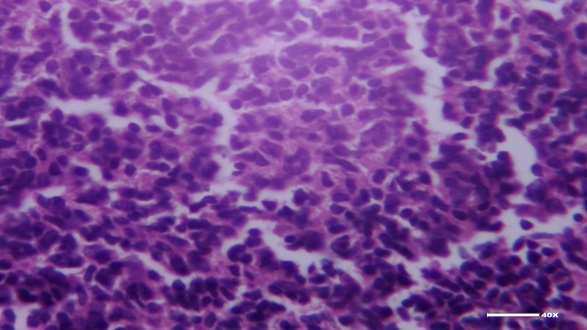 | 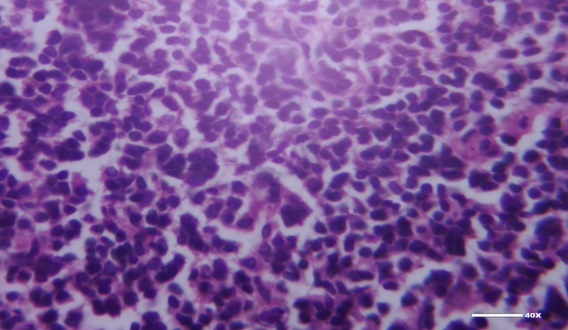 |
| **Lymphoma induced mice H&E stained section**  **treated with IRL** | **Lymphoma induced mice H&E stained section**  **treated with CPL-Micelles NP and irradiated with IRL** |
| 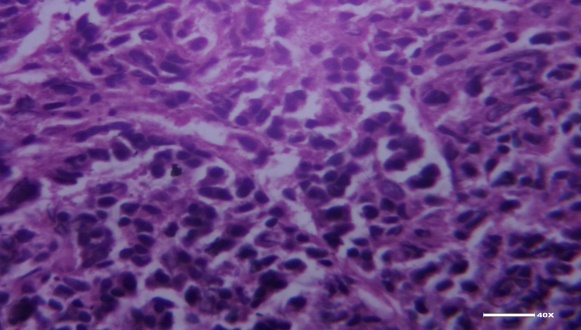 | 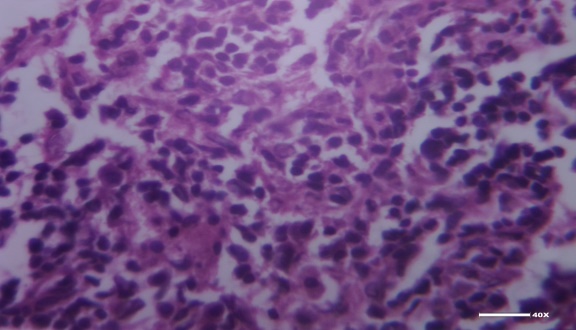 |
| **Lymphoma induced mice H&E stained section**  **treated with US** | **Lymphoma induced mice H&E stained section**  **treated with CPL-Micelles NP and irradiated with US** |
| 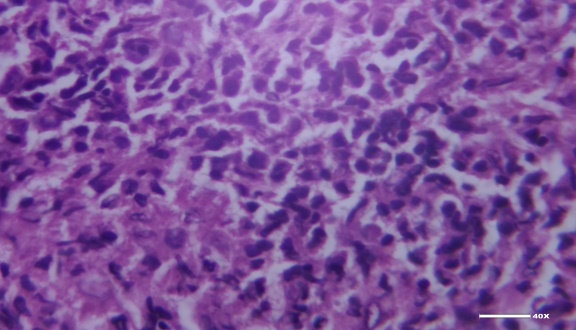 | 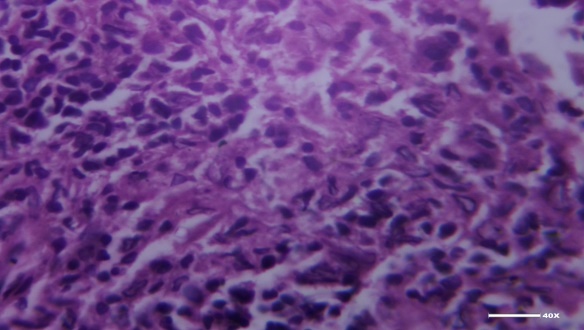 |
| **Lymphoma induced mice H&E stained section**  **treated with IRL and US** | **Lymphoma induced mice H&E stained section**  **treated with CPL-Micelles NP and irradiated with IRL and US** |
| 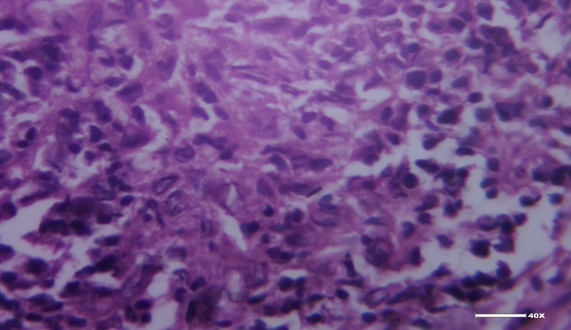 | 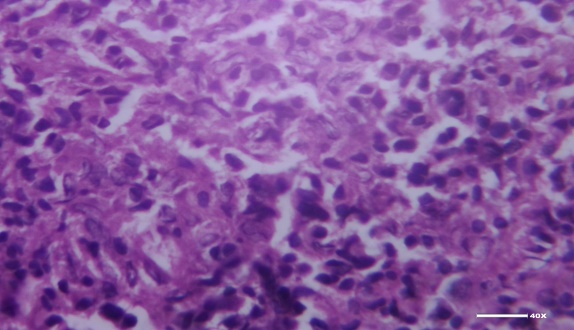 |

**Fig (4d): the H&E-stained segment of lymphoma tissue in all research groups, which illustrates the impact of various treatment regimens at the cellular level (Magnification x 40); 1.** Normal lymphoma untreated group, **2.** DMBA induced lymphoma group untreated, **3.** DMBA induced lymphoma group subjected to CPL-Micelles NP without activation, **4.** DMBA induced lymphoma group subjected to laser only, **5.** DMBA induced lymphoma group subjected to laser in presence of CPL-Micelles NP, **6.** DMBA induced lymphoma group subjected to ultrasound only, **7.** DMBA induced lymphoma group subjected to ultrasound in presence of CPL-Micelles NP, **8.** DMBA induced lymphoma group subjected to combined modalities laser/ultrasound only, **9.** DMBA induced lymphoma group subjected to combined modalities laser/ultrasound in presence of CPL-Micelles NP.
